# SMC Abca1 and Abcg1 Deficiency Enhances Urinary Bladder Distension but Not Atherosclerosis

**DOI:** 10.1161/CIRCRESAHA.124.325103

**Published:** 2025-02-11

**Authors:** Benedek Halmos, Anouk M. La Rose, Daisey Methorst, Anouk G. Groenen, Dalibor Nakládal, Venetia Bazioti, Mirjam H. Koster, Niels J. Kloosterhuis, Azuwerus van Buiten, Elisabeth M. Schouten, Nicolette C.A. Huijkman, Miriam Langelaar-Makkinje, Laura Bongiovanni, Simon M. De Neck, Alain de Bruin, Hendrik Buikema, Leo E. Deelman, Marius C. van den Heuvel, Folkert Kuipers, Igle Jan de Jong, Judith C. Sluimer, Helle F. Jørgensen, Robert H. Henning, Marit Westerterp

**Affiliations:** Department of Pediatrics (B.H., A.M.L.R., D.M., A.G.G., V.B., M.H.K., N.J.K., N.C.A.H., M.L.-M., L.B., S.M.D.N., A.d.B., F.K., M.W.), University Medical Center Groningen, University of Groningen, the Netherlands.; Department of Clinical Pharmacy and Pharmacology (D.N., A.v.B., H.B., L.E.D., R.H.H.), University Medical Center Groningen, University of Groningen, the Netherlands.; Department of Cardiology (E.M.S.), University Medical Center Groningen, University of Groningen, the Netherlands.; Department of Pathology (M.C.v.d.H.), University Medical Center Groningen, University of Groningen, the Netherlands.; Department of Laboratory Medicine (F.K.), University Medical Center Groningen, University of Groningen, the Netherlands.; Department of Urology (I.J.d.J.), University Medical Center Groningen, University of Groningen, the Netherlands.; Comenius University Science Park, Bratislava, Slovakia (D.N.).; 5th Department of Internal Medicine, Faculty of Medicine, Comenius University Bratislava, Slovakia (D.N.).; Department of Biomolecular Health Sciences, Dutch Molecular Pathology Center, University of Utrecht, the Netherlands (L.B., S.M.D.N., A.d.B.).; Department of Veterinary Medicine, University of Teramo, Italy (L.B.).; Department of Pathology, Cardiovascular Research Institute Maastricht, Maastricht University Medical Center, the Netherlands (J.C.S.).; Department of Medical Clinic II for Kidney and Hypertension Diseases, Rheumatological and Immunological Diseases, Rheinisch-Westfälische Technische Hochschule (RWTH) Aachen, Germany (J.C.S.).; British Heart Foundation (BHF) Centre for Cardiovascular Sciences, University of Edinburgh, United Kingdom (J.C.S.).; Section of Cardiorespiratory Medicine, Department of Medicine, University of Cambridge, Cambridge Biomedical Campus, United Kingdom (H.F.J.).

**Keywords:** atherosclerosis, cell transdifferentiation, cholesterol, myocytes, smooth muscle, vasoconstriction

## Abstract

**BACKGROUND:**

Smooth muscle cells (SMCs) regulate blood flow distribution via vasoconstriction mediated by α-ARs (α-adrenergic receptors). Plasma membrane cholesterol accumulation affects α_1_-AR signaling and promotes loss of SMC contractile markers in vitro. ABCA1 and ABCG1 (ATP-binding cassette transporter A1 and G1) mediate cholesterol efflux to HDL (high-density lipoprotein). ABCA1/ABCG1 show high expression in medial and low expression in intimal SMCs of atherosclerotic plaques. The role of ABCA1 and ABCG1 in SMC-mediated vasoconstriction and atherogenesis remains poorly understood.

**METHODS:**

We generated mice with SMC-specific *Abca1/Abcg1* deficiency on the low-density lipoprotein receptor–deficient (*Ldlr*^*−*^^*/*^^*−*^) background by crossbreeding *Abca1*^*fl/fl*^*Abcg1*^*fl/fl*^*Ldlr*^*−/−*^ mice with *Myh11Cre*^*ERT2*^ transgenic mice. To induce SMC cholesterol accumulation and atherogenesis, we fed *Myh11Cre*^*ERT2*^*Abca1*^*fl/fl*^*Abcg1*^*fl/fl*^*Ldlr*^*−/−*^, *Myh11Cre*^*ERT2*^*Abca1*^*fl/fl*^*Ldlr*^*−/−*^, *Myh11Cre*^*ERT2*^*Abcg1*^*fl/fl*^*Ldlr*^*−/−*^, and *Myh11Cre*^*ERT2*^*Ldlr*^*−/−*^ mice Western-type diet for 16 weeks.

**RESULTS:**

Combined *SMC-Abca1/Abcg1* deficiency increased vasoconstriction in aortic rings induced by the α_1_-AR agonist phenylephrine. Unexpectedly, *SMC-Abca1/Abcg1* deficiency induced urinary bladder distension by >20-fold. This was reversed by the α_1_-AR antagonist tamsulosin, indicating its dependence on bladder neck SMC constriction. Moreover, *SMC-Abca1/Abcg1* deficiency decreased contractile markers and increased macrophage and fibroblast markers in bladder SMCs, indicating SMC transdifferentiation. This was accompanied by free cholesterol accumulation and increased endoplasmic reticulum stress. *SMC-Abca1/Abcg1* deficiency did not induce thoracic aorta SMC transdifferentiation, presumably due to increased cholesteryl ester accumulation and no endoplasmic reticulum stress in thoracic aorta SMCs. Surprisingly, *SMC-Abca1/Abcg1* deficiency did not affect atherosclerotic lesion size or composition in the aortic root or brachiocephalic artery.

**CONCLUSIONS:**

We uncover a new role of SMC cholesterol efflux pathways in suppressing α_1_-AR–mediated vasoconstriction and bladder SMC transdifferentiation, decreasing urinary bladder distension. Our data may provide a mechanistic link for the association between urinary bladder distension and diabetes in humans, particularly because diabetes is associated with decreased cholesterol efflux. *SMC-Abca1/Abcg1* deficiency did not affect atherosclerotic lesion size or plaque composition, presumably due to low expression of *Abca1/Abcg1* in intimal SMCs.

**Novelty and Significance**

**What Is Known?**

- Plasma membrane cholesterol accumulation in rat fibroblasts increases α_1_-AR (α_1_-adrenergic receptor) signaling in vitro, but the significance of this observation for α_1_-AR–mediated constriction in vivo is unclear.
- Upon cholesterol loading, aortic smooth muscle cells (SMCs) lose their SMC contractile markers and gain expression of macrophage and fibroblast markers (SMC transdifferentiation) in vitro.
- SMCs show high expression of the cholesterol transporters ABCA1 and ABCG1 (ATP-binding cassette transporter A1 and G1) that mediate cholesterol efflux to apo AI and HDL (high-density lipoprotein), respectively. It is unknown whether these ABC-mediated cholesterol efflux pathways affect vasoconstriction or atherosclerosis.

**What New Information Does This Article Contribute?**

- *SMC-Abca1/Abcg1* deficiency in low-density lipoprotein receptor–deficient (*Ldlr*^*−/−*^) mice fed a western-type diet induces α_1_-AR–mediated vasoconstriction in aortic rings, due to plasma membrane cholesterol accumulation and, presumably, increased α_1_-AR surface expression.
- *SMC-Abca1/Abcg1* deficiency in *Ldlr*^*−/−*^ mice fed western-type diet does not affect thoracic aorta SMC transdifferentiation and does not induce formation of atherosclerotic plaques in the thoracic aorta while also not affecting atherosclerosis in the aortic root or brachiocephalic artery.
- *SMC-Abca1/Abcg1* deficiency in *Ldlr*^*−/−*^ mice fed western-type diet induces bladder SMC transdifferentiation and as a consequence thereof, and to a lesser extent α_1_-AR–mediated constriction, urinary bladder distension.

In vitro studies suggest that cholesterol accumulation in SMCs regulates α_1_-AR signaling and SMC dedifferentiation. ABCA1 and ABCG1 mediate cholesterol efflux to HDL and show high expression in medial and low expression in intimal SMCs of atherosclerotic plaques. We here investigated the role of ABCA1/ABCG1 in SMC-mediated vasoconstriction and atherosclerosis employing *SMC-Abca1/Abcg1* deficient *Ldlr*^*−/−*^ mice fed a cholesterol-rich western-type diet. SMC-*Abca1/Abcg1* deficiency increased free cholesterol and cholesteryl ester accumulation in thoracic aorta SMCs, as well as α_1_-AR–mediated vasoconstriction in aortic rings. SMC-*Abca1/Abcg1* deficiency did not affect atherosclerosis in the aortic root or brachiocephalic artery, presumably due to low Abca1/Abcg1 expression in intimal SMCs, as shown by previous studies. SMC-*Abca1/Abcg1* deficiency did not induce atherosclerosis in the thoracic aorta, consistent with SMC*-Abca1/Abcg1* deficiency not inducing thoracic aorta transdifferentiation or endoplasmic reticulum stress. Unexpectedly, SMC-*Abca1/Abcg1* deficiency induced urinary bladder distension. Although partially dependent on α_1_-AR–mediated constriction, we mainly attributed this to loss of contractile markers and endoplasmic reticulum stress in bladder SMCs. We attribute the effect of SMC-*Abca1/Abcg1* deficiency on bladder, but not thoracic aorta SMC transdifferentiation to bladder SMCs accumulating free, but not esterified cholesterol, while thoracic aorta SMCs accumulate both. These studies provide new insights into the effects of ABCA1/ABCG1 on SMC function.

**Meet the First Author, see p 454**

Vascular smooth muscle cells (SMCs) are the effectors of contraction of the vascular wall, as such regulating blood flow distribution and blood pressure.^1^ The α_1_-AR (α_1_-adrenergic receptor) is highly expressed by vascular SMCs^2^ and stimulates Ca^2+^ release from internal stores and Ca^2+^ influx, which promotes SMC contraction and vasoconstriction.^2,3^ Among the 3 α_1_-AR subtypes (α_1a_-AR, α_1b_-AR, and α_1d_-AR) that have been identified, α_1a_-AR is the main α_1_-AR subtype in human blood vessels.^4^ In vitro studies employing α_1a_-AR overexpression in rat fibroblasts have shown that cholesterol accumulation, especially in the plasma membrane, increases the interaction of the α_1a_-AR with its G-protein effectors, which increases α_1a_-AR signaling.^5^ Consistently, in the same cell system, membrane cholesterol depletion by methyl-β-cyclodextrin (MβCD) decreased α_1a_-AR signaling, at high doses of the α_1_-AR agonist phenylephrine.^6^ SMCs show high expression of the cholesterol transporters ABCA1 and ABCG1 (ATP-binding cassette transporter A1 and G1) that mediate cholesterol efflux to apo AI and HDL (high-density lipoprotein), respectively.^7–11^ The role of these cholesterol efflux pathways in α_1_-AR–mediated SMC vasoconstriction is unknown.

In addition to mediating vasoconstriction, aortic SMCs play a major role in maintaining atherosclerotic plaque stability.^12,13^ During atherosclerosis, contractile SMCs migrate from the media into the intima and acquire a synthetic phenotype, promoting SMC proliferation and production of extracellular matrix, including collagen, to form a stable fibrous cap.^1,12,13^ In mice, lineage tracing and single-cell RNA-sequencing studies revealed that within the plaque, several cell types are from Myh11^+^ (myosin heavy chain 11) SMC origin, including macrophage-like cells, mesenchymal stem cells, osteogenic-like cells, myofibroblasts,^14^ Sox9^+^ (SRY-box transcription factor 9) chondrocyte-like cells,^15^ and Lumican^+^ fibromyocytes;^16^ the latter 3 being fibroblast-like.^14–18^ Recent studies show that SMCs first differentiate into an intermediate state, referred to as multipotent stem cell endothelial cell monocyte (SEM),^15,19^ or a transitional Lgals3^+^ or Mac-2^+^ (galectin-3^+^) SMC state,^17^ before acquiring markers of other cell types.^15,17^ Also in plaques from human carotids, an SEM cell state has been identified,^15^ suggesting translational relevance of the findings in mice. In addition, several studies suggest that in humans, SMCs are the most abundant cell type in atherosclerotic plaques and are already present in athero-prone arteries in utero before signs of atherosclerosis develop, a phenomenon referred to as diffuse intimal thickening.^20,21^ These SMCs accumulate lipids.^22^ It has been suggested that up to 50% of foam cells in human coronary atherosclerotic plaques originate from SMCs and that these SMCs have acquired macrophage markers.^11,23^

Several pathways regulate SMC transdifferentiation processes. The transcription factor Klf4 (Krüppel-like factor 4) stimulates the differentiation of SMCs into Lgals3^+^ SMCs, macrophage-like cells, and to a lesser extent into the other cell types.^14,17^ Tcf21 (transcription factor 21) stimulates differentiation of SMCs into fibromyocytes,^16^ and agonism of retinoic acid receptors via all-trans retinoic acid inhibits differentiation of SMCs into SEM cells.^15^ SMC-*Klf4* deficiency and treatment of LDL (low-density lipoprotein) receptor–deficient (*Ldlr*^*−/−*^) mice with all-trans retinoic acid decrease lesion size and plaque stability,^15–17^ suggesting that differentiation of SMCs into Lgals3^+^ SMCs, macrophage-like cells, or SEM cells is proatherogenic. Interestingly, in vitro studies have shown that SMC cholesterol loading increases expression of macrophage markers concomitant with upregulation of Klf4 and downregulation of microRNA-143/145.^14,24^ A more recent study has shown that SMC cholesterol accumulation increases expression of macrophage and fibroblast markers downstream of endoplasmic reticulum (ER) stress.^25^ Although these data strongly suggest that cholesterol accumulation enhances SMC transdifferentiation in vitro,^14,15,24^ the role of SMC ABCA1- and ABCG1-mediated cholesterol efflux pathways in atherosclerosis has not been investigated directly. A role for these cholesterol efflux pathways in SMC transdifferentiation in the intima has been suggested. Intimal SMCs in human atherosclerotic plaques from coronaries show low *ABCA1* mRNA expression compared with medial SMCs,^10^ a finding that has been suggested to contribute to SMCs gaining markers of macrophages and becoming foam cells in atherosclerotic plaques.^11^ Furthermore, more recent studies in mice revealed low Abca1 expression in intimal CD45^−^ cells versus intimal CD45^+^ cells and also suggested that in mice, low Abca1 expression in intimal SMCs may contribute to foam cell formation.^26^ Although these studies suggest that expression of Abca1 in intimal SMCs may be low,^10,26^ residual expression of Abca1 in intimal SMCs may still contribute to atherogenesis. In addition, recent studies suggest that ER stress downstream of cholesterol accumulation in medial SMCs contributes to SMC migration,^27^ and thus cholesterol efflux by Abca1 and Abcg1 in medial SMCs may affect atherogenesis.

Deficiency of *Abca1* and *Abcg1* in macrophages,^28,29^ endothelial cells,^30^ dendritic cells,^31^ or hematopoietic stem and progenitor cells^32^ leads to marked cholesterol accumulation in these cells. We here generated mice with deficiency of *Abca1* and *Abcg1* in SMCs as a model of defective SMC cholesterol efflux and investigated the role of SMC cholesterol efflux pathways in vasoconstriction and atherogenesis.

## Methods

Detailed methods are provided in the Supplemental Material.

### Data Availability

All supporting data are available within the article and the Supplemental Material.

## Results

### SMC *Abca1/Abcg1* Deficiency Increases Lipid Accumulation in Aortic SMCs of *Ldlr*^*−/−*^ Mice Fed Western-Type Diet

**Figure 1.**
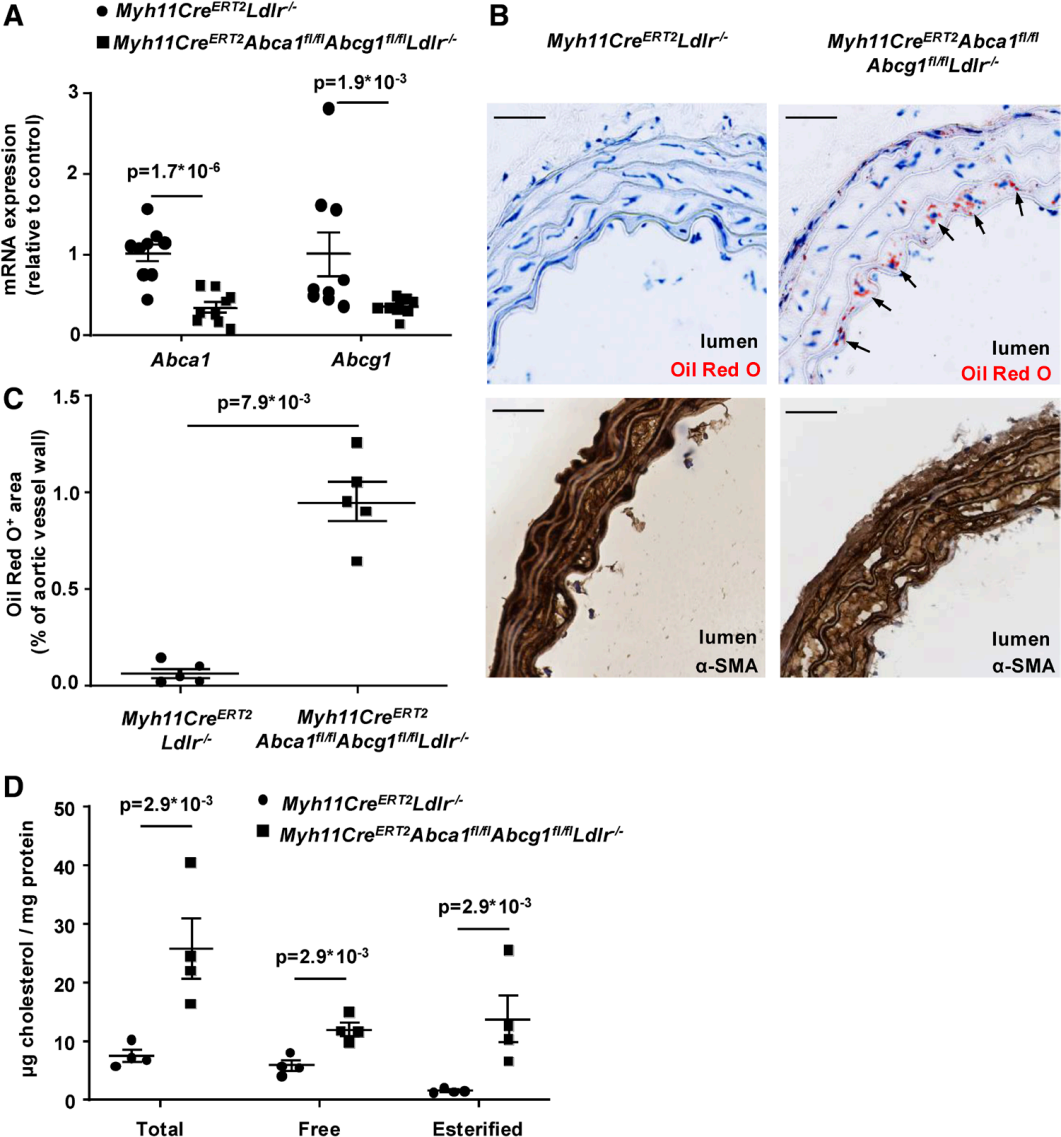
***Myh11Cre*^*ERT2*^*Abca1*^*fl/fl*^*Abcg1*^*fl/fl*^*Ldlr*^*−/−*^ mice show reduced *Abca1* and *Abcg1* mRNA expression and increased lipid accumulation in smooth muscle cells of the thoracic aorta compared with their controls.** *Myh11Cre*^*ERT2*^*Ldlr*^*−/−*^ and *Myh11Cre*^*ERT2*^*Abca1*^*fl/fl*^*Abcg1*^*fl/fl*^*Ldlr*^*−/−*^ mice were fed a tamoxifen diet for 1 week, followed by chow diet for 2 weeks, and western-type diet (WTD) for 16 weeks. **A**, Thoracic aortas were isolated, digested, and *Abca1* and *Abcg1* mRNA expression was determined (n=9). **B**, Thoracic aortas were sectioned and stained with Oil Red O and for α-SMA (α-smooth muscle actin). Arrowheads indicate Oil Red O positive areas. Scale bar represents 200 µm. **C**, Quantification of Oil Red O–positive areas corrected for the total area of the aortic section (n=5). **D**, Thoracic aorta total and free cholesterol were measured by gas chromatography–mass spectrometry. Esterified cholesterol=total cholesterol−free cholesterol (n=4). Data are shown as mean±SEM. *P*<0.05 by 2-tailed unpaired *t* test (**A**, *Abca1*) or Mann-Whitney *U* test (**A**, *Abcg1*; **C** and **D**) are indicated.

To generate a mouse model with defective SMC cholesterol efflux, we bred mice with SMC *Abca1* and *Abcg1* deficiency on the *Ldlr*^*−/−*^ background. Using the SMC-specific *Myh11Cre*^*ERT2*^ promoter, we generated *Myh11Cre*^*ERT2*^*Abca1*^*fl/fl*^*Abcg1*^*fl/fl*^*Ldlr*^*−/−*^ mice and *Myh11Cre*^*ERT2*^*Ldlr*^*−/−*^ controls. Mice were fed a diet containing tamoxifen for 1 week to induce activation of *Myh11Cre*^*ERT2*^, resulting in a ≈60% to 70% decrease of both *Abca1* and *Abcg1* mRNA expression in aortic SMCs of *Myh11Cre*^*ERT2*^*Abca1*^*fl/fl*^*Abcg1*^*fl/fl*^*Ldlr*^*−/−*^ compared with *Myh11Cre*^*ERT2*^*Ldlr*^*−/−*^ mice (Figure 1A). Of note, *Abca1* mRNA expression was >100-fold higher than *Abcg1* mRNA expression in aortic SMCs from *Myh11Cre*^*ERT2*^*Ldlr*^*−/−*^ mice (results not shown). The decrease of ≈60% to 70% in the expression of floxed genes is similar to previous studies using the *Myh11Cre*^*ERT2*^ promoter and likely the consequence of not all aortic SMCs expressing the *Myh11Cre*^*ERT2*^ transgene.^14,33^ We refer to *Myh11Cre*^*ERT2*^*Abca1*^*fl/fl*^*Abcg1*^*fl/fl*^*Ldlr*^*−/−*^ and *Myh11Cre*^*ERT2*^*Ldlr*^*−/−*^ mice as *SMC-Abc*^*dko*^*Ldlr*^*−/−*^ and *Ldlr*^*−/−*^ mice, respectively. Using similar procedures, *SMC-Abca1*^*ko*^*Ldlr*^*−/−*^ and *SMC-Abcg1*^*ko*^*Ldlr*^*−/−*^ mice were generated. We fed these mice a cholesterol-rich western-type diet (WTD) for 16 weeks, which induced hypercholesterolemia to a similar level (≈900 mg/dL) in all models (Table S1). Over the course of WTD feeding, *SMC-Abc*^*dko*^*Ldlr*^*−/−*^ and *Ldlr*^*−/−*^ mice gained bodyweight in a similar fashion (Table S1; Figure S1). After 16 weeks of WTD feeding, *SMC-Abc*^*dko*^*Ldlr*^*−/−*^ mice showed >10-fold increase in Oil Red O staining in α-SMA (α-smooth muscle actin)–positive SMCs of the thoracic aorta compared with *Ldlr*^*−/−*^ controls (Figure 1B and 1C), reflecting neutral lipid accumulation. These sections of the thoracic aorta did not show Mac-2 staining (Figure S2), indicating that the increase in Oil Red O was not the consequence of monocytes infiltrating into the vascular wall. In line with the data on Oil Red O staining, in aortic SMCs, *SMC-Abca1/Abcg1* deficiency increased cholesteryl ester (CE) accumulation by >8-fold (Figure 1D). *SMC-Abca1/Abcg1* deficiency increased free cholesterol accumulation by 2-fold (Figure 1D).

### *SMC-Abca1*/*Abcg1* Deficiency Enhances Phenylephrine-Induced Vasoconstriction in Aortic Rings From *Ldlr*^*−/−*^ Mice Fed WTD, Which Is Dependent on Membrane Cholesterol

**Figure 2.**
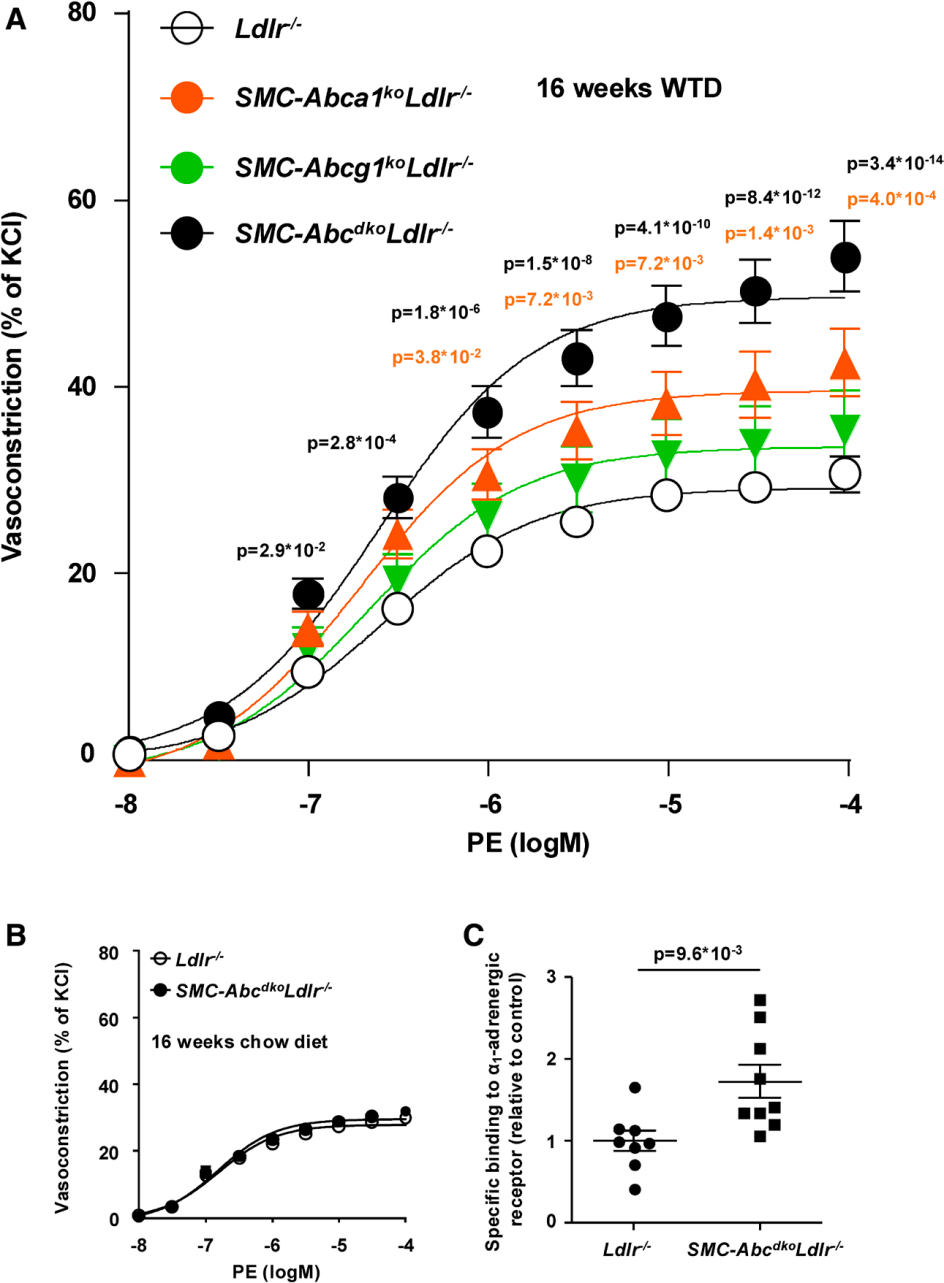
**Smooth muscle cell (SMC) Abca1/*Abcg1* deficiency increases the binding of ligands to the α_1_-AR (α1-adrenergic receptor) and enhances phenylephrine (PE)-induced vasoconstriction in aortic rings from *Ldlr*^*−/−*^ mice fed western-type diet (WTD).** Mice were fed WTD (**A** and **C**) or chow diet for 16 weeks (**B**). **A** and **B**, Aortic rings were isolated, mounted on a wire myograph, and subjected to increasing concentrations of PE. Vasoconstriction is expressed as % of maximum KCl-induced vasoconstriction. **A**, PE-induced vasoconstriction in aortic rings from WTD-fed *Ldlr*^*−/−*^ (n=20), *SMC-Abca1*^*ko*^*Ldlr*^*−/−*^ (n=7), *SMC-Abcg1*^*ko*^*Ldlr*^*−/−*^ (n=6), and *SMC-Abc*^*dko*^*Ldlr*^*−/−*^ (n=16). First, vasoconstriction was examined in aortic rings of *Ldlr*^*−/−*^ vs *SMC-Abc*^*dko*^*Ldlr*^*−/−*^ mice and then in aortic rings of *Ldlr*^*−/−*^ vs *SMC-Abca1*^*ko*^*Ldlr*^*−/−*^ and *Ldlr*^*−/−*^ vs *SMC-Abcg1*^*ko*^*Ldlr*^*−/−*^ mice. In all experiments employing aortic rings from *SMC-Abca1*^*ko*^*Ldlr*^*−/−*^ and *SMC-Abcg1*^*ko*^*Ldlr*^*−/−*^ mice, aortic rings from *SMC-Abc*^*dko*^*Ldlr*^*−/−*^ mice were used as positive controls. Therefore, the data of the 4 groups are presented together (**A**). **B**, PE-induced vasoconstriction in chow diet–fed *Ldlr*^*−/−*^ and *SMC-Abc*^*dko*^*Ldlr*^*−/−*^ mice (n=6). **C**, α_1_-AR–specific binding in thoracic aortic SMCs from WTD-fed *Ldlr*^*−/−*^ (n=8) and *SMC-Abc*^*dko*^*Ldlr*^*−/−*^ (n=9) mice (*P*=9.6×10^−^3; 2-tailed unpaired *t* test) was assessed using a competitive radioligand binding assay employing [7-methoxy-^3^H]-prazosin and phentolamine as described in the methods section. Data are shown as mean±SEM. *P*<0.05 by 2-way ANOVA with Sidak’s multiple comparison post-test (**A**) are indicated for comparisons between *SMC-Abc*^*dko*^*Ldlr*^*−/−*^ and *Ldlr*^*−/−*^ mice (black) or *SMC-Abca1*^*ko*^*Ldlr*^*−/−*^ and *Ldlr*^*−/−*^ mice (orange). For (**C**), *P* value by 2-tailed unpaired *t* test is indicated. KCl, potassium chloride.

We then assessed the effect of *SMC-Abca1/Abcg1* deficiency on vascular function in *Ldlr*^*−/−*^ mice. Phenylephrine induces vasoconstriction by binding to the α_1_-AR,^34^ and in vitro studies have shown that membrane cholesterol accumulation may affect this α_1_-AR–mediated response.^5,6^ To investigate whether *SMC-Abca1/Abcg1* deficiency affects vascular function, we measured α_1_-AR–mediated vasoconstriction in aortic rings isolated from *Ldlr*^−/−^, *SMC-Abca1*^*ko*^*Ldlr*^*−/−*^, *SMC-Abcg1*^*ko*^*Ldlr*^*−/−*^, and *SMC-Abc*^*dko*^*Ldlr*^*−/−*^ mice. These mice were fed a WTD for 16 weeks to enhance cholesterol accumulation in aortic SMCs. Although combined *SMC-Abca1/Abcg1* deficiency increased maximum vasoconstriction to phenylephrine in aortic rings from *Ldlr*^*−/−*^ mice by 1.8-fold, deficiency of *SMC-Abca1* alone increased phenylephrine-induced vasoconstriction by 1.3-fold, and *SMC-Abcg1* deficiency showed no effect (Figure 2A). When *SMC-Abca1/Abcg1* deficient mice were kept on chow diet, phenylephrine-induced vasoconstriction was not affected (Figure 2B), presumably because of insufficient SMC cholesterol accumulation.

To examine whether the effect of *SMC-Abca1/Abcg1* deficiency on phenylephrine-induced vasoconstriction was membrane cholesterol-dependent, we induced membrane cholesterol depletion from aortic rings using 10 mmol/L MβCD for 45 min, a condition previously shown to decrease the interaction between the α_1_-AR and its G-protein effectors.^5^ Preincubation with MβCD suppressed the maximum phenylephrine-induced vasoconstriction in aortic rings from *Ldlr*^*−/−*^ and *SMC-Abc*^*dko*^*Ldlr*^*−/−*^ mice fed WTD (Figure S3A and S3B) by 64% and 30%, respectively, indicating a key role for membrane cholesterol in regulating phenylephrine-mediated effects in the aorta. The observation that MβCD treatment did not completely normalize phenylephrine-induced vasoconstriction in aortic rings from *SMC-Abc*^*dko*^*Ldlr*^*−/−*^ mice is likely due to increased accumulation of SMC membrane cholesterol in *SMC-Abc*^*dko*^*Ldlr*^*−/−*^ compared with *Ldlr*^*−/−*^ mice and membrane cholesterol not being removed completely by MβCD. The EC_50_ of phenylephrine was unaffected in all experiments (Table S2). Despite increased α_1_-AR–mediated vasoconstriction, we did not observe any effect of *SMC-Abca1/Abcg1* deficiency on blood pressure in the aorta and left ventricle (Table S3). *SMC-Abca1/Abcg1* deficiency also did not affect endothelium-dependent acetylcholine-induced or sodium nitroprusside-induced vasorelaxation in aortic rings from *Ldlr*^*−/−*^ mice fed WTD (Figure S3C and S3D). Importantly, membrane cholesterol depletion by MβCD did not affect vasoconstriction downstream of the G-protein coupled receptors 5-hydroxytryptamine_2_ and the thromboxane A_2_ receptor in SMCs (Figure S3E and S3F). These data demonstrate that membrane cholesterol accumulation is highly specific for the regulation of α_1_-AR–mediated vasoconstriction, rather than representing a common mechanism regulating G-protein coupled receptor–mediated vasoconstriction in SMCs.

Therefore, we next assessed whether *SMC-Abca1/Abcg1* deficiency increased specific ligand binding to α_1_-AR in SMCs isolated from the thoracic aorta of WTD-fed *Ldlr*^*−/−*^ and *SMC-Abc*^*dko*^*Ldlr*^*−/−*^ mice. Using a competitive receptor binding assay employing the α_1_-AR ligands radiolabeled prazosin and unlabeled phentolamine, we found that *SMC-Abca1/Abcg1* deficiency increased specific binding to the α_1_-AR (Figure 2C), indicating increased plasma membrane levels of α_1_-AR in the absence of cholesterol efflux pathways. *SMC-Abca1/Abcg1* deficiency did not affect *α*_*1a*_*-AR* mRNA expression in aortic SMCs (results not shown).

To further study cholesterol efflux pathways in relation to α_1a_-AR surface expression and downstream signaling, we performed the opposite experiment by stimulating Abca1- and Abcg1-mediated cholesterol efflux by rHDL (reconstituted HDL) that depletes cell membrane cholesterol.^35^ We transfected HEK293T (human embryonic kidney 293T) cells with the ADRA1A (adrenergic receptor-α_1a_)-Tango plasmid expressing human α_1a_-AR with a FLAG epitope in its N terminus allowing for the detection of α_1a_-AR cell surface expression. Over a period of 24 hours, rHDL decreased α_1a_-AR cell surface expression by ≈50%, whereas phenylephrine did not affect it (Figure S4A through S4E). Similarly, rHDL also decreased total (surface+intracellular) α_1a_-AR cell surface expression over the same period of time, as assessed after fixation and permeabilization (Figure S4F and S4G). We then studied ERK (extracellular signal–regulated kinase) phosphorylation that occurs downstream of the α_1a_-AR.^30^ We stimulated HEK293T cells transfected with the α_1a_-AR with the same concentration of phenylephrine as used for flow cytometry experiments, in the absence or presence of rHDL. Phenylephrine stimulation increased phosphorylation of ERK, which was suppressed by rHDL (Figure S4H and S4I), suggesting, consistent with previous studies employing methyl-β-cyclodextrin,^36^ and our vasoconstriction studies, that cholesterol efflux pathways suppress α_1a_-AR signaling. These data indicate a direct link between membrane cholesterol accumulation, α_1a_-AR surface expression, and downstream signaling.

To examine whether rHDL also affected the surface expression of other G-coupled protein receptors that control vascular function, we examined the effects of rHDL on the AGTR1 (angiotensin II type receptor 1). We transfected HEK293A cells with the AGTR1-Tango plasmid expressing human AGTR1 with a FLAG epitope in its N terminus allowing for the detection of AGTR1 cell surface expression. Although angiotensin II incubation decreased AGTR1 surface expression, indicative of AGTR1 internalization, rHDL did not affect it (Figure S4J and S4K), substantiating our earlier observations (Figure S3E and S3F) that membrane cholesterol depletion is highly specific for regulation of the α_1a_-AR, rather than a common mechanism regulating G-protein coupled receptors.

Collectively, *SMC-Abca1*/*Abcg1* deficiency specifically increased phenylephrine-induced vasoconstriction in aortic rings from WTD-fed *Ldlr*^*−/−*^ mice, which was reversed by preincubation with MβCD, supporting its membrane cholesterol dependency. In addition, increased radioligand binding suggests that *SMC-Abca1/Abcg1* deficiency increases α_1_-AR plasma membrane abundance. The latter was substantiated by rHDL decreasing α_1a_-AR surface expression and downstream signaling in HEK293T cells expressing the α_1a_-AR, likely due to increased cholesterol efflux.

### *SMC-Abca1/Abcg1* Deficiency Increases Urinary Bladder Volume in *Ldlr*^*−/−*^ Mice Fed WTD

**Figure 3.**
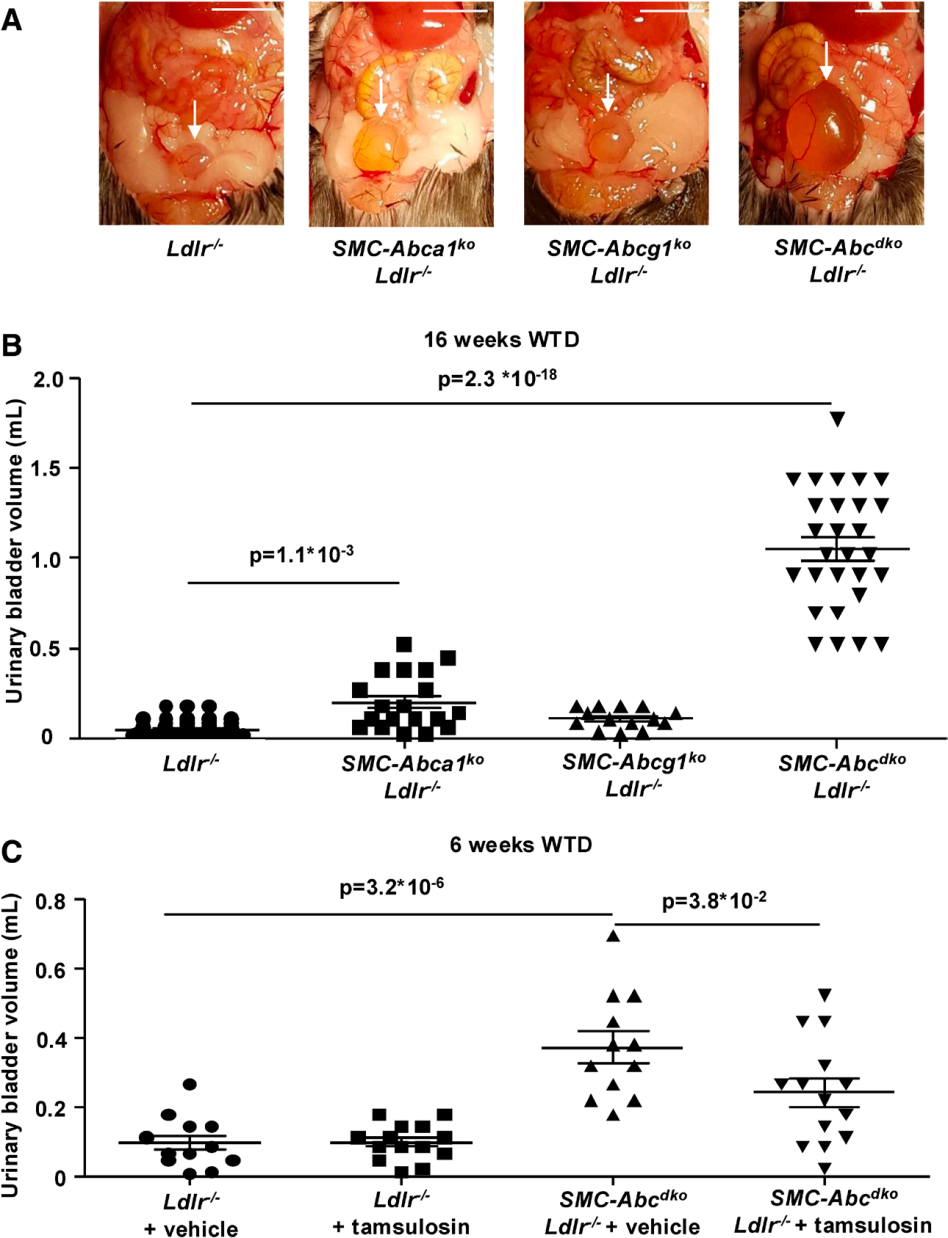
**Effects of smooth muscle cell (SMC) *Abca1/Abcg1* deficiency on the urinary bladder in *Ldlr*^*−/−*^ mice fed western-type diet (WTD), with partial reversal by the α_1_-AR (α1-adrenergic receptor) antagonist tamsulosin.** *Ldlr*^*−/−*^, *SMC-Abca1*^*ko*^*Ldlr*^*−/−*^, *SMC-Abcg1*^*ko*^*Ldlr*^*−/−*^, and *SMC-Abc*^*dko*^*Ldlr*^*−/−*^ mice were fed WTD for 16 (**A** and **B**) or 6 (**C**) weeks. **A**, Representative images of the urinary bladder at 16 weeks of WTD feeding are shown. Scale bar represents 1 cm. **B**, Urinary bladder volume assessed at 16 weeks of WTD feeding (*Ldlr*^*−/−*^ (n=61), *SMC-Abca1*^*ko*^*Ldlr*^*−/−*^ (n=19), *SMC-Abcg1*^*ko*^*Ldlr*^*−/−*^ (n=14), *SMC-Abc*^*dko*^*Ldlr*^*−/−*^ (n=29). **C**, Mice received 0.4 mg/kg tamsulosin or vehicle in the drinking water at 2 weeks after the start of WTD for a period of 4 weeks. Urinary bladder volume was assessed (*Ldlr*^*−/−*^+vehicle, *SMC-Abc*^*dko*^*Ldlr*^*−/−*^+vehicle (n=12), *Ldlr*^*−/−*^+tamsulosin, *SMC-Abc*^*dko*^*Ldlr*^*−/−*^+tamsulosin [n=14]). Scale bar represents 1 cm. Data are shown as mean±SEM. *P*<0.05 by Kruskal-Wallis test with Dunn post-test with Bonferroni adjustment (**B**) or 1-way ANOVA with Bonferroni post-test (**C**) are indicated.

Strikingly, after 16 weeks of WTD feeding, *SMC-Abca1/Abcg1* deficiency increased urinary bladder volume by >20-fold in *Ldlr*^*−/−*^ mice, whereas *SMC-Abca1* deficiency increased urinary bladder volume by 4-fold, and *SMC-Abcg1* deficiency showed no effect (Figure 3A and 3B). Thus, the effects on urinary bladder volume in the 3 mouse models are consistent with the observed effects on vasoconstriction (Figure 2A). On a chow diet, *SMC-Abca1/Abcg1* deficiency did not affect urinary bladder volume (Figure S5A). These data thus indicate a major role for SMC cholesterol efflux pathways in suppressing hypercholesterolemia-induced distension of the urinary bladder.

We observed that *SMC-Abca1/Abcg1* deficiency increased urinary bladder volume already after 6 weeks of WTD (Figure S5B), although to a lesser extent than at 16 weeks of WTD. Both prostate or bladder neck SMCs could have accounted for the urinary bladder distension. However, the phenotype was SMC specific and few SMCs are present in prostate in mice,^37^ suggesting that rather bladder neck SMCs were involved. Because the α_1_-AR is crucial for bladder neck SMC constriction,^38,39^ we investigated whether this phenotype was mediated by the α_1_-AR. *SMC-Abc*^*dko*^*Ldlr*^*−/−*^ and *Ldlr*^*−/−*^ mice received 0.4 mg/kg of the α_1_-AR antagonist tamsulosin or vehicle in the drinking water at 2 weeks of WTD for a period of 4 weeks. Tamsulosin decreased the urinary bladder volume in *SMC-Abc*^*dko*^*Ldlr*^*−/−*^ mice by ≈35% (Figure 3C), supporting a key role for the α_1_-AR in increasing bladder volume in WTD-fed *Ldlr*^*−/−*^ mice with *SMC-Abca1/Abcg1* deficiency. Collectively, these results indicate that the increase in urinary bladder volume in WTD-fed *SMC-Abc*^*dko*^*Ldlr*^*−/−*^ mice is mediated by α_1_-AR activity. Bladder distension was accompanied by an increase in interstitial inflammation in the kidney, although we observed no signs of kidney damage such as tubular or glomerular changes (Figure S6A and S6B). Likewise, plasma creatinine levels, a measure of kidney function, were not affected (Figure S6C).

### *SMC-Abca1*/*Abcg1* Deficiency Induces Urinary Bladder Wall Thinning and Induces Differentiation of SMCs into Macrophage-Like and Fibroblast-Like Cells in WTD-fed *Ldlr*^*−/−*^ Mice

**Figure 4.**
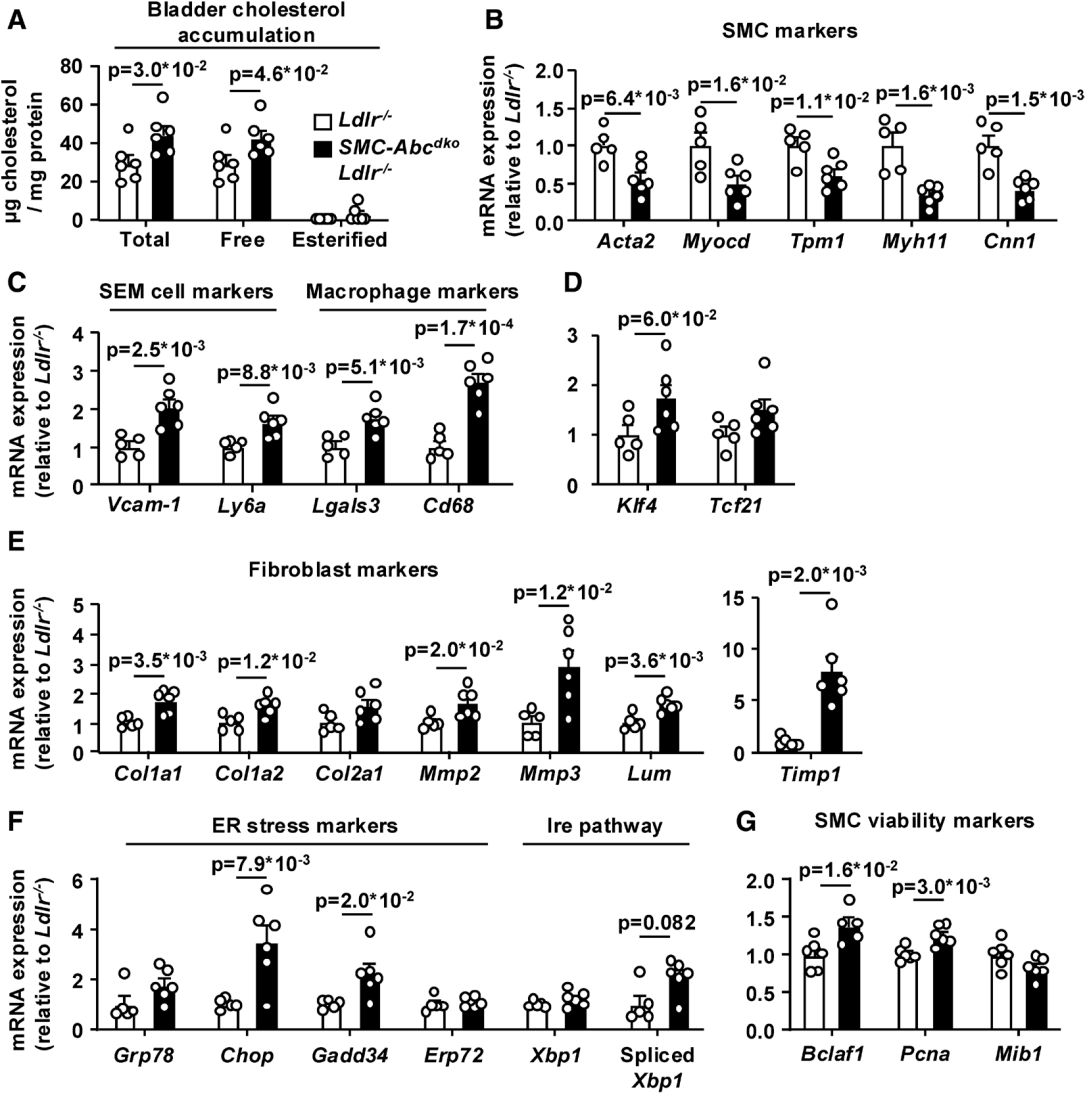
**Smooth muscle cell (SMC) *Abca1*/*Abcg1* deficiency induces free cholesterol accumulation, SMC differentiation into macrophage-like and fibroblast-like cells in the urinary bladder wall, and markers of endoplasmic reticulum (ER) stress in bladder SMCs.** *Ldlr*^*−/−*^ and *SMC-Abc*^*dko*^*Ldlr*^*−/−*^ mice were fed western-type diet (WTD) for 6 weeks. The urinary bladder was isolated, digested, and (**A**) total and free cholesterol were measured by gas chromatography–mass spectrometry. Esterified cholesterol=total cholesterol−free cholesterol (n=6). mRNA expression of (**B**) SMC markers, (**C**) stem cell endothelial cell monocyte (SEM) cell markers and macrophage markers, (**D**) Krüppel-like factor 4 (*Klf4*) and transcription factor 21 (*Tcf21*), (**E**) fibroblast markers, (**F**) ER stress markers (*Ldlr*^*−/−*^ [n=5], *SMC-Abc*^*dko*^*Ldlr*^*−/−*^ [n=6]), and (**G**) *Bclaf-1* and associated cell viability markers was determined (n=6, except for *Bclaf-1* for *SMC-Abc*^*dko*^ *Ldlr*^*−/−*^, where n=5 due to 1 undetermined CT value). Data are shown as mean±standard error of the mean. *P* values by 2-tailed unpaired *t* test are indicated.

**Figure 5.**
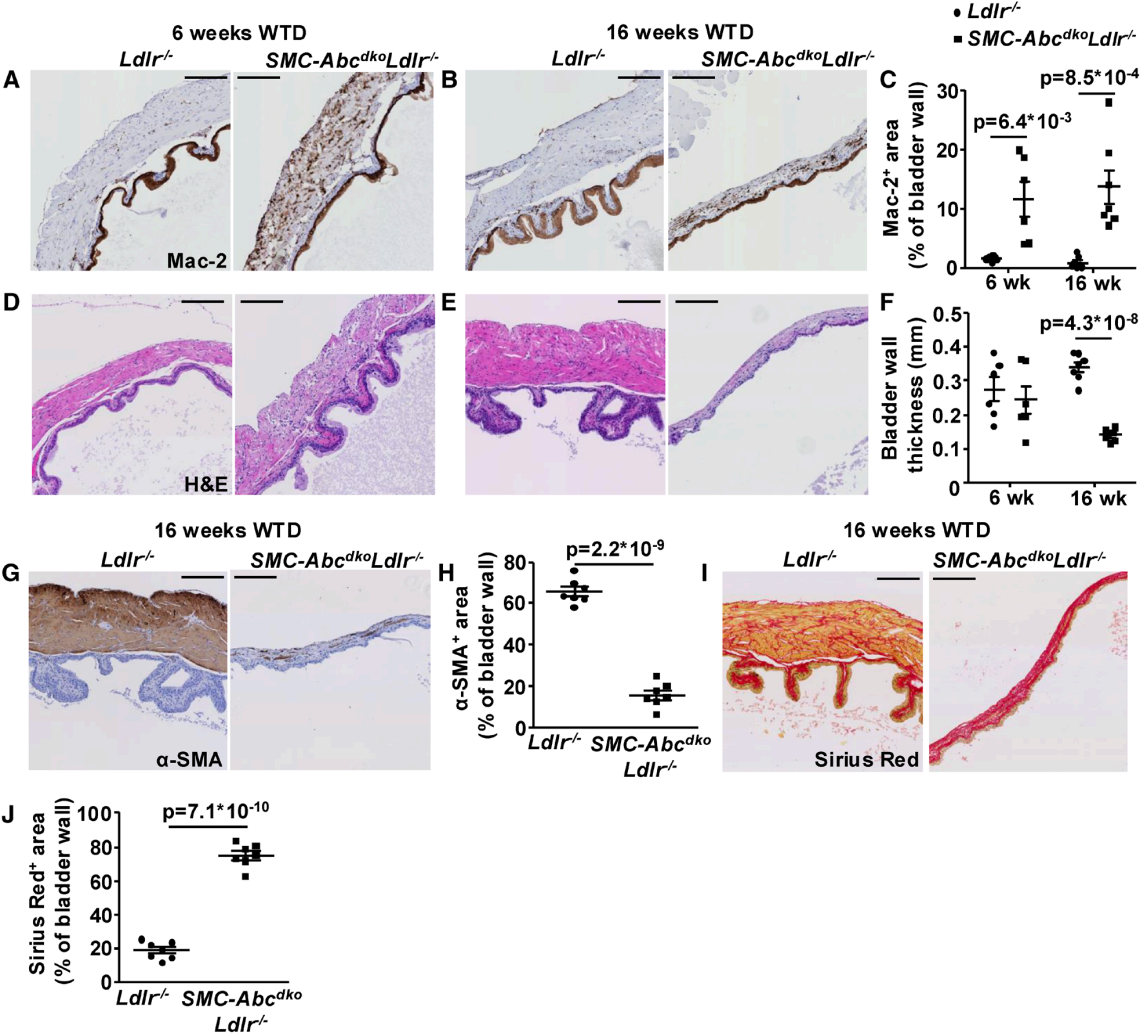
**Smooth muscle cell (SMC) *Abca1*/*Abcg1* deficiency induces urinary bladder wall thinning and induces SMC marker loss, and differentiation into Lgals3^+^ (or Mac-2; galectin-3+), macrophage-like and fibroblast-like cells.** *Ldlr*^*−/−*^ and *SMC-Abc*^*dko*^*Ldlr*^*−/−*^ mice were fed western-type diet (WTD) for 6 (**A** and **D**) or 16 weeks (**B**, **E**, **G**, and **J**). **A** through **C**, The urinary bladder was isolated, sectioned, and stained for Mac-2 (Lgals3^+^; **A** and **B**), and positive areas were quantified as % of bladder wall area (**C**). **D** through **F**, Sections were stained with hematoxylin and eosin (H&E). **F**, Quantification of urinary bladder wall thickness. **G** and **H**, Sections were stained for α-SMA (α-smooth muscle actin; **G**), and positive areas were quantified as % of bladder wall area (**H**). **I** and **J**, Sections were stained with Sirius Red (**I**), and positive areas were quantified as % of bladder wall area (n=7; **J**). For Mac-2 (Lgals3), the urothelium shows nonspecific staining. Scale bar represents 200 µm. Data are shown as mean±SEM. *P*<0.05 by 2-tailed unpaired *t* test (**C**, **F**, **H**, and **J**) are indicated.

We then further characterized the distended urinary bladder in *SMC-Abc*^*dko*^*Ldlr*^*−/−*^ mice. *SMC-Abca1/Abcg1* deficiency increased Sirius red staining, reflecting increased collagen content in the bladder wall of *Ldlr*^*−/−*^ mice at 6 weeks WTD; however, this was not reversed by the α_1_-AR antagonist tamsulosin (Figure S7A and S7B). Concomitantly, *SMC-Abca1/Abcg1* deficiency decreased α-SMA staining, a marker for SMCs, in the bladder wall of *Ldlr*^*−/−*^ mice after 6 weeks of WTD (Figure S7C and S7D), which was not reversed by the α_1_-AR antagonist tamsulosin (Figure S7C and S7D). This may be due to incomplete reversal of bladder distension at this tamsulosin dose. Alternatively, mechanisms independent of α_1_-AR activity may contribute to SMC dedifferentiation. Humans with rare, loss-of-function mutations in SMC contractile markers, including *ACTA2*, *MYH11*, or myocardin (*MYOCD*) show enlarged urinary bladders,^40–42^ a phenotype that was replicated at 4 months after inducing *SMC-Myocd* deficiency in *Myh11Cre*^*ERT2*^*Myocd*^*fl/fl*^ mice.^43^ We found that already within 7 days after inducing *SMC-Myocd* deficiency in *Myh11Cre*^*ERT2*^*Myocd*^*fl/fl*^ mice, *SMC-Myocd* deficiency led to a distended urinary bladder, accompanied by bladder wall thinning (Figure S8A through S8C), indicating that this phenotype develops rapidly and that loss of SMC markers is key to it. Previous studies have shown that SMCs in atherosclerotic plaques lose their contractile markers and acquire macrophage and fibroblast markers,^14–17^ preceded by differentiation into SEM cells^15^ or Lgals3^+^ SMCs.^17^ In vitro studies have shown that SMCs lose their markers and acquire macrophage and fibroblast markers secondary to cholesterol accumulation, which may be mediated by ER stress.^14,24,25^ *SMC-Abca1/Abcg1* deficiency increased accumulation of free cholesterol in bladder SMCs, whereas we could not detect CE (Figure 4A). Consistently, *SMC-Abca1/Abcg1* deficiency decreased mRNA expression of several SMC markers in the bladder (Figure 4B) and increased expression of the SEM cell markers, as well as the Lgals3^+^ SMC or macrophage marker *Lgals3* (*Mac-2*), and the macrophage marker *Cd68* (Figure 4C), while tending to increase mRNA expression of *Klf4* and *Tcf21* (Figure 4D), which mediate differentiation into macrophage-like and fibroblast-like cells, respectively. Substantiating differentiation of SMCs into macrophage-like cells, genes previously shown to be highly expressed by macrophage foam cells, associated with macrophage inflammation, induced by IFNγ (interferon gamma) or expressed by resident macrophages,^44–47^ were upregulated by *SMC-Abca1/Abcg1* deficiency (Figure S9A through S9E). *SMC-Abca1/Abcg1* deficiency barely affected the expression of osteoclast markers (Figure S9F) but increased the expression of several fibroblast markers (Figure 4E). Collectively, these data thus indicate that cholesterol accumulation enhances the transdifferentiation of SMCs into macrophage-like and fibroblast-like cells. *SMC-Abca1/Abcg1* deficiency also increased expression of ER stress markers and SMC viability markers (Figure 4F and 4G) that were recently shown to contribute to SMC differentiation into macrophage-like and fibroblast-like cells downstream of cholesterol accumulation.^25,48^ In addition, incubation of human bladder SMCs with cyclodextrin-cholesterol led to loss of SMC contractile markers and gain of macrophage markers as well as *KLF4* (Figure S10A through S10C), indicating human relevance of the findings in mice with *SMC-Abca1/Abcg1* deficiency. In support of our findings on mRNA expression, histological data revealed that *SMC-Abca1/Abcg1* deficiency increased staining of Mac-2 (Figure 5A through 5C). The collagen deposition in the bladder wall (Figure S7A and S7B) was likely due to increased differentiation into fibroblast-like cells.

After 16 weeks of WTD feeding, *SMC-Abca1/Abcg1* deficiency induced severe thinning of the bladder wall (Figure 5D through 5F), which was accompanied by further loss of α-SMA staining and increased Mac-2 staining, as well as a further increase in collagen deposition (Figure 5B, 5G through 5J). We did not find SMC apoptosis in the bladder wall, employing TUNEL staining (data not shown), and bladder SMCs only showed minimal proliferation (Figure S11) indicating that these mechanisms were unlikely to contribute to the distended urinary bladder on *SMC-Abca1/Abcg1* deficiency. Furthermore, the lumen area of the urethra, as well as SMC content and collagen deposition in the urethra were not affected by *SMC-Abca1/Abcg1* deficiency at 16 weeks of WTD (Figure S12A through S12F). Collectively, these findings show that in the setting of hypercholesterolemia, *SMC-Abca1/Abcg1* deficiency induces bladder wall SMC lipid accumulation, bladder wall thinning, loss of SMC markers, and an increase in Lgals3^+^ SMCs, macrophage-like, and fibroblast-like cells. In addition to α_1_-AR–mediated bladder neck SMC constriction, the SMC transdifferentiation into macrophage-like and fibroblast-like cells is a major contributor to the urinary bladder distension.

### *SMC-Abca1/Abcg1* Deficiency Does Not Affect Atherosclerotic Lesion Area, Composition, or Plaque Stability in *Ldlr*^*−/−*^ Mice Fed WTD

**Figure 6.**
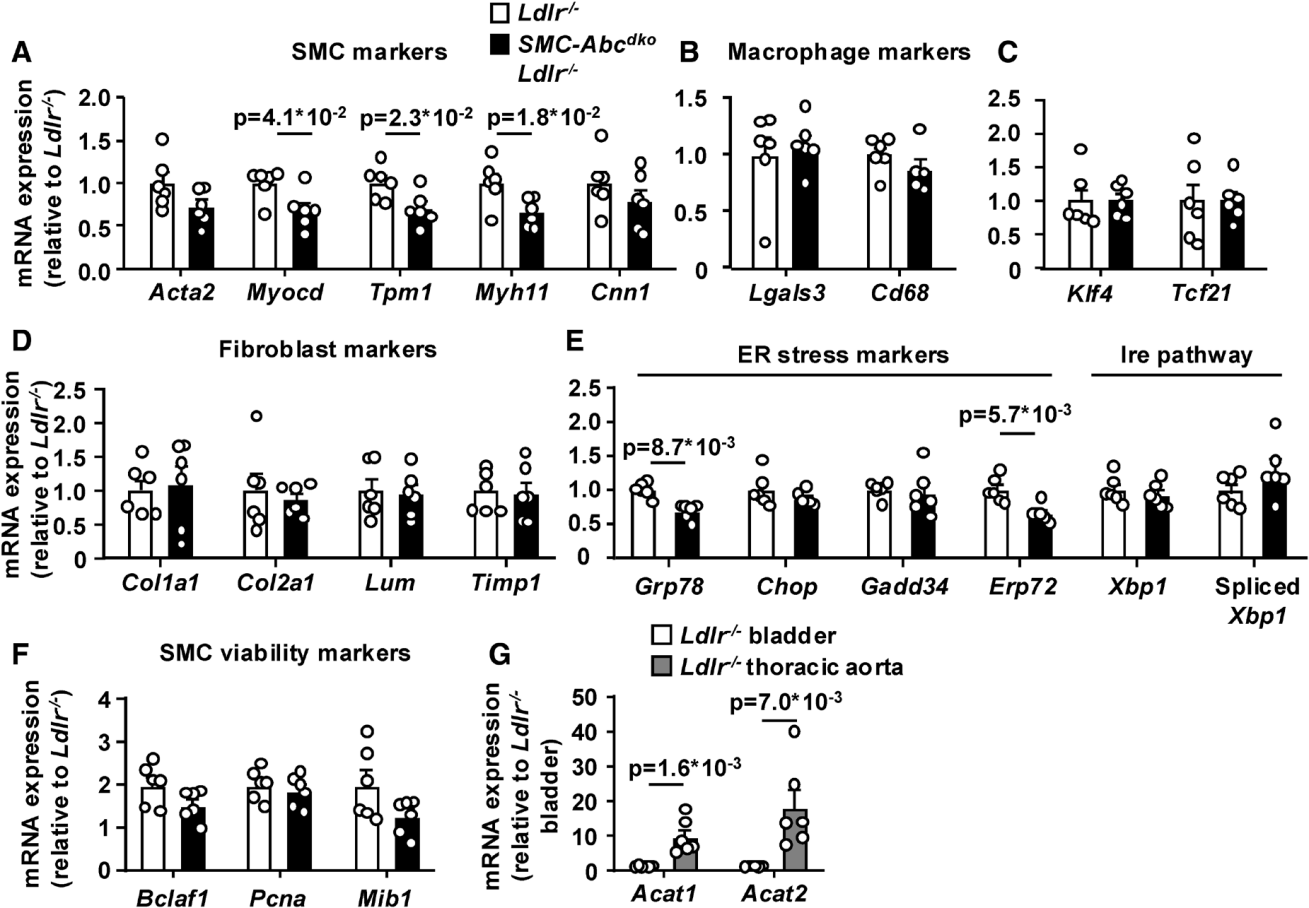
**Smooth muscle cell (SMC) *Abca1*/*Abcg1* deficiency does not induce differentiation into macrophage-like and fibroblast-like cells in the thoracic aorta in *Ldlr*^*−/−*^ mice fed western-type diet (WTD).** *Ldlr*^*−/−*^ and *SMC-Abc*^*dko*^*Ldlr*^*−/−*^ mice were fed WTD for 16 weeks. The thoracic aorta was isolated, digested, and mRNA expression of (**A**) SMC markers, (**B**) macrophage markers, (**C**) Krüppel-like factor 4 (*Klf4*) and transcription factor 21 (*Tcf21*), (**D**) fibroblast markers, (**E**) endoplasmic reticulum (ER) stress markers, as well as (**F**) *Bclaf-1* and associated cell viability markers was determined. **G**, The urinary bladder and thoracic aorta of *Ldlr*^*−/−*^ mice fed WTD for 6 and 16 weeks, respectively, were isolated, digested, and mRNA expression of *Acat1* and *Acat2* was determined (n=6 except for Cd68 (Cluster of differentiation 68) for *SMC-Abc*^*dko*^ *Ldlr*^*−/−*^, where n=5 due to 1 outlier being excluded based on the Grubbs test). Data are shown as mean±SEM. *P*<0.05 (2-way tailed unpaired *t* test except for *Myocd*, *Grp78*, and *ERP72*, which were calculated by Mann-Whitney *U* test) are indicated. Ire indicates inositol requiring enzyme.

Previous studies have shown that SMC to SEM cell transdifferentiation mediated by retinoic acid increases atherosclerosis and plaque vulnerability,^15^ as does SMC to macrophage transdifferentiation mediated by Klf4.^14,17^ ER stress in medial SMCs also enhances atherosclerosis.^27^ We thus examined the role of *SMC-Abca1/Abcg1* in transdifferentiation of aortic SMCs, using a similar approach to bladder SMCs. We fed *SMC-Abc*^*dko*^*Ldlr*^*−/−*^ mice and *Ldlr*^−/−^ controls a WTD for 16 weeks and isolated SMCs from the thoracic aorta. Using the thoracic aorta for these experiments allowed us to obtain a pure SMC population (Figure 1B; Figure S2). Furthermore, cholesterol loading of thoracic aorta SMCs has previously been shown to induce loss of SMC contractile markers and gain of macrophage markers.^24,49^ Although *SMC-Abca1/Abcg1* deficiency did induce loss of SMC contractile markers in thoracic aorta medial SMCs (Figure 6A), macrophage markers, *Klf4* and *Tcf21*, as well as fibroblast markers, ER stress markers, and SMC viability markers were not affected (Figure 6B through 6F). We then examined potential mechanisms for differences in SMC transdifferentiation between bladder and thoracic aorta SMCs. We had observed that *SMC-Abca1/Abcg1* deficiency induced free cholesterol, but not CE accumulation in bladder SMCs, while mainly inducing CE accumulation in thoracic aorta SMCs (Figures 1D and 4A). Previous studies have shown that inhibition of cholesterol esterification by ACAT (acyl-coenzyme A:cholesterol acyltransferase), concomitant with cyclodextrin-cholesterol loading induces SMC transdifferentiation to a larger extent than cyclodextrin-cholesterol loading alone.^49^ Cholesterol esterification via ACAT may thus protect against SMC transdifferentiation. We then examined *Acat1* and *Acat2* mRNA expression in thoracic aorta versus bladder SMCs. Although *Acat2* mRNA expression was 20-fold lower in aortic SMCs than *Acat1* mRNA expression (results not shown), both *Acat1* and *Acat2* mRNA expression were increased in thoracic aorta SMCs compared with bladder SMCs, by 10- and 18-fold, respectively (Figure 6G). This difference may account for cholesterol esterification in thoracic aorta, but not bladder SMCs. The high level of CE accumulation in thoracic aorta SMCs presumably explains why *SMC-Abca1/Abcg1* deficiency did not induce thoracic aorta SMC transdifferentiation, and also why we, after 16 weeks of WTD, found no atherosclerosis in the thoracic aorta, because no macrophages were present at this time point (Figure S2).

**Figure 7.**
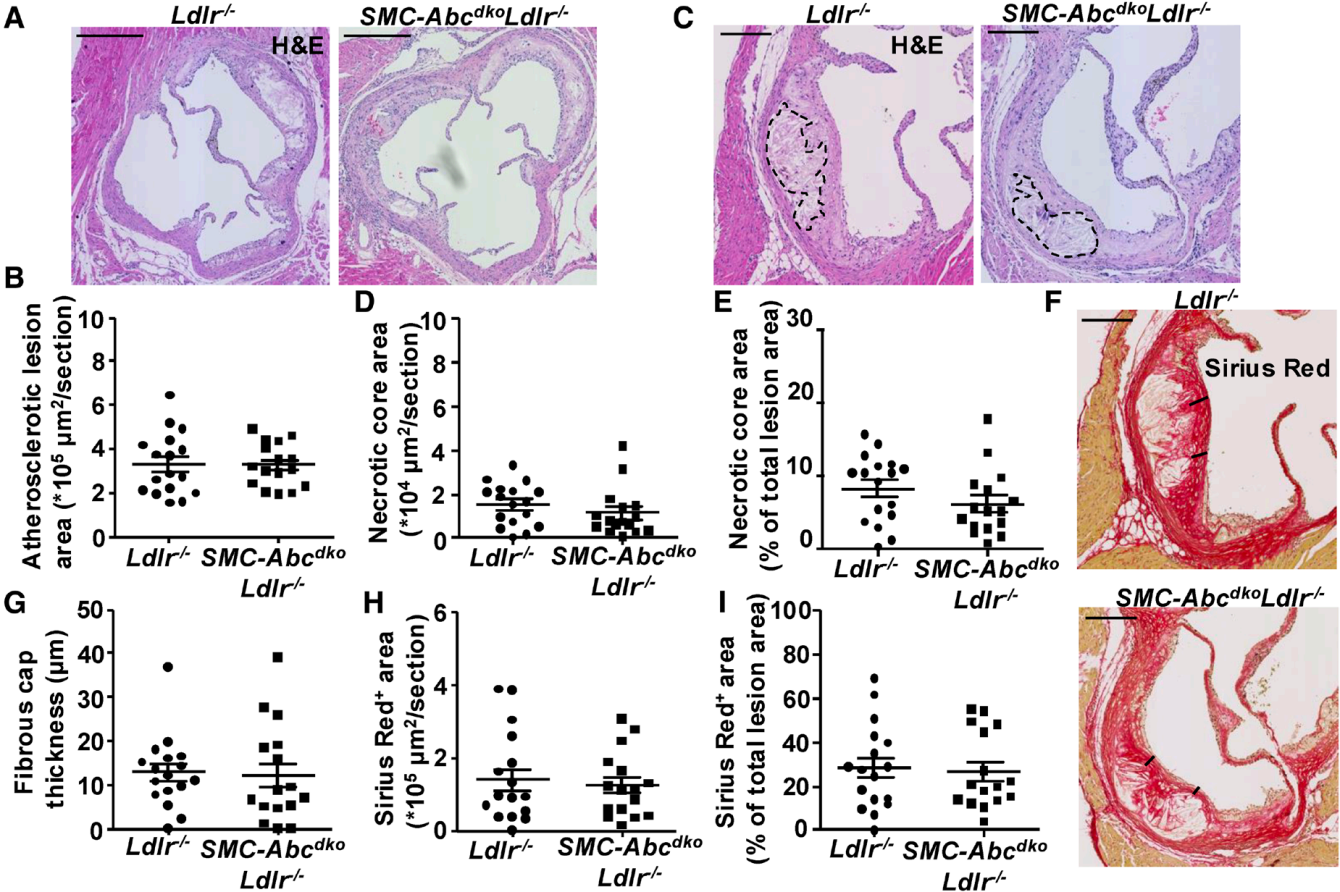
**Smooth muscle cell (SMC) *Abca1/Abcg1* deficiency does not affect atherosclerotic lesion area, necrotic core area, fibrous cap thickness, or collagen content in *Ldlr*^*−/−*^ mice fed western-type diet (WTD).** *Ldlr*^*−/−*^ and *SMC-Abc*^*dko*^*Ldlr*^*−/−*^ mice were fed WTD for 16 weeks. Hearts were isolated, sections were made of the aortic root and stained with hematoxylin and eosin (H&E). **A** and **B**, Representative example (**A**) and quantification (**B**) of atherosclerotic lesion area. Scale bar represents 400 µm. **C** through **E**, Representative example (**C**) and quantification of necrotic core area (**D**) and necrotic core area as % of total atherosclerotic lesion area (**E**). Black dashed lines indicate necrotic cores. **F** through **I**, Sections were stained with Sirius Red. **F**, Representative example of Sirius Red staining and quantification of fibrous cap thickness (**G**), collagen^+^ area (**H**), and collagen^+^ area as % of total atherosclerotic lesion area (**I**). Black bars indicate the fibrous cap (**A** through **I**; *Ldlr*^*−/−*^ (n=17), *SMC-Abc*^*dko*^*Ldlr*^*−/−*^ [n=16]). **C** and **F**, Scale bar represents 200 µm. **B** through **I**, Each data point represents an individual mouse. Data are shown as mean±SEM.

**Figure 8.**
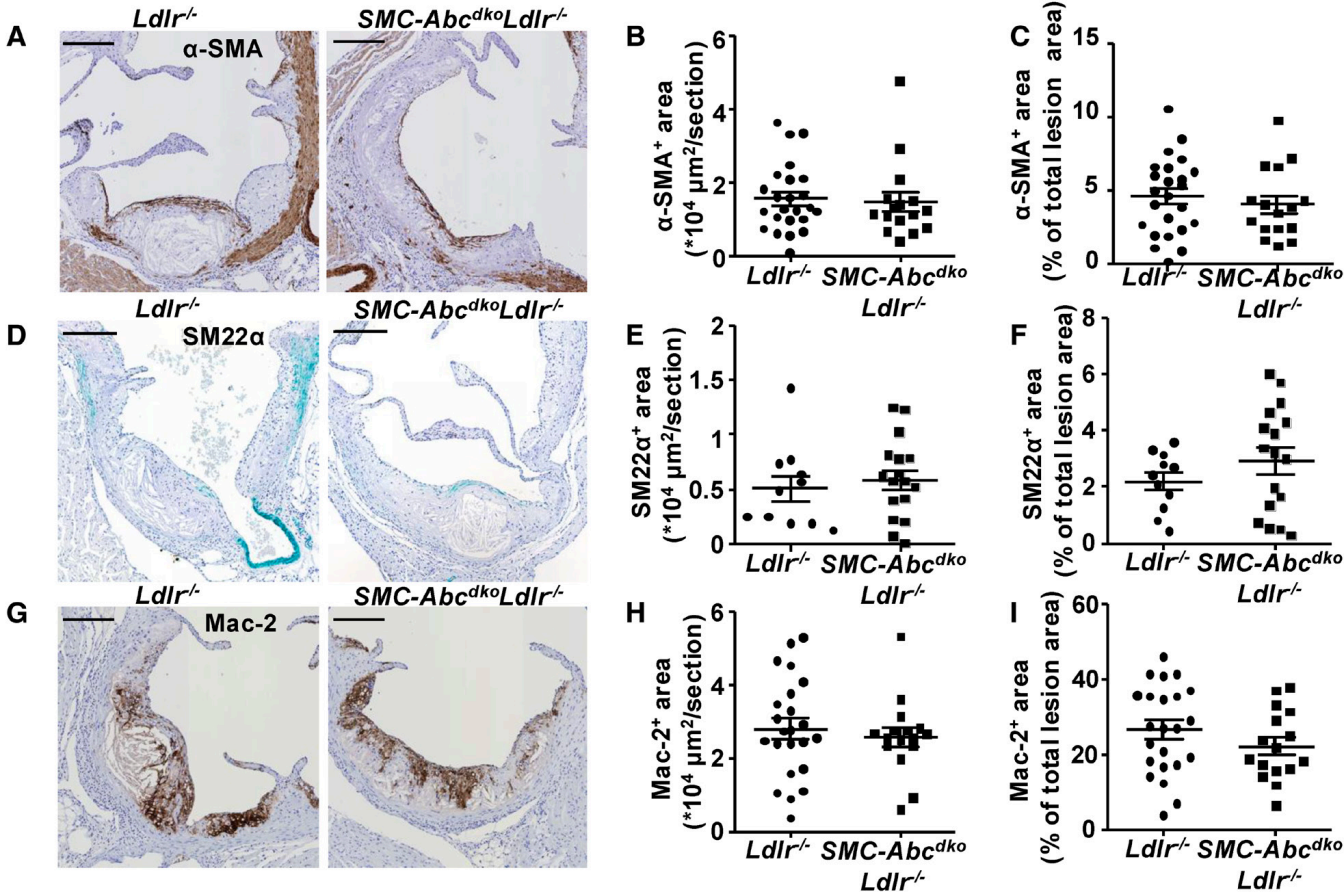
**Smooth muscle cell (SMC) *Abca1/Abcg1* deficiency does not affect α smooth muscle actin, SM22α (smooth muscle 22α), or Mac-2 (or Lgals3+; galectin-3+) area in *Ldlr*^*−/−*^ mice fed western-type diet (WTD).** *Ldlr*^*−/−*^ and *SMC-Abc*^*dko*^*Ldlr*^*−/−*^ mice were fed WTD for 16 weeks. Hearts were isolated, sections were made of the aortic root and stained for α-SMA (α-smooth muscle actin). **A**, Representative examples of α-SMA staining. **B** and **C**, Quantification of α-SMA^+^ area (**B**) and α-SMA^+^ area as % of total atherosclerotic lesion area (**C**). **A** through **C**, *Ldlr*^*−/−*^ (n=24), *SMC-Abc*^*dko*^*Ldlr*^*−/−*^ (n=16). **D** through **F**, Sections were stained for SM22α (Tagln). **D**, Representative examples of SM22α staining. **E** and **F**, Quantification of SM22α^+^ (Tagln^+^) area (**E**) and SM22α^+^ area as % of total atherosclerotic lesion area (**F**; *Ldlr*^*−/−*^ [n=11], *SMC-Abc*^*dko*^*Ldlr*^*−/−*^ [n=17]). **G** through **I**, Sections were stained for Mac-2 (Lgals3). **G**, Representative examples of Mac-2 staining. **H** and **I**, Quantification of Mac-2^+^ (Lgals3^+^) area (**H**) and Mac-2^+^ area as % of total atherosclerotic lesion area (**I**; *Ldlr*^*−/−*^ [n=23], *SMC-Abc*^*dko*^*Ldlr*^*−/−*^ [n=16]). **A**, **D**, and **G**, Scale bar represents 200 µm. **B** through **I**, Each data point represents an individual mouse. Data are shown as mean±SEM.

We then investigated the role of SMC *Abca1/Abcg1* in atherogenesis and plaque stability in the aortic root after 16 weeks of WTD feeding in *Ldlr*^*−/−*^ mice. Plasma cholesterol levels were not different between *SMC-Abca1*^*ko*^*Ldlr*^*−/−*^, *SMC-Abcg1*^*ko*^*Ldlr*^*−/−*^, or *SMC-Abc*^*dko*^*Ldlr*^*−/−*^ mice and their *Ldlr*^−/−^ controls (Table S4). Single *SMC-Abca1*, *SMC-Abcg1* (Figure S13A and S13B), or combined *SMC-Abca1/Abcg1* deficiency (Figure 7A and 7B) did not affect atherosclerotic lesion size in the aortic root of *Ldlr*^−/−^ mice fed WTD for 16 weeks. We then further characterized lesion composition and plaque stability. Plaque stability is reflected by smaller necrotic cores, increased fibrous cap thickness, and low ratio of macrophages compared with SMCs.^50^ Single *SMC-Abca1*, *SMC-Abcg1* (Figure S13C through S13F), or combined *SMC-Abca1/Abcg1* deficiency (Figure 7C through 7E) did not affect necrotic core area in atherosclerotic lesions of *Ldlr*^*−/−*^ mice fed WTD for 16 weeks. Sirius Red staining, representing collagen, of the aortic root showed that fibrous cap thickness was not affected by single *SMC-Abca1*, *SMC-Abcg1* (Figure S13G through S13H), or combined *SMC-Abca1/Abcg1* deficiency (Figure 7F through 7G) in atherosclerotic lesions of *Ldlr*^*−/−*^ mice fed WTD for 16 weeks. Similarly, the collagen content of atherosclerotic lesions was not affected by single *SMC-Abca1*, *SMC-Abcg1* (Figure S13I through S13L), or combined *SMC-Abca1/Abcg1* deficiency (Figure 7H and 7I). *SMC-Abca1/Abcg1* deficiency also did not affect the staining of the SMC markers α-SMA (Figure 8A through 8C; Figure S13M) and SM22α (smooth muscle 22α; Figure 8D through 8F; Figure S13N), the macrophage marker Mac-2 (Figure 8G through 8I), the chondrocyte marker Sox9 in the intima or media (Figure S13O through S13U), or the marker for modified SMCs or fibroblasts Lumican (Figure S13V through S13Y) or Oil Red O staining, reflecting lipid accumulation (Figure S14A and S14B) in atherosclerotic lesions of *Ldlr*^*−/−*^ mice after 16 weeks WTD. We verified that sections adjacent to those staining positive for Oil Red O stained positive for α-SMA (Figure S14C). Unlike in the thoracic aorta, in atherosclerotic plaques, *SMC-Abca1/Abcg1* deficiency did not affect lipid accumulation. Collectively, we found no effect of *SMC-Abca1/Abcg1* deficiency on atherosclerotic lesion size or plaque stability, at least with respect to necrotic core size or fibrous cap thickness, consistent with no effects on markers of SMCs, macrophages, chondrocytes or modified SMCs or fibroblasts in *Ldlr*^*−/−*^ mice fed WTD for 16 weeks. To further investigate whether SMCs would gain markers of macrophages in atherosclerotic plaques, we performed costainings for α-SMA and Mac-2 in atherosclerotic plaques. We found only minimal costaining between these 2 markers and no differences between the genotypes (Figure S15A through S15Q), suggestive of most Mac-2^+^ cells already having lost SMA expression. We then examined the atherosclerotic lesion area in the brachiocephalic artery (BCA) that shows a higher level of SMCs that gain markers of macrophages compared with the aortic root, according to previous studies.^14^ Similar to our findings in the aortic root, *SMC-Abca1/Abcg1* deficiency did not affect atherosclerotic lesion area, necrotic core area, Sirius red staining representing collagen, fibrous cap thickness, α-SMA staining or Mac-2 staining in the BCA (Figure S16A through S16O). Although we observed a higher percentage of α-SMA and Mac-2 costaining in the brachiocephalic artery than in the aortic root, this was not affected by *SMC-Abca1/Abcg1* deficiency (Figure S17A through S17L). Collectively, *SMC-Abca1/Abcg1* deficiency did not affect atherosclerosis or plaque composition in the aortic root or BCA.

## Discussion

Our findings show that during WTD-induced hypercholesterolemia, deficiency of Abca1- and Abcg1-mediated cholesterol efflux pathways in SMCs increases α_1_-AR–mediated vasoconstriction, α_1_-AR–dependent urinary bladder distension, and bladder SMC transdifferentiation into Lgals3^+^ SMCs, macrophage-like and fibroblast-like cells, accompanied by increased collagen deposition and inflammatory gene expression. Previous in vitro studies have suggested a link between cholesterol accumulation and α_1_-AR signaling^5,6^ and a role for SMC cholesterol accumulation in SMC transdifferentiation.^14,24^ We here show that defective SMC cholesterol efflux enhances α_1_-AR–mediated vasoconstriction and bladder SMC transdifferentiation in vivo, with especially the bladder SMC transdifferentiation contributing to urinary bladder distension. *SMC-Abca1/Abcg1* deficiency did not affect lesion size or composition in the aortic root or BCA in terms of fibrous cap thickness, necrotic core, or collagen content, consistent with *SMC-Abca1/Abcg1* deficiency not affecting α-SMA, SM22α, Mac-2, Sox9, Lumican, or costaining between α-SMA and Mac-2 in atherosclerotic plaques.

Our finding that *SMC-Abca1/Abcg1* deficiency induced free cholesterol accumulation in bladder SMCs versus mainly CE accumulation in medial aortic SMCs presumably explains why we observed bladder but not thoracic aorta SMC transdifferentiation. The mRNA expression of *Acat1* was 10-fold lower in the thoracic aorta compared with bladder SMCs, likely accounting for CE accumulation in aortic, but not bladder SMCs. Previous studies have shown that ACAT inhibition on top of cyclodextrin-cholesterol loading induces thoracic aorta SMC transdifferentiation to a greater extent than cyclodextrin-cholesterol loading alone,^49^ substantiating the key contribution of free cholesterol accumulation to this mechanism. Recent studies have shown that ER stress downstream of free cholesterol accumulation induces SMC transdifferentiation.^27^ Indeed, *SMC-Abca1/Abcg1* deficiency induced ER stress in bladder, but not thoracic aorta SMCs, consistent with increased free cholesterol accumulation and transdifferentiation of bladder SMCs. Of note, we had isolated thoracic aorta SMCs at the time point of 16 weeks of WTD feeding in *Ldlr*^*−/−*^ mice, when mice with *SMC-Abca1/Abcg1* had not developed atherosclerosis in the thoracic aorta, reflected by the Oil Red O positive area being restricted to SMCs of the thoracic aorta and the thoracic aorta being negative for Mac-2 staining. The lack of an effect of *SMC-Abca1/Abcg1* deficiency on atherosclerosis in the thoracic aorta may be due to thoracic aorta SMCs with *Abca1/Abcg1* deficiency not showing transdifferentiation or features of ER stress, a mechanism previously identified to increase SMC migration from the intima to the media, subsequent SMC transdifferentiation, and atherosclerosis.^27^ In addition, although cells in the media of the aortic root showed high expression of the chondrocyte marker Sox9, this was not affected by *SMC-Abca1/Abcg1* deficiency, suggesting that also in more athero-prone regions of the aorta, *SMC-Abca1/Abcg1* deficiency did not affect a marker of SMC transdifferentiation.

*SMC-Abca1/Abcg1* deficiency did not affect atherosclerotic lesion size or composition in the aortic root or BCA, consistent with markers of macrophages (Mac-2), chondrocytes (Sox9) or modified SMCs or fibroblasts (Lumican) or costaining between α-SMA and Mac-2 in atherosclerotic plaques not being affected. Several studies suggest that *Abca1/Abcg1* expression in plaque intimal SMCs may be low. Intimal SMCs in human atherosclerotic plaques from coronaries show low *ABCA1* mRNA expression compared with medial SMCs,^10^ a finding that has been replicated in plaques from mice.^26^ Low *ABCA1* mRNA expression has been suggested to contribute to SMCs gaining expression of the macrophage marker CD68, as shown by costainings of α-SMA and CD68 in this particular study.^11^ In addition, CD68^+^ cells in atherosclerotic plaques, of which ≈40% are from SMC origin,^14^ show low mRNA expression of *Abca1* and liver X receptor α (*Lxrα*),^51^ a transcription factor that regulates both *Abca1* and *Abcg1* expression,^7,52^ whereas macrophage *Abca1* and *Abcg1* expression in plaques are still high as suggested by single-cell RNA-sequencing studies. These studies showed high expression of *Abca1* and *Abcg1* in Trem2^hi^ macrophage foam cells of atherosclerotic plaques that comprise ≈19% of all macrophages in plaques.^45–47^ In addition, a recent single-cell RNA-sequencing study revealed low expression of *Abca1* in SMCs compared with SEM cells of mouse atherosclerotic plaques.^15^ Therefore, the lack of an effect of *SMC-Abca1/Abcg1* deficiency on atherosclerotic lesion size and composition may have been the consequence of low expression of these transporters in intimal SMCs in *Ldlr*^*−/−*^ mice fed WTD.

In addition, we found that *SMC-Abca1/Abcg1* deficiency increased α_1_-AR–mediated vasoconstriction in aortic rings of *Ldlr*^*−/−*^ mice fed WTD. This phenotype was most pronounced on combined *SMC-Abca1/Abcg1* deficiency, and present to a lesser extent in *SMC-Abca1*, but not *SMC-Abcg1* deficiency, consistent with SMCs showing high *Abca1* and low *Abcg1* expression in thoracic SMCs in our study.^10,29,53^ The increase in phenylephrine-induced vasoconstriction in WTD-fed *SMC-Abca1/Abcg1* deficient *Ldlr*^*−/−*^ and *Ldlr*^*−/−*^ mice was reversed by preincubation with MβCD, suggesting that phenylephrine-induced α_1_-AR–mediated effects were plasma membrane cholesterol-dependent, consistent with previous in vitro studies.^5,6^ In addition, we found that *SMC-Abca1/Abcg1* deficiency increased radioligand binding to the α_1_-AR. We used mechanistic studies using α_1a_-AR overexpression in HEK293T cells and found that augmenting Abca1- and Abcg1-mediated cholesterol efflux by rHDL^35^ suppressed α_1a_-AR surface expression and ERK phosphorylation downstream of the α_1a_-AR. Based on these studies, the increased radioligand binding to α_1_-AR on *SMC-Abca1/Abcg1* deficiency is most likely the consequence of increased α_1_-AR surface expression, in keeping with similar EC_50_ values for phenylephrine-induced vasoconstriction in *Ldlr*^*−/−*^ and *SMC-Abca1/Abcg1* deficient *Ldlr*^*−/−*^ mice. Despite increased α_1_-AR–mediated vasoconstriction, *SMC-Abca1/Abcg1* deficiency did not affect blood pressure in WTD-fed *Ldlr*^*−/−*^ mice, presumably because effects of endogenous α_1a_-AR or α_1d_-AR on blood pressure are relatively small (≈10%).^54,55^

We rather observed that the increase in α_1_-AR–mediated vasoconstriction on *SMC-Abca1/Abcg1* deficiency and the SMC dedifferentiation led to severe urinary bladder distension with collagen deposition in *Ldlr*^*−/−*^ mice fed WTD, a major cause for lower urinary tract symptoms (LUTS) in humans.^56^ Several epidemiological studies have shown that high plasma LDL cholesterol and low plasma HDL cholesterol levels are associated with LUTS, as is metabolic syndrome.^57,58^ Reduction of blood glucose levels and plasma LDL cholesterol by dietary intervention or statins decreases the incidence of LUTS,^56,59,60^ but underlying mechanisms are unknown. Macrophage cholesterol efflux may be decreased in metabolic syndrome, due to low plasma HDL, the acceptor for cholesterol efflux, or hyperglycemia, which decreases *Abca1* and *Abcg1* expression in monocytes and macrophages.^61,62^ Our data suggest that decreased SMC cholesterol efflux in metabolic syndrome could link adverse metabolic conditions, including diabetes and low HDL cholesterol, with urinary bladder distension, a major cause of LUTS.

In sum, we here show that SMC cholesterol efflux pathways control α_1_-AR–mediated vasoconstriction and SMC dedifferentiation, with implications for urinary bladder distension. Furthermore, we show that *SMC-Abca1/Abcg1* deficiency enhances bladder SMC transdifferentiation, presumably by increasing free cholesterol accumulation and ER stress. We attribute the lack of an effect of *SMC-Abca1/Abcg1* deficiency on atherosclerosis in the thoracic aorta to thoracic aorta SMCs not showing ER stress or transdifferentiation due mainly to CE accumulation and the lack of an effect of *SMC-Abca1/Abcg1* deficiency on atherosclerotic lesion size and composition to low expression of *Abca1/Abcg1* in intimal SMCs of *Ldlr*^*−/−*^ mice. It would be of interest to evaluate whether patients with metabolic syndrome or diabetes who show low expression of *Abca1/Abcg1* in monocytes due to hyperglycemia^61,62^ also show low *Abca1/Abcg1* expression in bladder SMCs. These SMCs could only be readily obtained in the context of a transplantation or bladder biopsy. Our findings may provide a mechanism for increased SMC dedifferentiation or vasoconstriction in these patients, with downstream effects on LUTS, and suggest that increasing SMC cholesterol efflux therapeutically may be beneficial in these conditions.

## Article Information

### Acknowledgments

The authors thank Jacques Debets for excellent technical assistance on immunostainings. The graphical abstract was prepared using BioRender.com.

### Author Contributions

B. Halmos and A.M. La Rose contributed to the conceptualization, methodology, investigation, and writing of the original draft. A.G. Groenen, D. Nakládal, and L.E. Deelman were responsible for designing the methodology, investigation, and writing—review and editing. V. Bazioti, D. Methorst, M.H. Koster, N.J. Kloosterhuis, A.v. Buiten, E.M. Schouten, N.C.A. Huijkman, M. Langelaar-Makkinje, L. Bongiovanni, S.M. De Neck, and A. de Bruin contributed to the investigation. H. Buikema, M.C. van den Heuvel, and J.C. Sluimer were involved in designing the methodology and investigation. F. Kuipers, I.J. de Jong, and R.H. Henning contributed to writing—review and editing. H.F. Jørgensen played a role in designing the methodology, investigation, funding acquisition, and writing—review and editing. M. Westerterp contributed to conceptualization, designing the methodology, investigation, funding acquisition, supervision, and writing—review and editing.

### Sources of Funding

This work was supported by Marie Skłodowska-Curie grant 945478 from European Union (EU) Horizon 2020, the Slovak Academic and Scientific Programme grant 3333/03/02, and the Slovak Research and Development Agency grant PP-MSCA-2022-0001 (support for projects Marie Skłodowska-Curie Actions-2022-0001) (to D. Nakládal); VIDI grant 917.15.350 and an Aspasia grant from the Netherlands Organization of Scientific Research and a Rosalind Franklin Fellowship with an EU Co-Fund attached from the University of Groningen (to M. Westerterp); and the British Heart Foundation—German Centre for Cardiovascular Research—Dutch Heart Foundation International Cardiovascular Research Partnership Award 02-001-2022-0125 (acronym PLAK TALK) to H.F. Jørgensen and M. Westerterp.

### Disclosures

None.

### Supplemental Material

Supplemental Methods

Tables S1–S4

Figures S1–17

Major Resources Table

References 63–72

## Supplementary Material


