# Supplementary material for: SMC Abca1 and Abcg1 Deficiency Enhances Urinary Bladder Distension but Not Atherosclerosis
Source: Circ Res. 2025 Feb 11;136(5):491–507. doi: 10.1161/CIRCRESAHA.124.325103 (PMC11867804; doi:10.1161/CIRCRESAHA.124.325103)
Supplement: Supplementary file 1 [file res-136-491-s001.pdf]

*** ARRIVE Reporting Guidelines**
This study involves use of animal models:
Yes

Manuscripts reporting on animal research are expected to adhere to the [ARRIVE] guidelines. A completed ARRIVE reporting guideline checklist will be required prior to acceptance and the information should be clearly presented in the manuscript. The following shortened version of the checklist should be completed at this time; a completed ARRIVE checklist is required to be uploaded during revision submission.

Animals
Species, age, sex, strain, and sources of animals are described:
Yes

Randomization
Randomization and allocation concealment were performed and described:
Yes

Blinding
Blinding was performed and the process described:
Yes

Inclusions and Exclusions (a)
Specific criteria for inclusions and exclusions are specified:
N/A

Inclusions and Exclusions (b)
Criteria for inclusions and exclusion were set before the study:
N/A

Reporting of Excluded Animals
All animals excluded after the randomization are reported:
N/A

Statistical Methods
Statistical Methods are described:
Yes

Date completed: 01/20/2025 13:04:01
User pid: 4910
