# Supplementary figures and images for: SMC Abca1 and Abcg1 Deficiency Enhances Urinary Bladder Distension but Not Atherosclerosis
Source: Circ Res. 2025 Feb 11;136(5):491–507. doi: 10.1161/CIRCRESAHA.124.325103 (PMC11867804; doi:10.1161/CIRCRESAHA.124.325103)

Uncut blots representative figures - Figure 2H

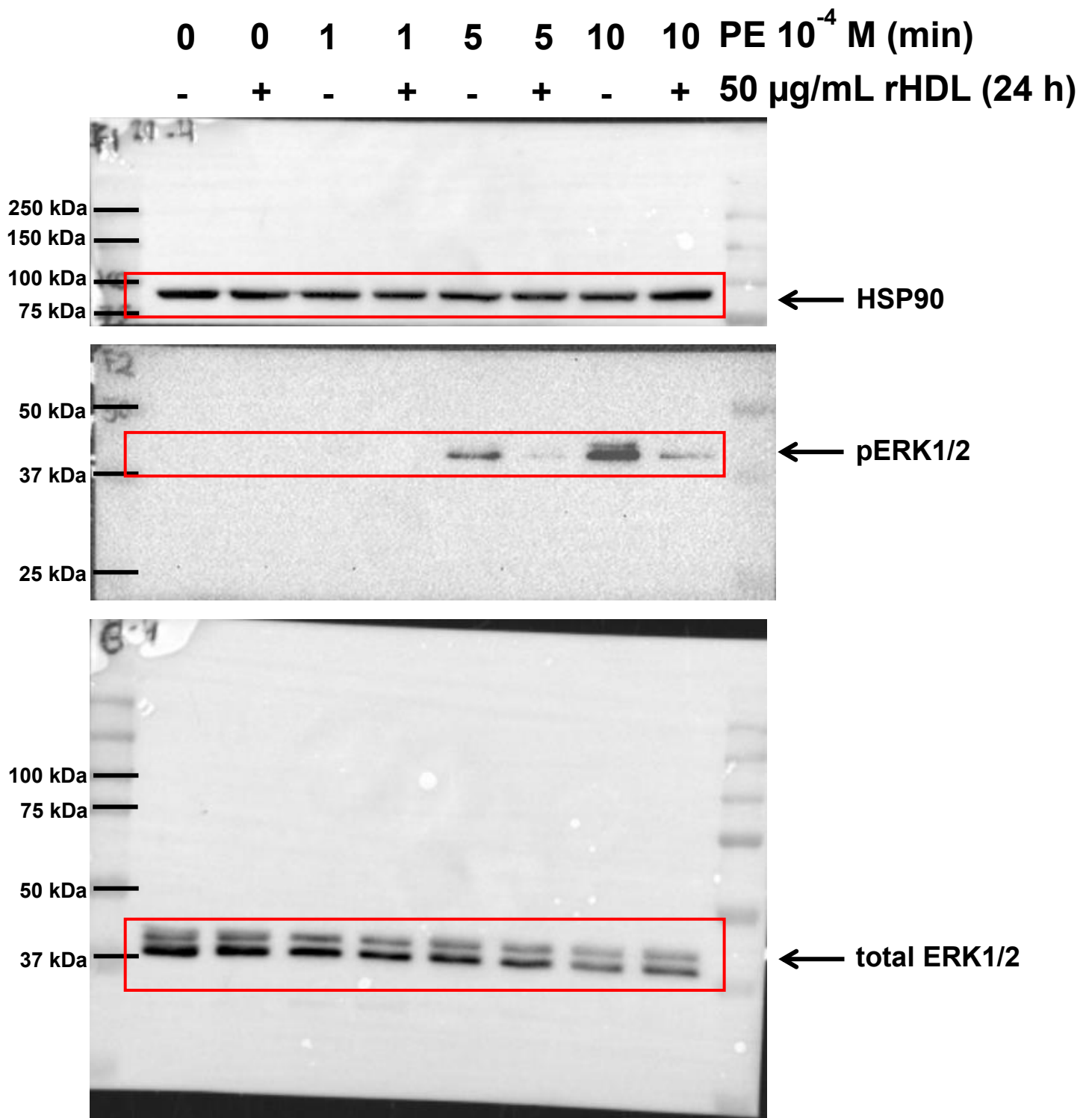

Figure 2H (continued)

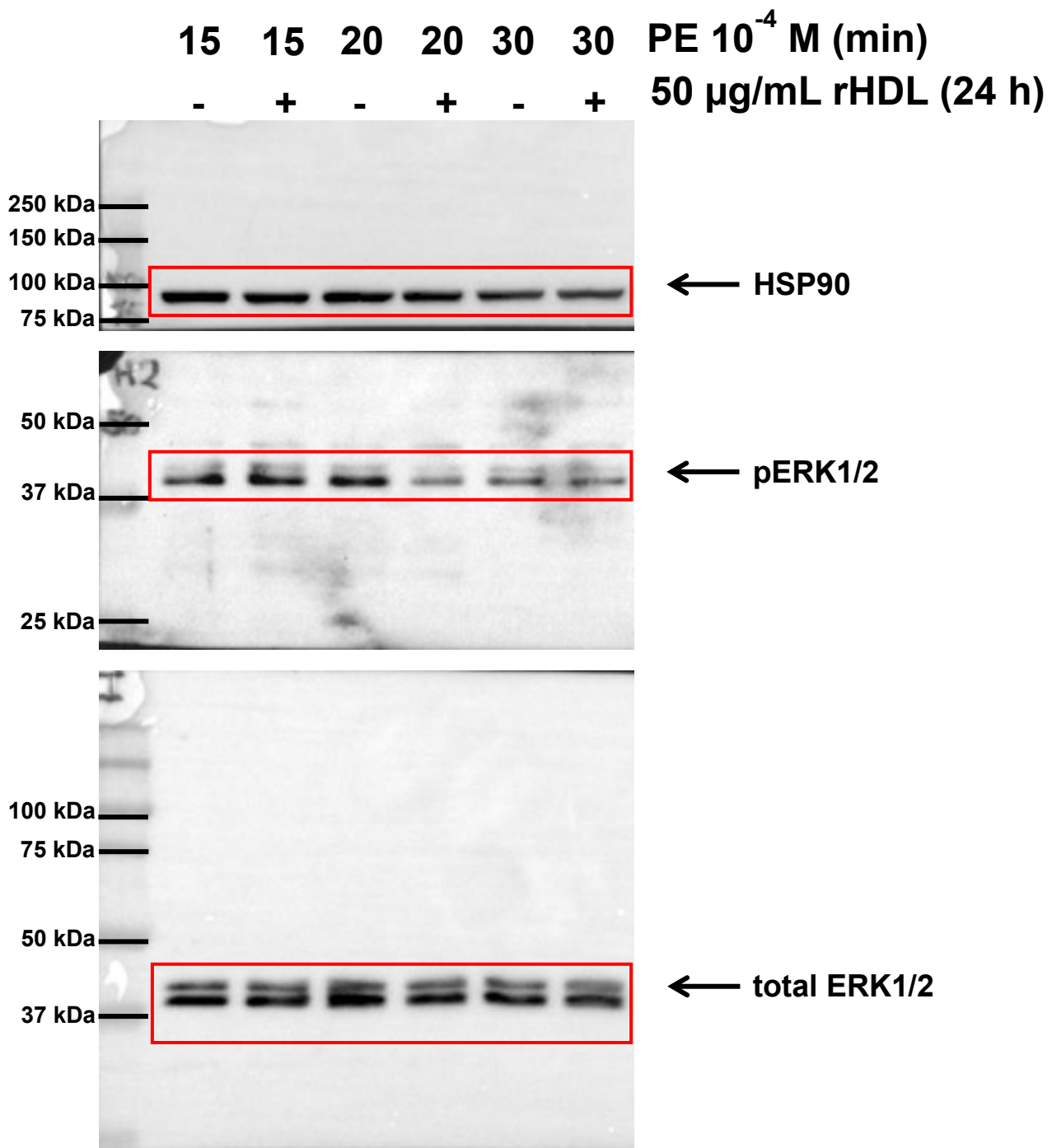

Supplement: Supplementary file 2 [file res-136-491-s002.pdf]
