# Supplementary material for: SMC Abca1 and Abcg1 Deficiency Enhances Urinary Bladder Distension but Not Atherosclerosis
Source: Circ Res. 2025 Feb 11;136(5):491–507. doi: 10.1161/CIRCRESAHA.124.325103 (PMC11867804; doi:10.1161/CIRCRESAHA.124.325103)
Supplement: Supplementary file 3 [file res-136-491-s003.pdf]

Major Resources Table

In order to allow validation and replication of experiments, all essential research materials listed in the Methods should be included in the Major Resources Table below. Authors are encouraged to use public repositories for protocols, data, code, and other materials and provide persistent identifiers and/or links to repositories when available. Authors may add or delete rows as needed.

Animals (in vivo studies)

Species	Vendor or Source	Background Strain	Sex	Persistent ID / URL
Mouse: B6.Cg-$Abca1^{tm1Jp}$ $Abcg1^{tm1Tall}$/J	[63], deposited at The Jackson Laboratory	C57BL/6	Male	RRID:IMSR_JAX:021067
Mouse: $Myh11Cre^{ERT2}$B6.FVB-Tg(myh11-cre/ERT2)1Soff/J	The Jackson Laboratory	C57BL/6	Male	RRID:IMSR_JAX:019079
Mouse: $Ldlr^{-/-}$: B6.129S7-$Ldlr^{tm1Her}$/J	The Jackson Laboratory	C57BL/6	Male	RRID:IMSR_JAX:002207
Mouse: $Myh11Cre^{ERT2}Myocd^{fl/fl}$	Dr. Eric Olson, UT Southwestern (gift)	C57BL/6	Male	

Genetically Modified Animals

	Species	Vendor or Source	Background Strain	Other Information	Persistent ID / URL
**Male**	$Myh11Cre^{ERT2}Abca1^{fl/fl}Abcg1fl/flLdlr^{-/-}$	This paper	C57BL/6		This paper
**Male**	$Myh11Cre^{ERT2}Abca1^{fl/fl}Abcg1^{+/+}Ldlr^{-/-}$	This paper	C57BL/6		This paper
**Male**	$Myh11Cre^{ERT2}Abca1^{+/+}Abcg1^{fl/fl}Ldlr^{-/-}$	This paper	C57BL/6		This paper
**Male**	$Myh11Cre^{ERT2}Abca1^{+/+}Abcg1^{+/+}Ldlr^{-/-}$	This paper	C57BL/6		This paper
**Male**	$Abca1^{fl/fl}Abcg1^{fl/fl}Ldlr^{-/-}$	This paper	C57BL/6		This paper
**Male**	$Myh11Cre^{ERT2}Myocd^{fl/fl}$	This paper	C57BL/6		This paper

Antibodies

Target antigen	Vendor or Source	Catalog #	Working concentration	Lot # (preferred but not required)	Persistent ID / URL
Rabbit anti Smooth Muscle Actin	Lab Vision	RB9010P1	1/200		RRID: AB_149756
Biotinylated goat anti-rabbit	Vector Laboratories	BA-1000	1/250		RRID:AB_2313606
Rat Anti-Mouse/Human Mac-2 (Lgals3)	Cedarlane	CL8942AP	1/10.000		RRID:AB_2534074; clone M3/38
Biotinylated goat anti-rat	Vector Laboratories	BA-9400	1/125		RRID:AB_2336202
Anti-actin α-smooth muscle Cy3 antibody	Sigma-Aldrich	C6198	1/500		RRID:AB_476856
Goat anti-rat IgG (H+L) AF647	ThermoFisher	A-21247	1/100		RRID:AB_141778

DOI [to be added]

Goat anti-rat IgG (H+L) AF488	ThermoFisher	A-11006	1/200		RRID:AB_10060357
Anti-FLAG M2	Sigma Aldrich	F1804	1/200		RRID:AB_262044
Goat anti-mouse IgG (H+L) APC	ThermoFisher	A-865	1/200		RRID:AB_2536211
Rabbit HSP90	Cell Signaling Technology	4874	1/1.000		RRID:AB_2121214
Rabbit p44/42 MAPK (Erk1/2)	Cell Signaling Technology	4695	1/1.000		RRID:AB_390779
Rabbit Phosphop44/42 MAPK (pErk1/2)	Cell Signaling Technology	4370	1/1.000		RRID:AB_2315112
Goat anti-rabbit HRP conjugated	Bio-rad	1706515	1/10.000		RRID:AB_11125142
Rabbit anti-mouse Sox9	Abcam	ab185230	1/2.0000		RRID: AB_2715497
Goat anti-mouse/human Tagln/SM22α	Abcam	ab10135	1/400		RRID:AB_225631
Donkey anti-goat HRP conjugated	ThermoFisher	A16005	1/500		RRID:AB_2534679
Rabbit anti-Lumican	Abcam	Ab168348	1/5.000		RRID:AB_2920864
Brightvision poly HRP anti-rabbit IgG	VWR	DPVR55HRP	Ready to use concentration		RRID:AB_2915958

qPCR primers

Name	Sequence	Source / Repository	Amplicon size/T_m
Mouse *Abca1*$^{fl/fl}$	Fw: 5'-GTGAATGGGCAATTCGCAAACT-3'<br>Rv: 5'-AGATCTCCCCTCCTTGACAATGC-3'	Previous publication[42]	215 bp / 60˚C
Mouse *Abcg1*$^{fl/fl}$	Fw: 5'-TGTTCAGGAGGCCATGATGGT-3'<br>Rv: 5'-TGGCCAGGCGTTTCCG-3'	Previous publication[42]	161 bp / 60˚C
Mouse *Acta2*	Fw: 5'-GCTTCGCTGGTGATGATGCTC-3'<br>Rv: 5'-AGTTGGTGATGATGCCGTGTTC-3'	This paper	177 bp / 60˚C
Mouse *Myocd*	Fw: 5'-AAGGTCCATTCCAACTGCTC-3'<br>Rv: 5'-CCATCTCTACTGCTGTCATCC-3'	This paper	216 bp / 56˚C
Mouse *Tpm1*	Fw: 5'-AGCTCGACAAAGAGAACGCC-3'<br>Rv: 5'-ATCTTCCAGCTGCTTGCTCC-3'	This paper	89 bp / 59˚C
Mouse *Myh11*	Fw: 5'-TCAACGCCAACCGCAGGAAGCTG-3'<br>Rv: 5'-TGCTAAGCAGTCTGCTGGGCT-3'	This paper	305 bp / 64˚C
Mouse *Cnn1*	Fw: 5'-GGACCAGGCGACCATCAG-3'<br>Rv: 5'-TAGGCAGAGTTGTAGTAGTTGTG-3'	This paper	297 bp / 58˚C
Mouse *Ly6a*	Fw: 5'-AGGAGGCAGCAGTTATTGTGG-3'<br>Rv: 5'-CGTTGACCTTAGTACCCAGGA-3'	This paper	114 bp / 59˚C
Mouse *Vcam-1*	Fw: 5'-AGTTGGGGATTCGGTTGTTCT-3'<br>Rv: 5'-CCCCTCATTCCTTACCACCC-3'	This paper	112 bp / 58˚C
Mouse *Cd68*	Fw: 5'-CTTCCCACAGGCAGCACAG-3'<br>Rv: 5'-ATGATGAGAGGCAGCAAGAGG-3'	This paper	234 bp / 59˚C

DOI [to be added]

Mouse *Lgals3*	Fw: 5'-AGGAGAGGGAATGATGTTGCC-3'<br>Rv: 5'-GGTTTGCCACTCTCAAAGGG-3'	This paper	143 bp / 58˚C
Mouse *Col1a1*	Fw: 5'-GCTCCTCTTAGGGGCCACT-3'<br>Rv: 5'-CCACGTCTCACCATTGGGG-3'	This paper	103 bp / 59˚C
Mouse *Col1a2*	Fw: 5'-GTAACTTCGTGCCTAGCAACA-3'<br>Rv: 5'-CCTTTGTCAGAATACTGAGCAGC-3'	This paper	230 bp / 56˚C
Mouse *Col2a1*	Fw: 5'-GGGAATGTCCTCTGCGATGAC-3'<br>Rv: 5'-GAAGGGGATCTCGGGGTTG-3'	This paper	66 bp / 59˚C
Mouse *Mmp2*	Fw: 5'-CAAGTTCCCCGGCGATGTC-3'<br>Rv: 5'-TTCTGGTCAAGGTCACCTGTC-3'	This paper	171 bp / 59˚C
Mouse *Mmp3*	Fw: 5'-ACCTATTCCTGGTTGCTG-3'<br>Rv: 5'-GCCTTGGCTGAGTGGTAG-3'	This paper	64 bp / 52˚C
Mouse *Lum*	Fw: 5'-CTCTTGCCTTGGCATTAGTCG-3'<br>Rv: 5'-GGGGGCAGTTACATTCTGGTG-3'	This paper	114 bp / 58˚C
Mouse *Timp1*	Fw: 5'-GCAACTCGGACCTGGTCATAA-3'<br>Rv: 5'-CGGCCCGTGATGAGAAACT-3'	This paper	226 bp / 58˚C
Mouse *Chad*	Fw: 5'-GTCATCTGCGACAAGGTGGG-3'<br>Rv: 5'-CATGGTCCGAAACGAGTTGG-3'	This paper	117 bp / 58˚C
Mouse *Sox9*	Fw: 5'-CACCCCGATTACAAGTACCAG-3'<br>Rv: 5'-TGCTCAGTTCACCGATGTCCA-3'	This paper	334 bp / 58˚C
Mouse *Klf4*	Fw: 5'-CTTTCCTGCCAGACCAGATG-3'<br>Rv: 5'-GGTTTCTCGCCTGTGTGAGT-3'	This paper	226 bp / 56˚C
Mouse *Tcf21*	Fw: 5'-CCCACTAAGAAAAGCCCGCTC-3'<br>Rv: 5'-CCGTTCTCGTACTTGTCGTTG-3'	This paper	242 bp / 58˚C
Mouse *Trem2*	Fw: 5'-CTGGAACCGTCACCATCACTC-3'<br>Rv: 5'-CGAAACTCGATGACTCCTCGG-3'	This paper	183 bp / 59˚C
Mouse *Spp1*	Fw: 5'-AGCAAGAAACTCTTCCAAGCAA-3'<br>Rv: 5'-GTGAGATTCGTCAGATTCATCCG-3'	This paper	134 bp / 56˚C
Mouse *Cd11c*	Fw: 5'-CTGGATAGCCTTTCTTCTGCTG-3'<br>Rv: 5'-GCACACTGTGTCCGAACTC-3'	This paper	113 bp / 57˚C
Mouse *Cd9*	Fw: 5'-ATGCCGGTCAAAGGAGGTAG-3'<br>Rv: 5'-GCCATAGTCCAATAGCAAGCA-3'	This paper	103 bp / 57˚C
Mouse *Mertk*	Fw: 5'-CAGGGCCTTTACCAGGGAGA-3'<br>Rv: 5'-TGTGTGCTGGATGTGATCTTC-3'	This paper	111 bp / 58˚C
Mouse *Lxra*	Fw: 5'-TGGTAATGTCCAGGGCTCCAG-3'<br>Rv: 5'-TCCACAACTCCGTTGCAGAA-3'	This paper	91 bp / 59˚C
Mouse *Lipa*	Fw: 5'-TGTTCGTTTTCACCATTGGGA-3'<br>Rv: 5'-CGCATGATTATCTCGGTCACA-3'	This paper	103 bp / 56˚C
Mouse *Npc1*	Fw: 5'-TGTTTGGTATGGAGAGTGTGGA-3'<br>Rv: 5'-GTCACAGCAGAGACTGACATTG-3'	This paper	154 bp / 57˚C
Mouse *Ctsb*	Fw: 5'-TCCTTGATCCTTCTTTCTTGCC-3'<br>Rv: 5'-ACAGTGCCACACAGCTTCTTC-3'	This paper	176 bp / 57˚C
Mouse *Ctsl*	Fw: 5'-ATCAAACCTTTAGTGCAGAGTGG-3'<br>Rv: 5'-CTGTATTCCCCGTTGTGTAGC-3'	This paper	136 bp / 57˚C
Mouse *Cd36*	Fw: 5'-GATCGGAACTGTGGGCTCAT-3'<br>Rv: 5'-GGTTCCTTCTTCAAGGACAACTTC-3'	This paper	135 bp / 56˚C
Mouse *Msr1*	Fw: 5'-GCACAATCTGTGATGATCGCT-3'<br>Rv: 5'-CCCAGCATCTTCTGAATGTGAA-3'	This paper	224 bp / 57˚C
Mouse *Cd163*	Fw: 5'-ATGGGTGGACACAGAATGGTT-3'<br>Rv: 5'-CAGGAGCGTTAGTGACAGCAG-3'	This paper	130 bp / 59˚C

DOI [to be added]

Mouse *Fabp2*	Fw: 5’-GTGGAAAGTAGACCGGAACGA-3’ Rv: 5’-CCATCCTGTGTGATTGTCAGTT-3’	This paper	117 bp / 57˚C
Mouse *Fabp4*	Fw: 5’-CACCATCCGGTCAGAGAGTACTT-3’ Rv: 5’-TCTAGGGTTATGATGCTCTTCACCT-3’	This paper	114 bp / 57˚C
Mouse *Fabp5*	Fw: 5’-TGAAAGAGCTAGGAGTAGGACTG-3’ Rv: 5’-CTCTCGGTTTTGACCGTGATG-3’	This paper	106 bp / 57˚C
Mouse *Mmp12*	Fw: 5’-TGAATTTGCATTTCCTGTACATAGT-3’ Rv: 5’-TGCTGTAAGTCCATGGGTGA-3’	This paper	61 bp / 56˚C
Mouse *Mmp14*	Fw: 5’-GGATACCCAATGCCCATTGGCCA-3’ Rv: 5’-CCATTGGGCATCCAGAAGAGAGC-3’	This paper	221 bp / 62˚C
Mouse *Il-1β*	Fw: 5’-TGCAGCTGGAGAGTGTGG-3’ Rv: 5’-TGCTTGTGAGGTGCTGATG-3’	This paper	143 bp / 57˚C
Mouse *Nlrp3*	Fw: 5’-ATTACCCGCCCGAGAAAGG-3’ Rv: 5’-TCGCAGCAAAGATCCACACAG-3’	This paper	141 bp / 58˚C
Mouse *Il-6*	Fw: 5’-CTGCAAGAGACTTCCATCCAGTT-3’ Rv: 5’-AGGGAAGGCCGTGGTTGT-3’	This paper	65 bp / 59˚C
Mouse *Ifnar1*	Fw: 5’-AGCCACGGAGAGTCAATGG-3’ Rv: 5’-GCTCTGACACGAAACTGTGTTTT-3’	This paper	167 bp / 56˚C
Mouse *Tnfα*	Fw: 5’-GTAGCCCACGTCGTAGCAAAC-3’ Rv: 5’-AGTTGGTTGTCTTTGAGATCCATG-3’	This paper	107 bp / 57˚C
Mouse *Mcp-1*	Fw: 5’-GCTGGAGAGCTACAAGAGGATCA-3’ Rv: 5’-ACAGACCTCTCTCTTGAGCTTGGT-3’	This paper	85 bp / 60˚C
Mouse *Ccr2*	Fw: 5’-ATCCACGGCATACTATCAACATC-3’ Rv: 5’-CAAGGCTCACCATCATCGTAG-3’	This paper	104 bp / 56˚C
Mouse *Ccl3*	Fw: 5’-TTCTCTGTACCATGACACTCTGC-3’ Rv: 5’-CGTGGAATCTTCCGGCTGTAG-3’	This paper	100 bp / 58˚C
Mouse *Cxcl1*	Fw: 5’-CTGGGATTCACCTCAAGAACATC-3’ Rv: 5’-CAGGGTCAAGGCAAGCCTC-3’	This paper	117 bp / 58˚C
Mouse *Cxcl2*	Fw: 5’-CCAACCACCAGGCTACAGG-3’ Rv: 5’-GCGTCACACTCAAGCTCTG-3’	This paper	108 bp / 58˚C
Mouse *Gpr183*	Fw: 5’-ATGGCTAACAATTTCACTACCCC-3’ Rv: 5’-ACCAGCCCAATGATGAAGACC-3’	This paper	128 bp / 58˚C
Mouse *Tgfbr2*	Fw: 5’-CCGCTGCATATCGTCCTGTG-3’ Rv: 5’-AGTGGATGGATGGTCCTATTACA-3’	This paper	131 bp / 57˚C
Mouse *Arg1*	Fw: 5’-CTCCAAGCCAAAGTCCTTAGAG-3’ Rv: 5’-AGGAGCTGTCATTAGGGACATC-3’	This paper	185 bp / 57˚C
Mouse *Irf7*	Fw: 5’-GAGACTGGCTATTGGGGGAG-3’ Rv: 5’-GACCGAAATGCTTCCAGGG-3’	This paper	102 bp / 58˚C
Mouse *Isg15*	Fw: 5’-GGTGTCCGTGACTAACTCCAT-3’ Rv: 5’-TGGAAAGGGTAAGACCGTCCT-3’	This paper	131 bp / 59˚C
Mouse *Cd115*	Fw: 5’-TGTCATCGAGCCTAGTGGC-3’ Rv: 5’-CGGGAGATTCAGGGTCCAAG-3’	This paper	134 bp / 57˚C
Mouse *Lyve1*	Fw: 5’-CAGCACACTAGCCTGGTGTTA-3’ Rv: 5’-CGCCCATGATTCTGCATGTAGA-3’	This paper	112 bp / 58˚C
Mouse *Acat1*	Fw: 5’-CAGGAAGTAAGATGCCTGGAAC-3’ Rv : 5’-TTCACCCCCTTGGATGACATT-3’	This paper	228 bp / 58˚C
Mouse *Acat2*	Fw: 5’-AGTCCATAGCCATGGGCGACTCCACCATTG-3’ Rv: 5’-CACCCATCCTGACTCCTGTTCTCAGGTGAG-3’	This paper	105 bp / 67˚C
Mouse *Grp78*	Fw: 5’-TGGTGATCAGGATACAGGTGATCT-3’ Rv: 5’-GGTACCACAGTGTTCCTTGGAA-3’	This paper	114 bp / 59˚C

DOI [to be added]

Mouse *Chop*	Fw: 5’-CTGGAAGCCTGGTATGAGGAT-3’<br>Rv: 5’-CAGGGTCAAGAGTAGTGAAGGT-3’	This paper	121 bp /<br>56˚C
Mouse *Gadd34*	Fw: 5’-AGAGGCGGCTCAGATTGTTC-3’<br>Rv: 5’-CGAAGTGTACCTTCCGAGCTTT-3’	This paper	81 bp /<br>58˚C
Mouse *Erp72*	Fw: 5’-ACGCCACCGAACAGACAGAC-3’<br>Rv: 5’-CCAGACTGCTCAATCATGTAG-3’	This paper	145 bp /<br>57˚C
Mouse *Xbp1*	Fw: 5’-GACAGAGAGTCAAACTAACGTGG-3’<br>Rv: 5’-GTCCAGCAGGCAAGAAGGT-3’	This paper	190 bp /<br>57˚C
Mouse spliced<br>*Xbp1*	Fw: 5’-AAGAACACGCTTGGGAATGG-3’<br>Rv: 5’-CTGCACCTGCTGCGGAC-3’	This paper	113 bp /<br>58˚C
Mouse *Bclaf1*	Fw: 5’-ACTCACCACGAGATGAAAGACT-3’<br>Rv: 5’-TGTCCCAGCAAAAACTCCTCT-3’	This paper	111 bp /<br>58˚C
Mouse *Pcna*	Fw: 5’-TTTGAGGCACGCCTGATCC-3’<br>Rv: 5’-GGAGACGTGAGACGAGTCCAT-3’	This paper	135 bp /<br>58˚C
Mouse *Mib1*	Fw: 5’-AGTTGGCCGAGTACAACAGAT-3’<br>Rv: 5’-TGTTCCACAGACTTCCACCTT-3’	This paper	61 bp /<br>58˚C
Mouse *Cd14*	Fw: 5’-AACCTGGAAGCCAGAGAACAC-3’<br>Rv: 5’-ACACGCTCCATGGTCGGTAG-3’	This paper	109 bp /<br>59˚C
Mouse *Alox5*	Fw: 5’-ACTACATCTACCTCAGCCTCATT-3’<br>Rv: 5’-GGTGACATCGTAGGAGTCCAC-3’	This paper	113 bp /<br>58˚C
Mouse *Tlr2*	Fw: 5’-TCAGACAAAGCGTCAAATCTC-3’<br>Rv: 5’-GCCACCAAGATCCAGAAGAG-3’	This paper	64 bp /<br>55˚C
Mouse *Gapdh*	Fw: 5’-ATTGTCAGCAATGCATCCTG-3’<br>Rv: 5’-ATGGACTGTGGTCATGAGCC-3’	This paper	102 bp /<br>57˚C
Mouse *36B4*	Fw: 5’-GGACCCGAGAAGACCTCCTT-3’<br>Rv: 5’-GCACATCACTCAGAATTTCAATGG-3’	This paper	85 bp /<br>56˚C
Human *ACTA2*	Fw: 5’-AAAAGACAGCTACGTGGGTGA-3’<br>Rv: 5’-GCCATGTTCTATCGGGTACTTC-3’	This paper	76 bp /<br>57˚C
Human<br>*MYOCARDIN*	Fw: 5’-ACGGATGCTTTTGCCTTTGAA-3’<br>Rv: 5’-AACCTGTCGAAGGGGTATCTG-3’	This paper	133 bp /<br>57˚C
Human *LGALS3*	Fw: 5’-ATGGCAGACAATTTTTCGCTCC-3’<br>Rv: 5’-GCCTGTCCAGGATAAGCCC-3’	This paper	173 bp /<br>58˚C
Human *CD68*	Fw: 5’-GCTACATGGCGGTGGAGTACAA-3’<br>Rv: 5’-ATGATGAGAGGCAGCAAGATGG-3’	This paper	262 bp /<br>60˚C
Human *KLF4*	Fw: 5’-CGGACATCAACGACGTGAG-3’<br>Rv: 5’-GACGCCTTCAGCACGAACT-3’	This paper	139 bp /<br>58˚C
Human *CYCLOA*<br>*(PPIA)*	Fw: 5’-ATGTGTCAGGGTGGTGACTTC-3’<br>Rv: 5’-GCCATCCAACCACTCAGTCT-3’	This paper	192 bp /<br>58˚C
Human *B-ACTIN*	Fw: 5’-CATGTACGTTGCTATCCAGGC-3’<br>Rv: 5’-CTCCTTAATGTCACGCACGAT-3’	This paper	250 bp /<br>57˚C

Cultured Cells

Name	Vendor or Source	Sex (F, M, or unknown)	Persistent ID / URL
Bladder SMCs	Lonza	Unknown	Cat# CC-2533
HEK293T Cells	ATCC	Female	Cat# CRL-3216
HEK293A Cells	Invitrogen	Unknown	Cat# R705-07

Data & Code Availability

Description	Source / Repository	Persistent ID / URL

DOI [to be added]

N/A		

Other

Description	Source / Repository	Persistent ID / URL
Oil Red O	Sigma-Aldrich	Cat# O0625
U46619	Sigma-Aldrich	Cat# D8174
Acetylcholine	Sigma-Aldrich	Cat# A6625
Sodium Nitroprusside	Sigma-Aldrich	Cat# 71778
Phenylephrine	Sigma-Aldrich	Cat# P6126
Serotonin	Sigma-Aldrich	Cat# H7752
Potassium chloride	Merck	Cat# 104936
Methyl-β-cyclodextrin	Sigma-Aldrich	Cat# C4555
Cyclodextrin-cholesterol	Sigma-Aldrich	Cat# C4951
Liberase TH	Sigma-Aldrich	Cat# 5401135001
DNAse I	Sigma-Aldrich	Cat# DN25
Hyalurodinase	Sigma-Aldrich	Cat# H3506
Elastase type III	Sigma-Aldrich	Cat# E0127
Collagenase type II	Worthington Biochemical	Cat# LS004176
[7-methoxy-^{3}H]-prazosin	PerkinElmer	Cat# NET823250UC
Tamsulosin hydrochloride	Sigma-Aldrich	Cat# T1330
Sirius Red	Sigma-Aldrich	Cat# 365548
Vectastain ABC-peroxidase kit	Vector Laboratories	Cat#PK-4000
Cholesterol enzymatic kit	Diasys Diagnostic Systems	Cat# 113009910026
Cholesterol Standard FS	Diasys Diagnostic Systems	Cat# 113009910030
Creatinine plus VER.2 assay	Roche Diagnostics	Cat# 03263991190
BCA protein assay kits	Pierce	Cat# 23225
RNA isolation kit	Qiagen	Cat# 74104
Transcriptor Universal cDNA Master Kit	Roche	Cat# 05893151001
Complete protease inhibitor cocktail	Sigma-Aldrich	Cat# P1860
Phosphatase inhibitor cocktail 2	Sigma-Aldrich	Cat# P5726
Phosphatase inhibitor cocktail 3	Sigma-Aldrich	Cat# P0044
Transcriptor universal cDNA master kit	Roche	Cat# 05893151001
ProLong Gold Antifade mountant with DAPI stain	Invitrogen	Cat# P36931
Antigen unmasked solution citrate-based	Vector	Cat# H-3300-250
ADRA1A-Tango plasmid	Addgene	Cat# 66213
AGTR1-Tango plasmid	Àddgene	Cat# 66222
pcDNA 3.1 plasmid	Invitrogen	Cat# 79020
Tamoxifen	Sigma-Aldrich	Cat# T5648
SMC growth cell medium	Lonza	Cat# CC-3182
Polyethyleneimine (PEI)	Polysciences	Cat# 23966
Reconstituted HDL	Meridian Bioscience	Cat# A34275H
Angiotensin II	Bachem	Cat# 4006473
TrypLE Express	Gibco	Cat# 12604013

Foxp3 / Transcription Factor Staining Buffer Set	Invitrogen	Cat# 00-5523-00
EnVisision Dako Target retrieval	Dako	Cat# K800521-2
Vector Red	Vector	Cat# SK-5100
Tissue Tek optimal cutting temperature (OCT) compound	Sakura Finetek	Cat# 4583
hematoxylin	Merck	Cat# 1.04302
Aluminum Potassium Sulfate-dodecahydrate	Merck	Cat# 1.01047
Sodium Iodate	Merck	Cat# 1.06525
Chloral Hydrate	Merck	Cat# 1.02425
Citric acid	Merck	Cat# 1.00241
Aquatex	Merck	Cat# 1.08562.0050
goat serum	Sanquin Amsterdam, The Netherlands	Cat# M1530
DAB	Sigma-Aldrich	Cat# D5637
Cholestanol-D5	MedChemExpress	Cat# HY-107819
Intercellular fixation&permeabilization buffer set	eBioscience	Cat# 88-8824-00
phosphate buffered paraformaldehyde	Alfa Aesar	Cat# 43368
xylene	VWR	Cat# 28973.363
ethanol	VWR	Cat# 20821.365
eosin	Merck	Cat# 1.5935
Permount	Fisher Scientific	Cat# SP15-500
hematoxylin	Klinipath	Cat# 800620
Iron(III)chloride	Sigma Aldrich	Cat# 157740
Picric acid solution	Sigma Aldrich	Cat# P6744-1GA
Glacial acetic acid solution	Merck	Cat# 1.00063.1000
Trilogy	Cell Marque	Cat# 920P-09

Software

Description	Source	Persistent ID / URL

DOI [to be added]

Hamamatsu Nanozoomer 2.0-HT Slide Scanner	Hamamatsu Photonics, Hamamatsu City, Japan	N/A
Aperio Image Scope	version 12.3.3, Leica	Aperio ImageScope | Pathology Slide Viewing Software (leicabiosystems.com)
Image J Software	NIH	ImageJ 1.51k_Java 1.6.0_24, imagej.net
BioTek Synergy H4 Hybrid Multi-Mode Microplate Reader	Biotek Instruments	N/A
FACS DiVa software V8.0.3	BD Biosciences	https://www.bdbiosciences.com/en-se/products/instruments/software-informatics/instrument-software/facs-diva/facsdiva-software-v-8-0-3-upgrade-win-7-32-bit-os.659528
FlowJo version 10.6.2	FlowJo	https://www.flowjo.com/solutions/flowjo/downloads
Zen 3.4	Zeiss	https://www.zeiss.com/microscopy/us/products/microscope-software/zen-lite.html
Graph Pad Prism version 8	GraphPad Software	https://www.graphpad.com/scientificsoftware/prism
R version 4.3.1	CRAN	https://www.r-project.org/
G*Power 3.1.9.7	HHU	Universität Düsseldorf: G*Power (hhu.de)

DOI [to be added]

ARRIVE GUIDELINES

This study involves use of animal models:
Yes

Manuscripts reporting on animal research are expected to adhere to the ARRIVE guidelines. A completed ARRIVE reporting guideline checklist will be required prior to acceptance and the information should be clearly presented in the manuscript. The following shortened version of the checklist should be completed at this time; a completed ARRIVE checklist is required to be uploaded during revision submission.

Animals
Species, age, sex, strain, and sources of animals are described: Yes

Randomization
Randomization and allocation concealment were performed and described: Yes

Blinding
Blinding was performed and the process described:
Yes

Inclusions and Exclusions (a)
Specific criteria for inclusions and exclusions are specified:
Yes

Inclusions and Exclusions (b)
Criteria for inclusions and exclusion were set before the study:
Yes

Reporting of Excluded Animals
All animals excluded after the randomization are reported: N/A

Statistical Methods Statistical Methods are described: Yes

Date completed 19/03/2024 16:01

DOI [to be added]
