# Supplementary material for: SMC Abca1 and Abcg1 Deficiency Enhances Urinary Bladder Distension but Not Atherosclerosis
Source: Circ Res. 2025 Feb 11;136(5):491–507. doi: 10.1161/CIRCRESAHA.124.325103 (PMC11867804; doi:10.1161/CIRCRESAHA.124.325103)
Supplement: Supplementary file 4 [file res-136-491-s004.pdf]

SUPPLEMENTAL MATERIAL

SMC Abca1 and Abcg1 deficiency enhances urinary bladder distension but not atherosclerosis

Benedek Halmos[1,*], Anouk M. La Rose[1,*], Daisey Methorst[1], Anouk G. Groenen[1], Dalibor Nakladal[2,3,4], Venetia Bazioti[1], Mirjam H. Koster[1], Niels J. Kloosterhuis[1], Azuwerus van Buiten[2], Elisabeth M. Schouten[5], Nicolette C.A. Huijkman[1], Miriam Langelaar-Makkinje[1], Laura Bongiovanni[1,6,7], Simon M. De Neck[1,6], Alain de Bruin[1,6], Hendrik Buikema[2], Leo E. Deelman[2], Marius C. van den Heuvel[8], Folkert Kuipers[1,9], Igle Jan de Jong[10], Judith C. Sluimer[11], Helle F. Jørgensen[12], Robert H. Henning[2], Marit Westerterp[1,†]

[1]Department of Pediatrics, [2]Department of Clinical Pharmacy and Pharmacology, [5]Department of Cardiology, [8]Department of Pathology, [9]Department of Laboratory Medicine, and [10]Department of Urology, University Medical Center Groningen, University of Groningen, Groningen, The Netherlands; [3]Comenius University Science Park, Bratislava, Slovakia, [4]5th Department of Internal Medicine, Faculty of Medicine, Comenius University Bratislava, Bratislava, Slovakia; [6]Department of Biomolecular Health Sciences, Dutch Molecular Pathology Center, University of Utrecht, Utrecht, The Netherlands; [7]Department of Veterinary Medicine, University of Teramo, Teramo, Italy; [11]Department of Pathology, Cardiovascular Research Institute Maastricht, Maastricht University Medical Center, the Netherlands. Department of Medical Clinic II for Kidney and Hypertension Diseases, Rheumatological and Immunological Diseases, Rheinisch-Westfälische Technische Hochschule (RWTH) Aachen, Germany. British Heart Foundation (BHF) Centre for Cardiovascular Sciences, University of Edinburgh, United Kingdom;[12] Section of Cardiorespiratory Medicine, Department of Medicine, University of Cambridge, Cambridge Biomedical Campus, Cambridge, UK.

[*] These authors contributed equally to this work

[†]Corresponding author: Marit Westerterp PhD (m.westerterp@umcg.nl)

Expanded Materials and Methods

Online Tables S1-4

Online Figures S1-17

References 63-72

Expanded Materials and Methods

Animals

Mice with floxed genes for the cholesterol transporters ATP binding cassette A1 and G1 *(Abca1*$^{fl/fl}$*Abcg1*$^{fl/fl}$*)* (stock 021067),[63] Myosin heavy chain 11 (*Myh11)Cre*^{ERT2} (stock 019079)[64] and low-density-lipoprotein-receptor deficient (*Ldlr*$^{-/-}$*)* (stock 002207) mice were purchased from Jackson Laboratories. *Myocardin*$^{fl/fl}$ (*Myocd*$^{fl/fl}$) mice were a kind gift from Dr. Eric Olson (UT Southwestern). All mice were in the C57Bl6/J background. Since the *Myh11Cre*^{ERT2} transgene is located on the Y chromosome, male mice were included in experiments. *Abca1*$^{fl/fl}$*Abcg1*$^{fl/fl}$*Ldlr*$^{-/-}$ and *Myh11Cre*^{ERT2} mice were intercrossed to generate *Myh11Cre*^{ERT2}*Abca1*$^{fl/fl}$*Abcg1*$^{fl/fl}$*Ldlr*$^{-/-}$*, Myh11Cre*^{ERT2}*Abca1*$^{fl/fl}$*Ldlr*$^{-/-}$*, Myh11Cre*^{ERT2}*Abcg1*$^{fl/fl}$*Ldlr*$^{-/-}$, and *Myh11Cre*^{ERT2}*Ldlr*$^{-/-}$ mice that are referred to as *SMC-Abc*^{dko}*Ldlr*$^{-/-}$, *SMC-Abca1*^{ko}*Ldlr*$^{-/-}$*, SMC-Abcg1*^{ko}*Ldlr*$^{-/-}$, and *Ldlr*$^{-/-}$ mice, respectively. At 6 weeks of age, mice were fed a diet containing 400 mg/kg tamoxifen citrate (TD. 55125.I, Envigo) for 1 week to induce activation of *Myh11Cre*^{ERT2}, followed by a 2 week recovery period on chow diet (V1554, Ssniff Spezialdiäten GmbH). Subsequently, mice were fed a chow diet or Western-type diet (WTD) (40% fat, 0.15% cholesterol; D12079B, Research Diets) for 6 to 16 weeks.

In addition, *Myh11Cre*^{ERT2} mice were crossbred with *Myocd*$^{fl/fl}$ mice to generate *Myh11Cre*^{ERT2}*Myocd*$^{fl/fl}$ mice. At 12 weeks of age, mice received 10 injections of tamoxifen (once daily; 1 mg each in corn oil) to induce activation of *Myh11Cre*^{ERT2}. *Myh11Cre*^{ERT2}*Myocd*$^{fl/fl}$ mice are referred to as *SMC-Myocd*^{ko} mice.

For all studies, mice were housed under standard laboratory conditions with a light cycle of 12 hours and *ad libitum* water and food. Mice were randomized to experimental groups according to genotype. The number of mice used for the experiments and period of diet (chow or WTD) feeding are indicated for each experiment in the figure legends. No inclusion or exclusion criteria were used. For all measurements, the individual carrying out these was unaware of the genotypes (blinded). All animal studies were approved by the Institutional Animal Care and Use Committee from the University of Groningen under permit number AVD105002015244 and adhered to the guidelines set out in the 2010/63/EU directive or the Cambridge Animal Welfare and Ethical Review Body under project license P452C9545.

mRNA expression in thoracic aorta and urinary bladder SMCs

The thoracic aorta and urinary bladder from *Ldlr*$^{-/-}$ and *SMC-Abc*^{dko}*Ldlr*$^{-/-}$ mice were isolated and homogenized using the Precellys evolution tissue homogenizer (Bertin technologies). Subsequently, the thoracic aorta was incubated with a digestion mix containing liberase TH (5401135001, Sigma-Aldrich) (4 U/mL), DNAse I (DN25, Sigma-Aldrich) (40 U/mL), and hyalurodinase (H3506, Sigma-Aldrich) (60 U/mL) in PBS for 1 hour at 37°C. For RNA extraction and cDNA synthesis from thoracic aorta SMCs, samples were first resuspended in RLT buffer. RNA was then extracted using the RNeasy Mini Kit (74104, Qiagen), without using DNase, and cDNA was synthesized using the Transcriptor Universal cDNA Master Kit (05893151001, Roche). *Abca1* and *Abcg1* mRNA expression in thoracic aorta SMCs and actin alpha 2 (*Acta2),* myocardin *(Myocd),* tropomyosin 1 *(Tpm1),* myosin heavy chain 11 (*Myh11*), calponin 1 (*Cnn1),* vascular cell adhesion molecule 1 (*Vcam-1*), lymphocyte antigen 6a (*Ly6a*), cluster of differentiation 68 *(Cd68),* galectin-3 *(Mac-2* or *Lgals3),* collagen type 1 alpha chain 1 *(Col1a1), Col1a2, Col2a1,* metalloproteinase 2 *(Mmp2), Mmp3*, lumican *(Lum),* tissue inhibitor of metalloproteinase 1 (*Timp1),* chondroadherin *(Chad),* SRY-box transcription factor 9 *(Sox9),* Krüppel-like factor 4 *(Klf4),* transcription factor 21 *(Tcf21),* triggering receptor expressed on myeloid cells 2 (*Trem2*), secreted phosphoprotein 1 *(Spp1), Cd11c, Cd9,* MER proto-oncogene tyrosine kinase *(Mertk),* liver x receptor α *(Lxrα),* lysosomal acid lipase *(Lipa),* Niemann-Pick C1 *(Npc1),* cathepsin b *(Ctsb), Ctsl*, *Cd36,* macrophage scavenger receptor 1 *(Msr1), Cd163,* fatty acid binding protein 2 *(Fabp2), Fabp4, Fabp5, Mmp12, Mmp14,* interleukin (*Il*)*-1β*, NLR family pyrin domain containing 3 (*Nlrp3*), *Il-6*, interferon α and β receptor subunit 1 *(Ifnar1),* tumor necrosis factor α (*Tnfα*), monocyte chemoattractant protein-1 (*Mcp-1*), C-C motif chemokine receptor 2 *(Ccr2),* C-C motif chemokine ligand 3 *(Ccl3), Cd14,* arachidonate 5-lipoxygenase *(Alox5),* toll-like receptor 2 *(Tlr2),* C-X-C motif chemokine ligand 1 *(Cxcl1), Cxcl2,* G protein-coupled receptor 183 *(Gpr183),* transforming growth factor β receptor 2 *(Tgfβr2),* arginase 1 *(Arg1),* interferon regulatory factor 7 *(Irf7),* ISG15 ubiquitin like modifier *(Isg15), Cd115,* lymphatic vessel endothelial hyaluronan receptor 1 *(Lyve1)*, Acylcoenzyme A:cholesterol acyltransferase 1 (*Acat1*), *Acat2*, glucose regulated protein 78 (*Grp78*), C/EBP homologous protein (*Chop*), growth arrest and DNA damage-inducible protein 34 (*Gadd34*), endoplasmic reticulum protein 72 (*Erp72*), X-box binding protein 1 (*Xbp1*), spliced *Xbp1*, B-cell lymphoma 2 associated transcription factor 1 (*Bclaf1*), proliferating cell nuclear antigen (*Pcna*), and Mindbomb homolog-1 (*Mib1*) mRNA expression in thoracic aorta or urinary bladder SMCs were assessed by qPCR using QuantStudio 7 Flex Real-Time PCR System (Applied Biosystems). The settings of the system were: denaturation, 95°C for 15 seconds; melting, annealing, and elongation, 60°C for 1 minute; for a total of 40 cycles. Initial differences in RNA

quantity were corrected for using the housekeeping genes *Gapdh* and *m36B4*, employing the ΔΔCt method. Primer sequences and amplicon sizes are shown in the Major Resource Table.

Human bladder SMC cholesterol loading and mRNA expression

Primary human bladder SMCs (BdSMCs) were purchased from Lonza (CC-2533) (Breda, The Netherlands) and cultured in SMC growth cell medium (CC-3182, Lonza) according to the manufacturer's instructions. Cells were cultured for a maximum of 5 passages. After incubation with or without cyclodextrin-cholesterol (C4951) (50 µg/mL cholesterol; Sigma) for 24 or 48 hours, cells were lysed, and RNA was isolated and cDNA was synthesized as described above. *ACTA2*, *MYOCD*, *MAC-2* (*LGALS3*), *CD68*, and *KLF4* mRNA expression were assessed by qPCR as described above. Initial differences in RNA quantity were corrected for using the housekeeping genes *CYCLOA* (*PPIA*) and *B-ACTIN*.

Oil Red O and Smooth Muscle Actin staining on frozen sections

The thoracic aorta and heart from *Ldlr*$^{-/-}$ and *SMC-Abc*^{dko}*Ldlr*$^{-/-}$ mice were isolated, embedded in Tissue-Tek optimal cutting temperature (OCT) compound (Sakura Finetek 4583) and immediately frozen on dry ice. Seven µm frozen cross-sections were made. To assess neutral lipids, sections were stained with Oil Red O, and counterstained with hematoxylin (Merck, 1.04302). In brief, 0,5 gram of Oil Red O (O0625, Sigma-Aldrich) was dissolved in 100 mL of isopropanol using the very gentle heat of a water bath (60˚C, covered), and subsequently filtered. Then, 30 mL of this stock stain was added to 20 mL of H_2O, allowed to stand for 10 mins, filtered, and covered immediately. Sections were stained immediately (the stain does not keep and needs to be prepared fresh for each use). The solution containing hematoxylin (for subsequent counterstaining) was prepared by first boiling 2,5 L of H_2O, and then dissolving in 2,5 L of hot H_2O: 2,5 g hematoxylin (Merck, 1.04302), 125 g Aluminum Potassium Sulfate-dodecahydrate (Merck, 1.01047), 0,5 g Sodium Iodate (Merck, 1.06525), 125 g Chloral Hydrate (Merck, 1.02425), and 2,5 g Citric Acid (Merck, 1.00241). The solution was subsequently cooled down to room temperature (RT), left overnight at RT, and filtered. Sections stained for Oil Red O were stained in this hematoxylin solution for 4 min, then left in running tapwater for 10 min, before mounting them employing Aquatex (Merck 1.08562.0050). Subsequently, scans of the sections were made using a Hamamatsu NanoZoomer 2.0-HT Slide Scanner (Hamamatsu Photonics, Hamamatsu City, Japan) employing a 40x objective. Photos were made using Aperio Image Scope (version 12.3.3, Leica, Aperio ImageScope | Pathology Slide Viewing Software (leicabiosystems.com)), and subsequently Oil Red O$^+$ area was quantified employing ImageJ software (ImageJ 1.51k_Java 1.6.0_24, imagej.net, NIH). To assess aortic

smooth muscle cell (SMC) content, sections were blocked in 10% goat serum (M1530, Sanquin Amsterdam, The Netherlands) for 30 min at room temperature. Subsequently, slides were incubated o/n at 4°C with anti-smooth muscle actin (α-SMA) (RB9010P1; Lab Vision) (1/200 dilution) primary antibody. Then, sections were incubated with biotinylated goat anti-rabbit secondary antibody (BA-1000; Vector Laboratories) (1/250 dilution) for 30 min at room temperature. Subsequently, sections were incubated with Vectastain ABC-peroxidase (PK-4000; Vector Laboratories) according to the manufacturer's instructions, stained with DAB (Sigma, D5637) and counterstained with hematoxylin.

Gas Chromatography – Mass Spectrometry

Thoracic aorta and bladder SMCs were isolated as described above, sonicated, and 100 µl of the internal standard cholestanol D5 (HY-107819; MedChemExpress) was added to the cells prior to cholesterol extraction using hexane. Samples were split for measurement of either total or free cholesterol content using Gas Chromatography – Mass Spectrometry (7890B GS system, 5973 MS system, and 7693A automatic liquid sampler from Agilent; positive chemical ionization mode with 5% ammonia in methane as reaction gas). A polar DB-WAXetr (30m x 0.25 mm x 0.25 µm) column was used. Cholesteryl ester (CE) content was calculated by subtracting free cholesterol from total cholesterol. Cholesterol content was normalized to cellular protein content measured by BCA protein assay (23225; Pierce).

Plasma total cholesterol

Blood samples were collected and plasma was separated by centrifugation. For mice fed WTD, blood samples were diluted 15 times. Total plasma cholesterol was measured using an enzymatic kit (113009910026; Diasys Diagnostic Systems), by adding 70 µL of PBS to 5 µL of plasma. Cholesterol standard FS (113009910030; Diasys Diagnostic Systems) was used for the calibration curve. 10 µL of standards or samples were pipetted into a 96 wells plate, and subsequently 200 µL of R1 reagent from the enzymatic kit was added. Samples and reagents were mixed by gently tapping the plate and subsequently incubate at 37˚C for 30 mins. The absorbance at λ=540 nm was then measured employing a BioTek Synergy H4 Hybrid Multi-Mode Microplate Reader (Biotek Instruments).

Vascular function studies

The thoracic aorta from *Ldlr*$^{-/-}$, *SMC-Abca1*^{ko}*Ldlr*$^{-/-}$, *SMC-Abcg1*^{ko}*Ldlr*$^{-/-}$, and *SMC-Abc*^{dko}*Ldlr*$^{-/-}$ mice was isolated and placed in cold Krebs bicarbonate solution (120.4 mM NaCl, 5.9 mM

KCl, 2.5 mM $CaCl_2$, 1.2 mM $MgCl_2$, 1.2 mM NaH_2PO_4, 11.5 mM glucose, 25 mM $NaHCO_3$), cleaned of adhering tissue and cut into equally sized aortic rings (2 mm in length) at the exact same location of each aorta. Aortic rings were mounted on 200 µm stainless steel pins in individual organ baths of a Mulvany myograph (Model 610M, Danish Myo Technology), as described previously.[65,66] Briefly, organ baths containing 6 mL Krebs solution were maintained at 37°C and continuously aerated with 95% O_2 - 5% CO_2 to maintain pH at 7.4. Aortic rings were equilibrated for 40 min until they were at steady state baseline in terms of isometric force, and then stretched to transmural pressure of 13.3 kPa using the proprietary DMT Normalization Procedure software module. Rings were primed and checked for viability by two consecutive stimulations with a high concentration of potassium chloride (KCl, 60 mM) followed by intermediate washes using Krebs buffer, and renewed stabilization. To study vasoconstriction, aortic rings were stimulated with cumulative concentrations of phenylephrine (PE, 10 nM - 100 µM) (P6126; Sigma-Aldrich). After the highest concentration of PE, a single high dose of KCl (60 mM) was added to determine maximal receptor-independent constriction.
PE-induced vasoconstriction was calculated as % of the final KCl-induced vasoconstriction. For these experiments, first, vasoconstriction was examined in aortic rings of *Ldlr$^{-/-}$ vs SMC-Abc^{dko}Ldlr$^{-/-}$* mice, and then in aortic rings of *Ldlr$^{-/-}$ vs SMC-Abca1^{ko}Ldlr$^{-/-}$* and *Ldlr$^{-/-}$ vs SMC-Abcg1^{ko}Ldlr$^{-/-}$* mice. In all experiments employing aortic rings from *SMC-Abca1^{ko}Ldlr$^{-/-}$* and *SMC-Abcg1^{ko}Ldlr$^{-/-}$* mice, aortic rings from *SMC-Abc^{dko}Ldlr$^{-/-}$* mice were used as positive controls. For this reason, the data of the four groups of mice are presented together in one graph.

For experiments examining the effect of membrane cholesterol depletion on G-protein coupled receptor-mediated vasoconstriction, aortic rings were incubated with 10 mM methyl-β-cyclodextrin (C4555; Sigma-Aldrich) (MβCD; in Krebs buffer)[5] or vehicle for 45 minutes, and washed with Krebs buffer to remove MβCD, prior to subsequent stimulation with cumulative doses of either PE (10 nM - 100 µM), serotonin (H7752; Sigma-Aldrich) (10 nM – 100 µM), or U46619 (D8174; Sigma-Aldrich) (1 nM - 10 µM). In each of these experiments, a single high dose of KCl (60 mM) was added after the highest dose of each stimulus, and receptor-mediated vasoconstriction was calculated as % of this KCl-induced vasoconstriction.

To study vasorelaxation, aortic rings were first pre-constricted with 30 nM U46619. Once vasoconstriction had stabilized, increasing concentrations of acetylcholine (ACh, 10 nM - 100 µM) were added in a cumulative fashion to assess endothelium-dependent vasorelaxation, or sodium nitroprusside (71778; Sigma-Aldrich) (SNP, 1 nM - 100 µM) as a nitric oxide (NO) donor

to assess SMC dependent vasorelaxation. Vasorelaxation induced by Ach or SNP is expressed as % of preconstriction with U46619.

Blood pressure measurement

Blood pressure was assessed in *Ldlr$^{-/-}$* and *SMC-Abc^{dko}Ldlr$^{-/-}$* mice fed a WTD for 16 weeks as previously described.[67] Mice were anaesthetized and a catheter (Mikro-Tip pressure catheter 1.2F, Transonic Scisense Inc) was inserted via the left carotid artery into the aorta and blood pressure was recorded using a Powerlab data acquisition system (AD Instruments). Subsequently, the catheter was moved into the left ventricle of the heart to record blood pressure. Data was analyzed using LabChart 8 software.

Aortic SMC α_1-adrenergic receptor binding

Specific binding of ligands to the α_1-adrenergic receptor (α_1-AR) was assessed in primary aortic SMCs from *Ldlr$^{-/-}$* and *SMC-Abc^{dko}Ldlr$^{-/-}$* mice. To isolate aortic SMCs, the thoracic aorta was digested in Dulbecco's Modified Eagle Medium (DMEM) containing 1 mg/mL collagenase type II (LS004176; Worthington Biochemical) at 37°C for 1 hour, minced, and incubated in DMEM containing 0.125 mg/mL elastase type III (E0127; Sigma-Aldrich) at 37°C for 40 minutes. Cells were grown in DMEM containing 10% FBS and 1% penicillin/streptomycin and passaged once before experiments. Aortic SMCs were seeded in a 96-wells plate and washed with HBSS. Specific binding to the α_1-AR was assessed in a competitive radioligand binding assay using the α_1-AR ligands prazosin and phentolamine. Total and non-specific binding in the same SMC cultures were assessed by incubating cells with the α_1-AR ligand prazosin that was ^{3}H-labeled ([7-methoxy-^{3}H]-prazosin) (NET823250UC; PerkinElmer) at a concentration of 100 nM without and with addition of a 1,000-fold higher concentration of unlabeled phentolamine (100 µM) (local hospital pharmacy, UMCG), respectively. Cells were incubated with compounds for 1 hour at 37°C, after which they were quickly washed with ice-cold PBS (3x), preserving receptor binding, and lysed using 1N NaOH. The cell lysate was transferred to scintillation vials and ^{3}H-activity was counted for 5 minutes. Technical triplicates were used for each experimental condition. Specific α_1-AR binding was calculated by subtracting the ^{3}H-activity in the lysate of the [7-methoxy-^{3}H]-prazosin+phentolamine condition from the [7-methoxy-^{3}H]prazosin condition. The assay was validated using chinese hamster ovary (CHO) cells overexpressing the α_{1a}-AR.[68]

α_{1a}-AR expression in HEK293T cells

Human embryonic kidney 293-derived cells expressing the SV40 large T antigen (HEK293T) were cultured in DMEM (Gibco) supplemented with 10% FCS, and 1% penicillin/streptomycin. The ADRA1A-Tango plasmid expressing human α_{1a}-AR and a FLAG epitope at the N-terminus allowing for the detection of α_{1a}-AR cell surface expression was obtained from Addgene (Cat no 66213). HEK293T cells were transfected with either empty vector pcDNA3.1 (control; 79020, Invitrogen) or the ADRA1A-Tango plasmid using polyethyleneimine (PEI, 23966, Polysciences); ratio plasmid: PEI = 1:4. At 24 hours after transfection, cells were incubated with or without reconstituted HDL (rHDL, 50 µg/mL; A34275H, Meridian BioScience) for another 24 hours. Subsequently, cells were stimulated with or without PE (10^{-4} M) for 30 mins. Cells were trypsinized with TrypLE Express (12604013, Gibco) for 2 min at 37°C, and subsequently centrifuged (1500 g; 5 min, 4°C). The pellet was resuspended in Dulbecco's phosphate-buffered saline (DPBS; Gibco) and centrifuged at 1500g for 2 min at 4°C. This washing step was performed two times. After the last centrifugation step, cells were resuspended in Fluorescence-activated Cell Sorting (FACS buffer: Hank's Balanced Salt Solution, 0.1% Bovine Serium Albumin (BSA), 0.5mM Ethylenediaminetetraacetic acid (EDTA)) and stained with mouse anti-FLAG primary antibody (5 µg/mL; F1804, Sigma-Aldrich) for 30 min at 4°C in the dark. Cells were centrifuged at 1500g for 2 min at 4°C. The supernatant was aspirated and the pellet was resuspended in FACS buffer. This washing step was performed two times. Cells were then stained with goat anti-mouse APC (5 µg/mL; A-865, ThermoFisher) for 30 min at 4°C in the dark, subsequently centrifuged at 1500g for 2 min at 4°C, and resuspended in 300 µL FACS buffer. APC positive staining was assessed on a LSRII (BD Biosciences), running FACSDiVa software (BD Biosciences FACSDiva software v 8.0.3 Upgrade (Win 7 32 bit OS) (bdbiosciences.com)). The data were analyzed using FlowJo software (FlowJo version 10.6.2).

To assess total (surface+intracellular) α_{1a}-AR expression in the same experiment, cells were, after incubation with rHDL, fixed and permeabilized. In brief, cells were resuspended in fixation/permeabilization buffer of the intercellular fixation&permeabilization buffer set (88-8824-00, eBioscience), incubated on ice in the dark (10 min), and washed in permeabilization buffer of the same buffer set. Cells were then centrifuged (1500 g; 5 min, 4°C), and the pellet was resuspended in permeabilization buffer. This washing step was performed two times. After the last step, cells were resuspended in permeabilization buffer and stained with goat anti-mouse APC as described above.

Alternatively, at 48 h after transfection and 24 h after rHDL incubation, cells were stimulated with PE (10^{-4} M). After PE stimulation for 1, 5, 10, 15, 20, or 30 minutes, cells were harvested

and lysed in radio immuno precipitation assay (RIPA) buffer (1% IGEPAL, 0.1% SDS, and 0.5% sodium deoxycholate in PBS) supplemented with complete protease inhibitor cocktail (P1860, Sigma-Aldrich), and phosphatase inhibitor cocktail 2 and cocktail 3 (P5726 and P0044, Sigma-Aldrich). Cell lysates were separated by SDS-PAGE gel electrophoresis and immunoblotted with primary antibodies against total extracellular signal-regulated kinases (ERK) 1/2 (1:1.000; 4695, Cell Signaling Technology) or phosphorylated ERK1/2 (1:1.000; 4370, Cell Signaling Technology). Heat shock protein 90 (HSP90) (1:1.000; 4874, Cell Signaling Technology) was used as loading control. Goat anti-rabbit (1:10.000; 1706515, Bio-rad) HRP-conjugated secondary antibody was used to detect ERK1/2, phosphorylated ERK1/2, and HSP90. Blots were developed using the Chemidoc XRS+ system (Bio-Rad, Berkeley, CA) and the relative intensity of the bands was quantified using ImageJ software (ImageJ 1.51k_Java 1.6.0_24, imagej.net, NIH).

Angiotensin II receptor type I expression in HEK293A cells

HEK293-derived cells expressing the SV40 large A antigen (HEK293A) were cultured as described for HEK293T cells above. The AGTR1-Tango plasmid expressing human AGTR1 and a FLAG epitope at the N-terminus allowing for the detection of AGTR1 cell surface expression was obtained from Addgene (Cat no. 66222). Transfections were carried out as described above for the ADRA1A-Tango plasmid. At 24 hours after transfection, cells were incubated with or without rHDL (50 µg/mL) and during the last 30 minutes of this incubation period with or without angiotensin II (100 nM; 4006473, Bachem). The cells were then trypsinized and analyzed by flow cytometry as described above for the experiments with the ADRA1A-Tango plasmid.

Atherosclerotic lesion analysis

After 16 weeks of WTD feeding, mice were sacrificed, hearts were isolated and fixed in 4% phosphate buffered paraformaldehyde (43368, Alfa Aesar). Hearts were embedded in paraffin, 4 µm sections of the aortic root area were made and stained with haematoxylin-eosin (H&E). In brief, after paraffin melting (15 min, 60˚C oven), slides were kept in xylene (28973.363, VWR) for (2x 2 mins), and then hydrated in solutions of decreasing % of ethanol (20821.365, VWR): 100% ethanol (2x, 2 mins), 96% ethanol (2x, 2 mins), 70% ethanol (2x, 2 mins), before leaving them in H_2O for 2 mins. Sections were then stained for hematoxylin (see preparation of hematoxylin solution above) for 4 mins; subsequently slides were left under running tapwater for 10 mins, sections were stained for eosin (preparation: dissolve 25 g eosin (Merck 1.5935) in 1250 mL H_2O; wait until eosin has been dissolved completely and then add 1250 mL ethanol

96% and 1.25 mL of acetic acid) for 1 min, left in H_2O for 10 seconds, and then dehydrated in solutions of increasing % of ethanol: 70% ethanol (2x, 2 mins), 96% ethanol (2x, 2 mins), 100% ethanol (2x, 2 mins), after leaving them in xylene (2x, 2 mins). Sections were mounted in permount mounting media (SP15-500, Fisher Scientific). This mounting media was the same for all immunohistochemistry stainings, except for Oil Red O, and immunofluorescent stainings where specific mounting media is indicated. The method for scanning the sections and obtaining photos has been described above (Oil Red O staining paragraph) and is similar for all sections, except when using immunofluorescence. Atherosclerotic lesion area was quantified using ImageJ software (NIH) and the average of 5 sections with 40 μm distance in between was calculated for each mouse. Necrotic core area was identified as acellular area, lacking nuclei and cytoplasm, similar to previous measurements,[64] quantified using ImageJ software, and expressed as μm^2 area and as % of total atherosclerotic lesion area. To assess fibrous cap content and collagen area, Sirius Red staining was performed. Sections were stained with Weigert's haematoxylin (prepared by adding 1 g hematoxylin (Klinipath, 800620) to 100 mL of 95% ethanol and subsequently combining this with 100 mL of a solution containing 4 mL 29% ferric chloride in H_2O (stock: 7,29 g Iron(III) chloride (Sigma Aldrich, 157740) in 25 mL H_2O), 95 mL H_2O, and 1 mL HCl (37%)) for 8 min to stain the nuclei and subsequently with Sirius Red (Direct Red 80, 365548, Sigma Aldrich) (0.1% (w/v)) in 1.3% aqueous picric acid solution (P6744-1GA, Sigma Aldrich)) for 1 hour. Slides were then washed twice 10 sec in acidified water (1% glacial acetic acid solution; prepared by adding 5 mL of stock solution (Merck 1.00063.1000) to 1L H_2O). Fibrous cap thickness was measured in the largest section at even intervals 2 μm apart using ImageJ software. The average thickness of the fibrous cap was reported in length units, as described previously.[69] Sirius Red$^+$ area was quantified using ImageJ software and expressed as μm^2 area and as % of total atherosclerotic lesion area. To assess smooth muscle cell area, sections were stained with α-SMA as described above for the thoracic aorta. In addition to α-SMA, we also stained for Transgelin (Tagln; SM22α; ab10135, Abcam) to assess smooth muscle area. Antigen retrieval was performed using heat-induced epitope retrieval at low pH with EnVision Dako target retrieval solution (Dako K800521-2), followed by blocking in 10% normal goat serum. Sections were incubated with anti-SM22α (1/400 dilution) primary antibody for 30 minutes. Subsequently, sections were incubated with donkey anti-goat (A16005; ThermoFisher) (1/500 dilution) secondary antibody for 30 minutes, before being stained with DAB and counterstained with hematoxylin. Pseudo-fluorescent images were created for SM22α-stained sections and scanned with the PANNORAMIC 1000 (3DHistech). Both α-SMA$^+$ and SM22α$^+$ area were quantified using ImageJ software and expressed as μm^2 area and as % of total atherosclerotic lesion area. To assess Lgals3$^+$ SMC and macrophage area, antigen retrieval was performed by incubating sections in Trilogy (920P-

09, Cell Marque) (15 min; microwave). Next, sections were blocked for 30 min at RT and incubated o/n at 4°C with Mac-2 (Lgals3) primary antibody (CL8942AP, Cedarlane) (1/1000 dilution). Then, the sections were incubated with biotinylated goat anti-rat secondary antibody (BA-9400, Vector Laboratories) (1/125 dilution) for 30 min at RT and subsequently with Vectastain ABC-peroxidase according to the manufacturer's instructions. Sections were then stained with DAB and counterstained with haematoxylin. Alternatively, sections were stained with anti-rat IgG-AF488 (1/200 dilution; A-11006, Thermo Fisher), and mounted using ProLong Gold Antifade Mountant with DAPI (P36931, Invitrogen). For immunofluorescence, sections were imaged using an AxioObserver Z1 compound microscope (10x objective; AxioCam MRm3 CCD camera; Carl Zeiss) running Zen software. Mac-2$^+$ area was quantified using ImageJ software (NIH) and expressed as μm^2 area and as % of total atherosclerotic lesion area. To assess modified SMC and fibroblast area, sections were stained for Lumican. Antigen retrieval was performed as stated above for SM22α. Sections were incubated with anti-Lumican (Ab 168348, Abcam) (1/5.000 dilution) for 30 minutes at RT, and subsequently incubated with Brightvision anti-rabbit HRP (DPVR55HRP, VWR) for 30 minutes before being counterstained with hematoxylin. Pseudo-fluorescent images were created and scanned with PANNORAMIC 1000, and Lumican$^+$ area was quantified using ImageJ software and expressed as μm^2 area and as % of total atherosclerotic lesion area. To assess chondrocytes, sections were stained for SRY-box transcription factor 9 (Sox9). Antigen retrieval was performed as mentioned above for SM22α. Sections were incubated with anti-Sox9 (ab185230, Abcam) (1/2000 dilution) for 60 minutes and subsequently incubated with Brightvision anti-rabbit HRP for 30 minutes. Sections were developed with vector red (SK-5100, Vector) and counterstained with hematoxylin. Sections were scanned with the PANNORAMIC 1000. Sox9$^+$ area was quantified using ImageJ software and expressed as number of intimal and medial cells as well as % of total intimal and medial cells. To confirm the absence of nonspecific binding by the secondary antibody, we carried out stainings employing the secondary antibody without the primary antibody. In addition, we assessed co-localization of SMA$^+$ and Mac-2$^+$ area. Antigen retrieval was performed using citrate (H-3300, Vector) (1 mM; 15 minutes microwave). We stained sections concomitantly with α-SMA-Cy3 antibody (dilution 1/500) and anti-Mac-2 (Lgals3) (dilution 1/1000) as described above for the Mac-2 staining, and subsequently for 60 min at RT with anti-rat IgG-AF647 (1/100 dilution; A-21247, Thermo Fisher). Sections were mounted using ProLong Gold Antifade Mountant with DAPI (P36931, Invitrogen). Sections were imaged using an AxioObserver Z1 compound microscope (10x objective; AxioCam MRm3 CCD camera; Carl Zeiss) running Zen software. The AF647 was visualized as fluorescent green in the sections. The co-localization of SMA$^+$ and Mac-2$^+$ area was quantified as % of SMA$^+$ or Mac-2$^+$ (Lgals3$^+$) area using ImageJ software (NIH). In addition, from the

same mice, the brachiocephalic artery (BCA) was isolated and fixed in 4% phosphate buffered paraformaldehyde. BCAs were embedded in paraffin, 6 µm sections of the BCA were made and stained with H&E. Atherosclerotic lesion area was quantified using ImageJ software (NIH) and the average of 5 sections with 60 µm distance in between was calculated for each mouse. Lesion composition was assessed employing exactly the same stainings as described above for the aortic root.

Urinary bladder volume

Ldlr$^{-/-}$, *SMC-Abca1*^{ko}*Ldlr*$^{-/-}$, *SMC-Abcg1*^{ko}*Ldlr*$^{-/-}$, and *SMC-Abc*^{dko}*Ldlr*$^{-/-}$ mice were sacrificed. The length and width (in both directions) of the urinary bladder were measured in mm using a ruler and volume in mm^3 was converted to mL. Concomitantly, urinary volume was collected from the bladder using a syringe. For 20 measurements, the calculation based on the volume in mm^3 and the volume assessed using a syringe in mL matched exactly, and therefore we used the measurements in mm^3 to estimate urinary bladder volume.

Plasma creatinine levels

Blood from *Ldlr*$^{-/-}$ and *SMC-Abc*^{dko}*Ldlr*$^{-/-}$ mice was collected by cardiac puncture and plasma was separated by centrifugation. Plasma creatinine levels were measured using the enzymatic creatinine plus VER.2 assay and Cobas 501 analyzer according to manufacturer's instructions (Roche Diagnostics).

Tamsulosin treatment

After 2 weeks of WTD feeding, *Ldlr*$^{-/-}$ and *SMC-Abc*^{dko}*Ldlr*$^{-/-}$ mice were treated with the $\alpha_1$adrenergic receptor antagonist tamsulosin (T1330, Sigma-Aldrich) or vehicle (PBS) in the drinking water (0.4 mg/kg body weight) for 4 weeks. After 4 weeks of tamsulosin treatment, mice were sacrificed and urinary bladder volume was assessed as described above.

Histology of urinary bladder, urethra and kidney

The urinary bladder, urethra and kidneys of *Ldlr*$^{-/-}$ and *SMC-Abc*^{dko}*Ldlr*$^{-/-}$ mice were isolated and fixed in 4% phosphate buffered paraformaldehyde. Tissues were embedded in paraffin, 4 µm sections were made and stained with H&E. Bladder wall thickness at the location where the bladder has the largest diameter was quantified every 500 µm using ImageJ software to calculate the average bladder wall thickness per mouse. To assess whether the urethra was dilated, the surface area of the lumen of the urethra was measured using ImageJ software. To

assess urinary bladder and urethra SMC area, the anti-smooth muscle actin staining was carried out as described above for the thoracic aorta. To assess urinary bladder and urethra collagen area, sections were stained with Sirius Red as described above for the aortic root. To assess urinary bladder Lgals3^{+} SMC and macrophage area, sections stained with antiMac-2 (Lgals3) as described above for the aortic root. In addition, urinary bladders of *Myh11Cre*^{ERT2}*Myocd*$^{fl/fl}$ mice were isolated and fixed in 4% phosphate buffered paraformaldehyde. Tissues were embedded in paraffin, 4 μm sections were made and stained with H&E. Bladder wall thickness was assessed as described above.

Statistical analysis

All data are presented as mean ± SEM. Datasets were assessed for normality using the Shapiro-Wilk test. Datasets smaller than n=6 were assumed to be non-normally distributed, with the exception of mRNA expression levels in Figures 5, 7 and SVIII in the data supplement, which were assessed for normality with n≥5. Outliers were identified in normally and non-normally distributed datasets using Grubb's test and the ROUT test (Q=1%), respectively, and removed from further analysis. To compare 2 groups, the unpaired t-test was used when both datasets were normally distributed. When data were not normally distributed, the Mann-Whitney U test was used. To compare ≥ 3 groups, a One-way Analysis of Variance (ANOVA), followed by a Bonferroni post hoc test was performed when all datasets were normally distributed. A Kruskal-Wallis test with Dunn's test with Bonferroni adjustment was used when data were not normally distributed. Concentration-response curves were compared by two-way ANOVA with Sidak's multiple comparison test. Kidney interstitial inflammation score was compared by Chi-square test. Group size and statistical test are reported in the figure legends. The criterion for significance was set at $P<0.05$. Statistical analysis was performed using GraphPad Prism 8 and R version 4.3.1.

Power Calculations

All power calculations were performed using G*Power 3.1.9.7 software. For atherosclerosis studies, we hypothesized, based on previous studies suggesting that low *Abca1* expression in intimal SMCs contributes to SMCs gaining markers of macrophages, and that 50% of plaque foam cells are of SMC origin,[10,11,26] that SMC-*Abca1/Abcg1* deficiency increased atherosclerotic lesion area. Our previous studies showed that when macrophage *Abca1/Abcg1* deficiency did not affect plasma cholesterol levels, macrophage *Abca1/Abcg1* deficiency increased atherosclerotic lesion area by ~65%.[63] In this model of macrophage *Abca1/Abcg1* deficiency, *Abca1* and *Abcg1* mRNA expression in lesion CD68^{+} cells were below the detection limit.[63] Based on previous studies suggesting that 50% of foam cells are of SMC origin, we

hypothesized that SMC-*Abca1/Abcg1* deficiency would increase atherosclerotic lesion area by 33% (so 50% of the 65% increase in atherosclerotic lesion area in mice with macrophage *Abca1/Abcg1* deficiency). Calculating with a lesion size in the *Ldlr*$^{-/-}$ control group of 350000 µm^2 (based on previous studies)[70] and a lesion size in the experimental group of 465500 µm^2 (~33% increase in atherosclerotic lesion size) and a standard deviation of 90000 µm^2 (based on previous studies),[70] a power calculation showed that at least n=11 mice would be required for an 80% chance to detect a difference where p<0.05. For all other atherosclerosis studies (lesion characterization, single SMC-*Abca1* or SMC-*Abcg1* deficiency), we used at least n=11 mice per group.

For the level of deletion of *Abca1* and *Abcg1* mRNA expression in SMCs of *MyhCre*^{ERT2}*Abca1*$^{fl/fl}$*Abcg1*$^{fl/fl}$*Ldlr*$^{-/-}$ mice *vs MyhCre*^{ERT2}*Ldlr*$^{-/-}$ controls, we hypothesized, based on previous studies in the *MyhCre*^{ERT2} model[4,33] that the decrease in *Abca1/Abcg1* mRNA expression in SMCs would be ~60% with a standard deviation of ~30%. Calculating with *Abca1* and *Abcg1* mRNA expression in control *MyhCre*^{ERT2}*Ldlr*$^{-/-}$ mice being 1 and in *MyhCre*^{ERT2}*Abca1*$^{fl/fl}$*Abcg1*$^{fl/fl}$*Ldlr*$^{-/-}$ mice being 0.4 and a standard deviation of 0.3, a power calculation showed that at least n=6 mice would be required for an 80% chance to detect a difference where p<0.05.

For the level of Oil Red O staining and cholesteryl ester and free cholesterol accumulation in SMCs, we hypothesized, based on our previous studies on *Abca1/Abcg1* deficiency in endothelial cells, macrophages, and T cells,[63,71,72] that SMC-*Abca1/Abcg1* deficiency would increase these levels by ~2-fold. Calculating with 0.1% of SMCs in aortic rings of *Ldlr*$^{-/-}$ control mice being positive for Oil Red O and 0.2% being positive in SMC-*Abc*^{dko}*Ldlr*$^{-/-}$ mice, and a standard deviation of 0.04%, a power calculation showed that at least n=4 mice would be required for an 80% chance to detect a difference where p<0.05. We hypothesized that the level of differences between these values would be similar for cholesteryl ester and free cholesterol accumulation (even though the range and unit is different).

For vasoconstriction experiments, we hypothesized, based on *in vitro* experiments that membrane cholesterol depletion reduces α_1-adrenergic surface expression by ~40%,[5] that membrane cholesterol accumulation due to SMC-*Abca1/Abcg1* deficiency would increase maximum vasoconstriction to the α_1-adrenergic receptor agonist PE by 80%. Calculating with a vasoconstriction of 30% of the maximum response to KCl for the *Ldlr*$^{-/-}$ controls, and 50% for *SMC-Abc*^{dko}*Ldlr*$^{-/-}$ mice, and a standard deviation of 10%, a power calculation showed that at least n=6 mice would be required for an 80% chance to detect a difference where p<0.05.

Since we expected that effects on blood pressure would be due to increased vasoconstriction, we used the same number of mice for blood pressure experiments.

For experiments assessing specific binding to the α_1-adrenergic receptor on aortic SMCs, we hypothesized that, similar to effects on vasoconstriction, membrane cholesterol accumulation due to SMC-*Abca1/Abcg1* deficiency would increase specific binding by 80%. Calculating with a specific binding of 1 in SMCs from *Ldlr*$^{-/-}$ controls and 1,8 in SMCs from *SMC-Abc*^{dko}*Ldlr*$^{-/-}$ mice, and a standard deviation of 0,5, a power calculation showed that at least n=8 mice would be required for an 80% chance to detect a difference where $p<0.05$.

For experiments assessing the effect of reconstituted HDL (rHDL) on the surface expression of the α_1-adrenergic receptor on HEK293 cells we hypothesized that similar to experiments employing membrane cholesterol depletion by methyl-β-cyclodextrin,[36] rHDL would decrease the surface expression of the α_1-adrenergic receptor by 50%. Calculating with a surface α_1-adrenergic receptor expression of 1000 mfi in the control setting, and 500 mfi in the presence of rHDL, and a standard deviation of 220 mfi, a power calculation showed that at least n=5 replicates would be required for an 80% chance to detect a difference where $p<0.05$. Since ERK phosphorylation occurs downstream of the α_1-adrenergic receptor, we anticipated the same effect (though in a different range) for this parameter. For experiments employing HEK cells transfected with a construct expressing the angiotensin II receptor type 1, we employed the same power calculation as for HEK cells transfected with a construct expressing the α_1-adrenergic receptor.

For measurements of bladder volume, we included all mice that we used for atherosclerosis and vasoconstriction experiments. We did not carry out a power calculation, but included as many mice as possible. For experiments employing tamsulosin, we hypothesized that the effect of SMC-*Abca1/Abcg1* deficiency on bladder distension was completely dependent on the α_1-adrenergic receptor, and that we would be able to inhibit ~50% of all α_1-adrenergic receptors in the bladder. Calculating with a bladder volume of 0.4 mL for *SMC-Abc*^{dko}*Ldlr*$^{-/-}$ mice and 0.2 mL for *SMC-Abc*^{dko}*Ldlr*$^{-/-}$ mice treated with tamsulosin and a standard deviation of 0.16 mL, a power calculation showed that at least n=12 mice would be required for an 80% chance to detect a difference where $p<0.05$.

For experiments on bladder histology, and mRNA expression in bladder SMCs, we hypothesized that similar to experiments on SMC cholesterol loading with cyclodextrin-cholesterol,[24] bladder SMCs from *SMC-Abc*^{dko}*Ldlr*$^{-/-}$ mice would lose ~55% of their contractile

markers compared to *Ldlr*$^{-/-}$ control mice with a standard deviation of ~30%. Based on these parameters, a power calculation shows that at least n=5 mice would be required for an 80% chance to detect a difference where $p<0.05$. A similar number of replicates was included for experiments on bladders from *Myocd*$^{+/+}$ and *SMC-Myocd*^{ko} mice as well as for aortic SMCs, where we initially had expected similar observations as compared to bladder SMCs with SMC-*Abca1/Abcg1* deficiency.

Materials

Please see the Major Resources Table in the supplemental materials.

Table S1. Effect of SMC *Abca1/Abcg1* deficiency on plasma cholesterol levels in *Ldlr*$^{-/-}$ mice fed WTD for 16 weeks.

Genotype	Plasma cholesterol level (mg/dL)
***Ldlr*$^{-/-}$**	**890 ± 47.1**
***SMC-Abca1*^{ko}*Ldlr*$^{-/-}$**	**885 ± 28.6**
***SMC-Abcg1*^{ko}*Ldlr*$^{-/-}$**	**938 ± 69.8**
***SMC-Abc*^{dko}*Ldlr*$^{-/-}$**	**822 ± 60.1**
**Genotype**	**Bodyweight (g)**
***Ldlr*$^{-/-}$**	**33.0 ± 0.6**
***SMC-Abc*^{dko}*Ldlr*$^{-/-}$**	**31.6 ± 0.4**

Ldlr$^{-/-}$, *SMC-Abca1*^{ko}*Ldlr*$^{-/-}$, *SMC-Abcg1*^{ko}*Ldlr*$^{-/-}$ and *SMC-Abc*^{dko}*Ldlr*$^{-/-}$ mice were fed WTD for 16 weeks and plasma cholesterol levels were measured (*Ldlr*$^{-/-}$ (n=48), *SMC-Abca1*^{ko}*Ldlr*$^{-/-}$ (n=17), *SMC-Abcg1*^{ko}*Ldlr*$^{-/-}$ (n=14), *SMC-Abc*^{dko}*Ldlr*$^{-/-}$ (n=16)), as was bodyweight (*Ldlr*$^{-/-}$ (n=21), *SMC-Abc*^{dko}*Ldlr*$^{-/-}$ (n=16)). Data are shown as mean ± SEM.

Table S2. EC_{50} values from phenylephrine dose response curves.

Genotype	Diet	Treatment	EC_{50} (*10^{-7} M)	n
$Ldlr^{-/-}$	WTD	-	2.53 ± 0.09	20
SMC-$Abc^{dko}Ldlr^{-/-}$	WTD	-	2.49 ± 0.10	16
SMC-$Abca1^{ko}Ldlr^{-/-}$	WTD	-	1.94 ± 0.12	7
SMC-$Abcg1^{ko}Ldlr^{-/-}$	WTD	-	2.13 ± 0.16	6
$Ldlr^{-/-}$	Chow	-	1.57 ± 0.07	6
SMC-$Abc^{dko}Ldlr^{-/-}$	Chow	-	1.54 ± 0.09	6
$Ldlr^{-/-}$	WTD	-	2.45 ± 0.15	10
$Ldlr^{-/-}$	WTD	MβCD	10.09 ± 0.19	6
SMC-$Abc^{dko}Ldlr^{-/-}$	WTD	-	2.76 ± 0.11	8
SMC-$Abc^{dko}Ldlr^{-/-}$	WTD	MβCD	2.98 ± 0.14	4

EC_{50} values were calculated from dose response curves shown in Figure 2A-B and Figure S3A-B. For each experiment, the number of replicates (n) is shown in the Table. Data are shown as mean ± SEM. WTD, Western-type diet; MβCD, Methyl-β-cyclodextrin.

Table S3. Effect of SMC *Abca1/Abcg1* deficiency on blood pressure in the aorta and left ventricle in *Ldlr*$^{-/-}$ mice fed WTD for 16 weeks.

Genotype	Location	Mean blood pressure (mmHg)	Systolic blood pressure (mmHg)	Diastolic blood pressure (mmHg)
Ldlr$^{-/-}$	Aorta	85.0 ± 3.11	101.2 ± 3.63	70.5 ± 3.37
SMC-Abc^{dko}*Ldlr*$^{-/-}$	Aorta	81.9 ± 5.05	94.7 ± 5.81	69.1 ± 5.17
			Afterload (mmHg)	Preload (mmHg)
Ldlr$^{-/-}$	Left ventricle	47.8 ± 1.33	99.8 ± 3.83	16.5 ± 1.62
SMC-Abc^{dko}*Ldlr*$^{-/-}$	Left ventricle	48.7 ± 3.12	93.3 ± 5.02	19.6 ± 2.57

Ldlr$^{-/-}$ and *SMC-Abc*^{dko}*Ldlr*$^{-/-}$ mice were fed WTD for 16 weeks. Blood pressure in the aorta and left ventricle was assessed using a pressure catheter (n=6). Data are shown as mean ± SEM.

Table S4. Effect of SMC *Abca1/Abcg1* deficiency on plasma cholesterol levels in *Ldlr*$^{-/-}$ mice fed WTD for 16 weeks (atherosclerosis studies).

Genotype	Plasma cholesterol (mg/dL)
Ldlr$^{-/-}$	**1000.8 ± 79.50**
SMC-Abca1^{ko}***Ldlr***$^{-/-}$	**855.9 ± 49.06**
Ldlr$^{-/-}$	**816.5 ± 60.67**
SMC-Abcg1^{ko}***Ldlr***$^{-/-}$	**927.6 ± 75.78**
Ldlr$^{-/-}$	**940.7 ± 85.72**
SMC-Abc^{dko}***Ldlr***$^{-/-}$	**838.0 ± 53.11**

Ldlr$^{-/-}$, *SMC-Abca1*^{ko}*Ldlr*$^{-/-}$, *SMC-Abcg1*^{ko}*Ldlr*$^{-/-}$ and *SMC-Abc*^{dko}*Ldlr*$^{-/-}$ mice were fed WTD for 16 weeks and plasma cholesterol levels were measured (*Ldlr*$^{-/-}$ (n=19), *SMC-Abca1*^{ko}*Ldlr*$^{-/-}$ (n=17), *Ldlr*$^{-/-}$ (n=16), *SMC-Abcg1*^{ko}*Ldlr*$^{-/-}$ (n=14), *Ldlr*$^{-/-}$ (n=17), and *SMC-Abc*^{dko}*Ldlr*$^{-/-}$ (n=16)). . Data are shown as mean ± SEM.

Figure S1

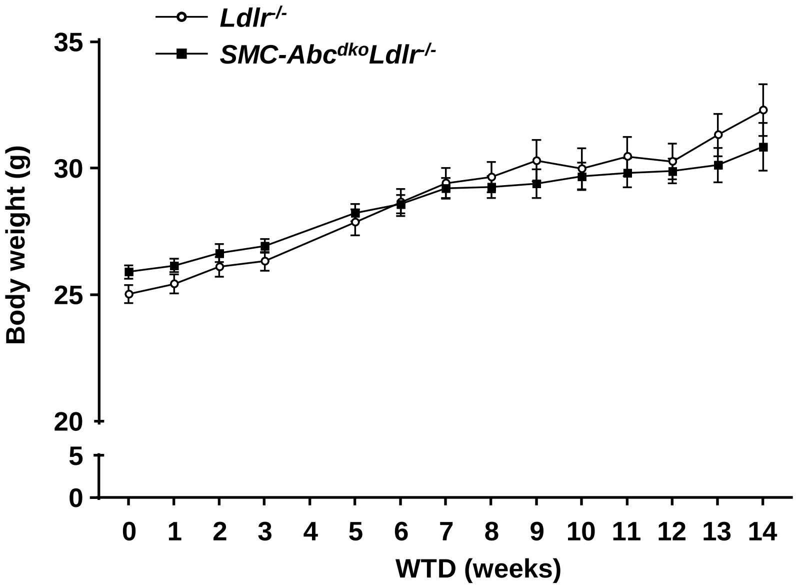

Figure S1. *SMC-Abca1/Abcg1* deficiency does not affect body weight. *Ldlr$^{-/-}$* and *SMC-Abc^{dko}Ldlr$^{-/-}$* mice were fed WTD and weighed at the indicated time points (*Ldlr$^{-/-}$* (n=17), *SMC-Abc^{dko}Ldlr$^{-/-}$* (n=18)). Data are shown as mean ± SEM.

Figure S2

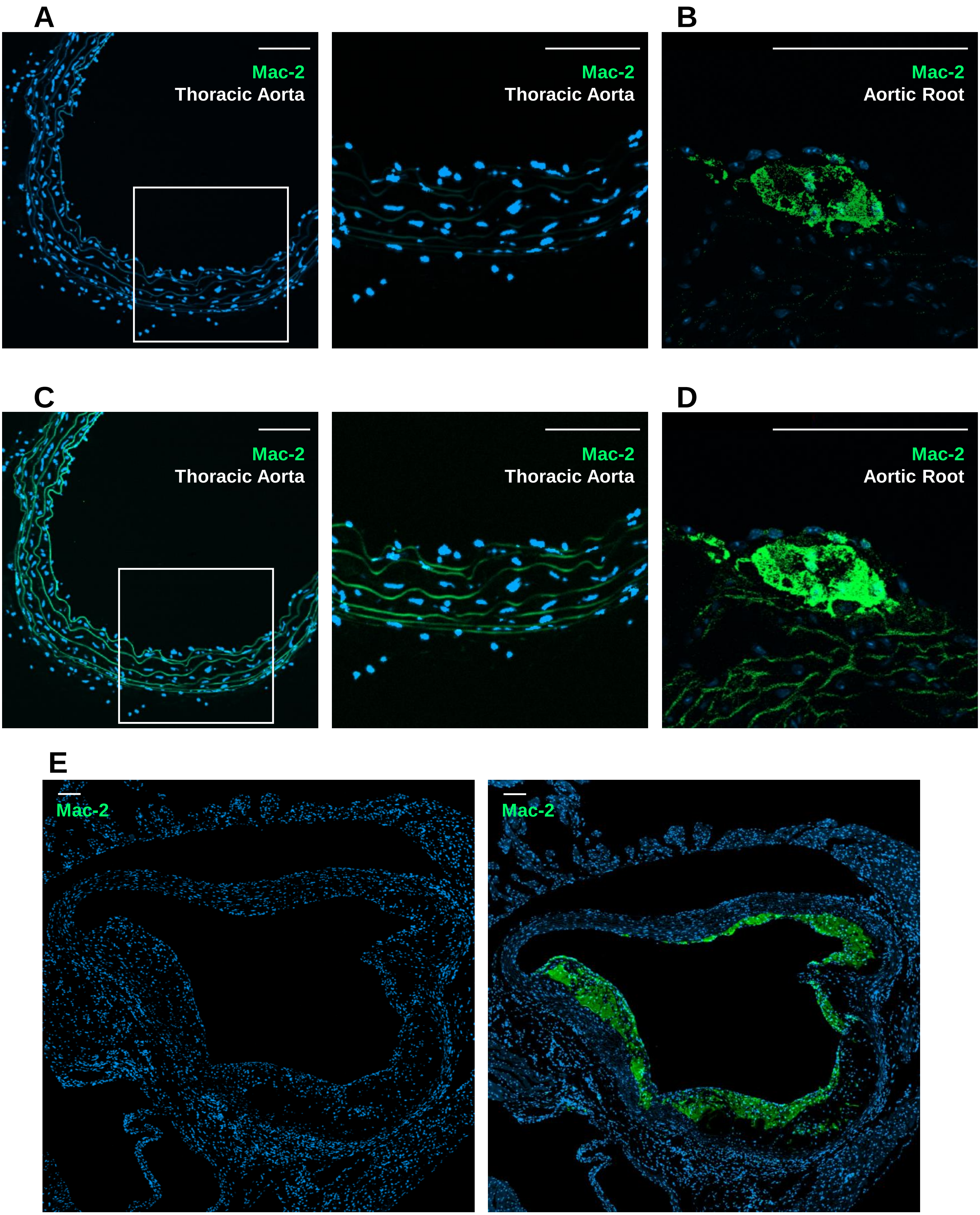
A
Mac-2
Thoracic Aorta
Mac-2
Thoracic Aorta
B
Mac-2
Aortic Root
C
Mac-2
Thoracic Aorta
Mac-2
Thoracic Aorta
D
Mac-2
Aortic Root
E
Mac-2
Mac-2

Figure S2. *SMC-Abca1/Abcg1* deficiency does not induce Mac-2 expression in the thoracic aorta of *Ldlr*$^{-/-}$ mice fed WTD. *Ldlr*$^{-/-}$ and *SMC-Abc*^{dko}*Ldlr-/-* mice were fed WTD for 16 weeks. The thoracic aorta (**A**,**C**) was isolated, sectioned, and stained for Mac-2 and DAPI. At the same time point, the aortic root (**B, D**) was isolated, and used as a positive control for Mac-2 staining. (**A, B**) Mac-2 staining of the thoracic aorta and aortic root at an exposure where the aortic root shows specific Mac-2 staining in plaque macrophages. The same exposure was used for sections of the thoracic aorta and the aortic root. (**C, D**) Mac-2 staining of the thoracic aorta and aortic root at an exposure where the aortic root shows non-specific staining of the elastica (in addition to plaque Mac-2 staining). Using the same exposure, the thoracic aorta also shows non-specific staining of the elastica. (**E**) Negative control for Mac-2 staining in a section of the aortic root (left, negative control without Mac-2 primary antibody; right, adjacent section stained with Mac-2 primary antibody). Scale bar represents 100 µm.

Figure S3

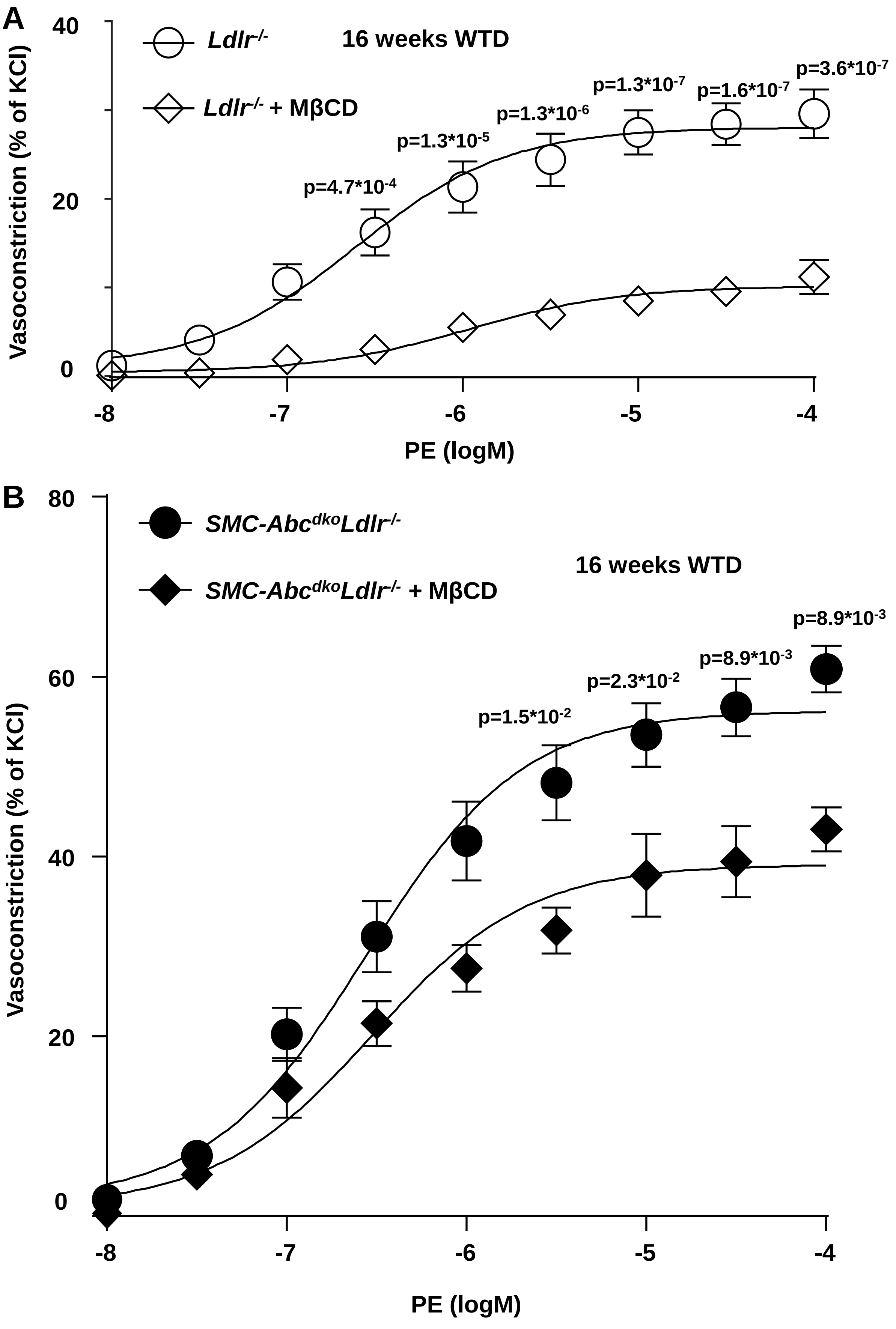
A
40
20
0
Vasoconstriction (% of KCl)
Ldlr-/-
Ldlr-/- + MβCD
16 weeks WTD
p=4.7*10-4
p=1.3*10-5
p=1.3*10-6
p=1.3*10-7
p=1.6*10-7
p=3.6*10-7
-8
-7
-6
-5
-4
PE (logM)
B
80
60
40
20
0
Vasoconstriction (% of KCl)
SMC-AbcdkoLdlr-/-
SMC-AbcdkoLdlr-/- + MβCD
16 weeks WTD
p=1.5*10-2
p=2.3*10-2
p=8.9*10-3
p=8.9*10-3
-8
-7
-6
-5
-4
PE (logM)

Figure S3 (continued)

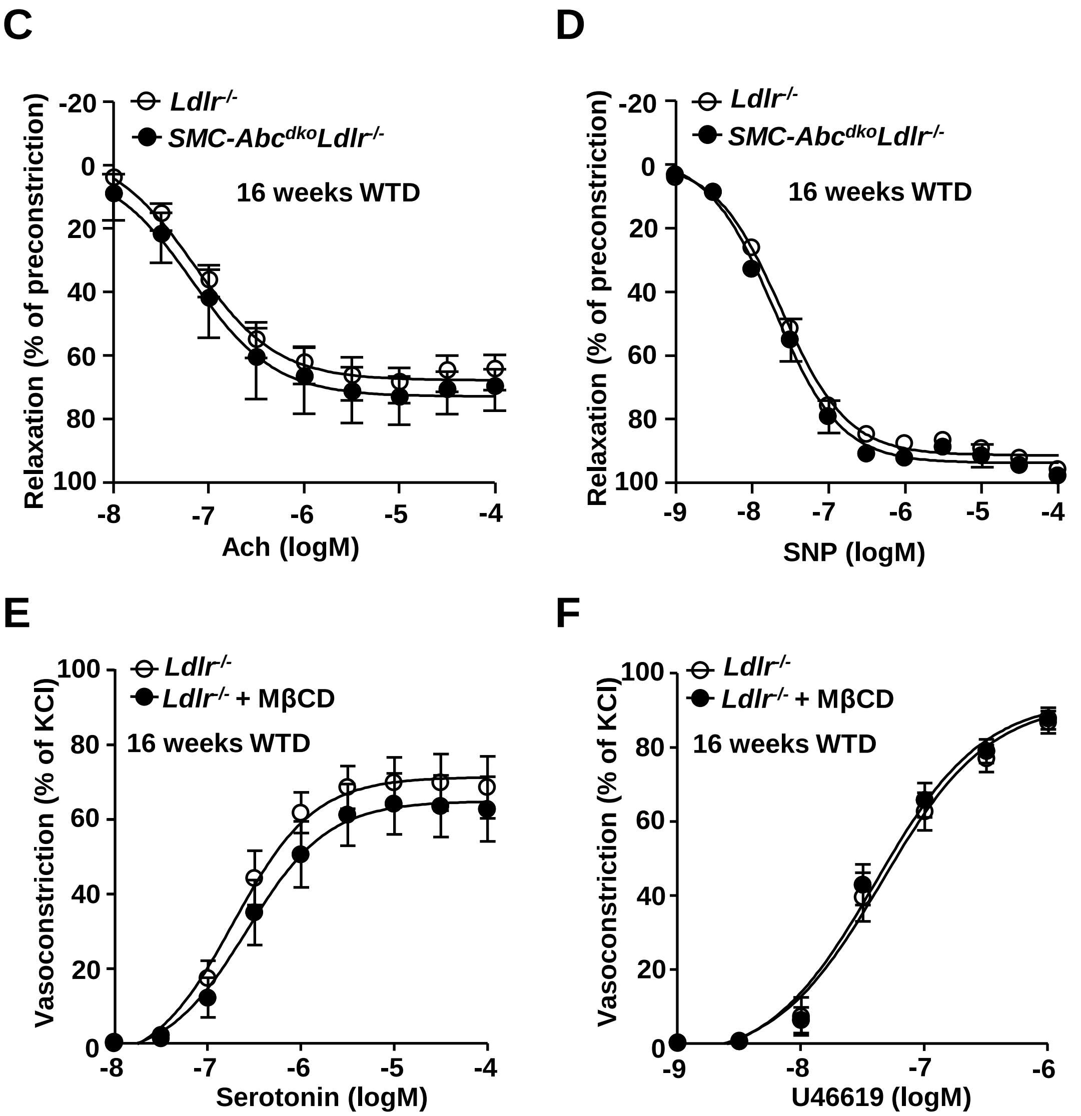

Figure S3. Effects of SMC *Abca1/Abcg1* deficiency and/or membrane cholesterol depletion on parameters related to vascular function. (**A**-**D**) *Ldlr*$^{-/-}$ and *SMC-Abc*^{dko}*Ldlr*$^{-/-}$ mice were fed WTD for 16 weeks. Aortic rings were isolated, mounted on a wire myograph, and pre-incubated with or without methyl-β-cyclodextrin (MβCD) (10mM; 45 minutes) to deplete membrane cholesterol from aortic rings of (**A**) *Ldlr*$^{-/-}$ (*Ldlr*$^{-/-}$ (n=10), *Ldlr*$^{-/-}$ + MβCD (n=6)) and (**B**) *SMC-Abc*^{dko}*Ldlr*$^{-/-}$ mice (*SMC-Abc*^{dko}*Ldlr*$^{-/-}$ a (n=8), *SMC-Abc*^{dko}*Ldlr*$^{-/-}$ + MβCD (n=4)), before subjecting them to increasing concentrations of phenylephrine (PE). (**C**-**D**) Aortic rings were preconstricted with $3*10^{-8}$ M U46619. Vasorelaxation in response to increasing doses of acetylcholine (Ach) (**C**) or Sodium Nitroprusside (SNP) (**D**) was measured (n=4). (**E**, **F**) *Ldlr*$^{-/-}$ mice were fed WTD for 16 weeks. Aortic rings were isolated and vasoconstriction in response to increasing concentrations of serotonin (n=8) (**E**) or U46619 (n=7) (**F**) was measured with or without membrane cholesterol depletion by pre-incubation with methyl-β-cyclodextrin (MβCD) (10mM; 45 minutes). Data are expressed as % of maximum KCl or U46619-induced vasoconstriction. Data are shown as mean ± SEM. *p*-values < 0.05 by two-way ANOVA with Sidak's multiple comparison post-test (**A**, **B**) are indicated on the plots. KCl, potassium chloride.

Figure S4

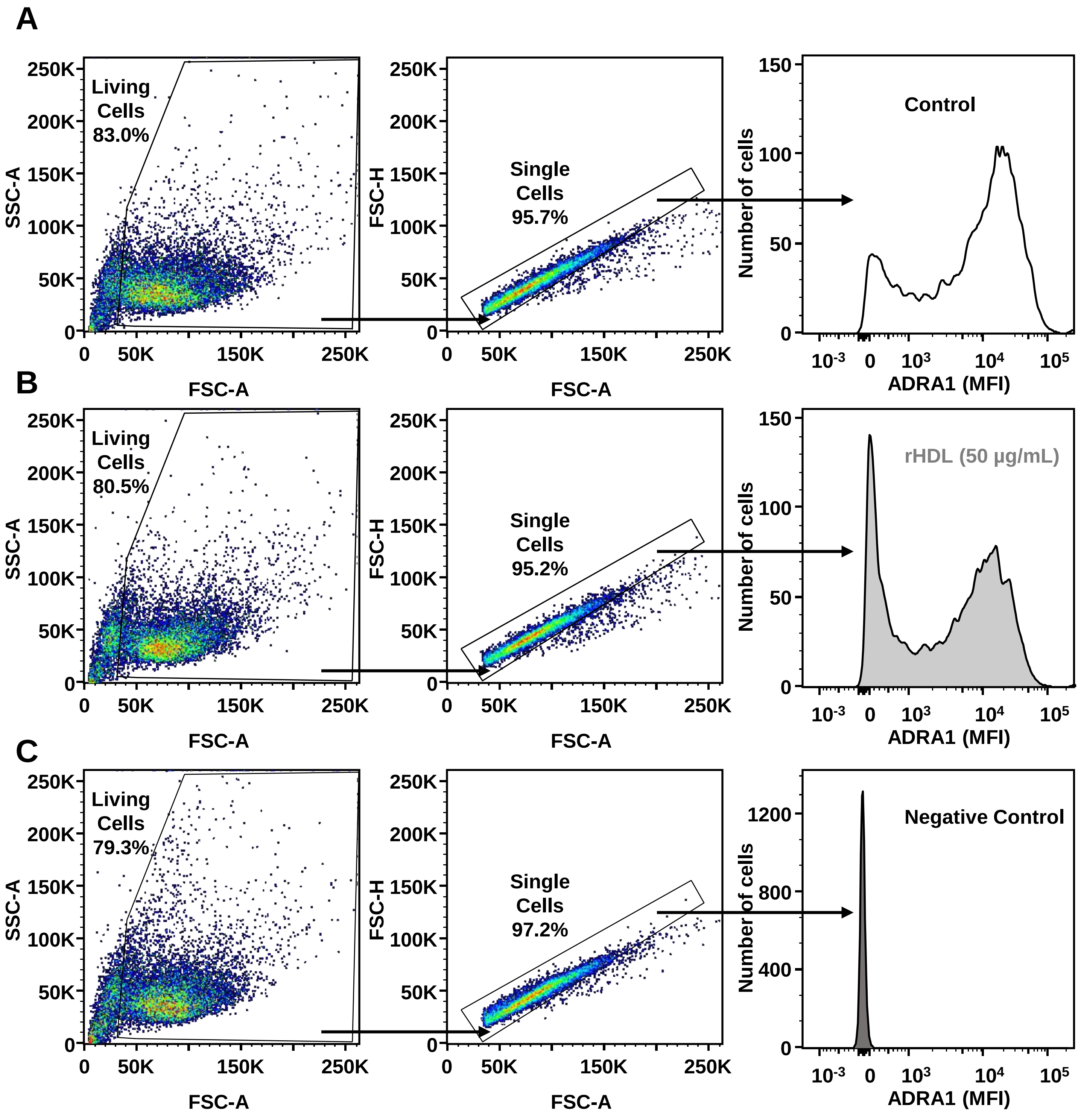
A
B
C
Living Cells 83.0%
Living Cells 80.5%
Living Cells 79.3%
Single Cells 95.7%
Single Cells 95.2%
Single Cells 97.2%
SSC-A
FSC-H
FSC-A
Number of cells
ADRA1 (MFI)
Control
rHDL (50 µg/mL)
Negative Control
0
50K
100K
150K
200K
250K
50
100
150
400
800
1200
10^{-3}
10^{3}
10^{4}
10^{5}

Figure S4 (Continued)

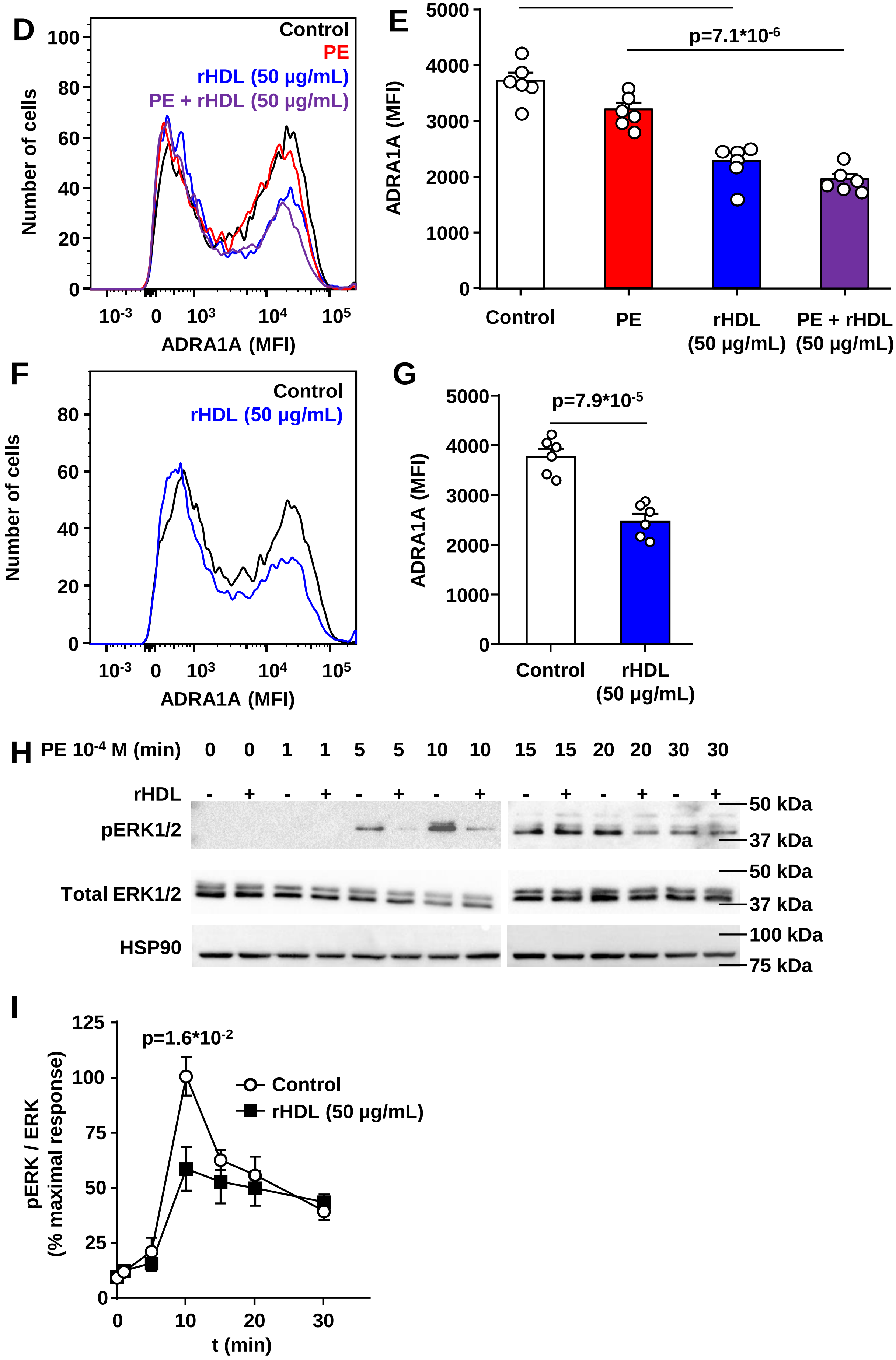

Figure S4 (Continued)

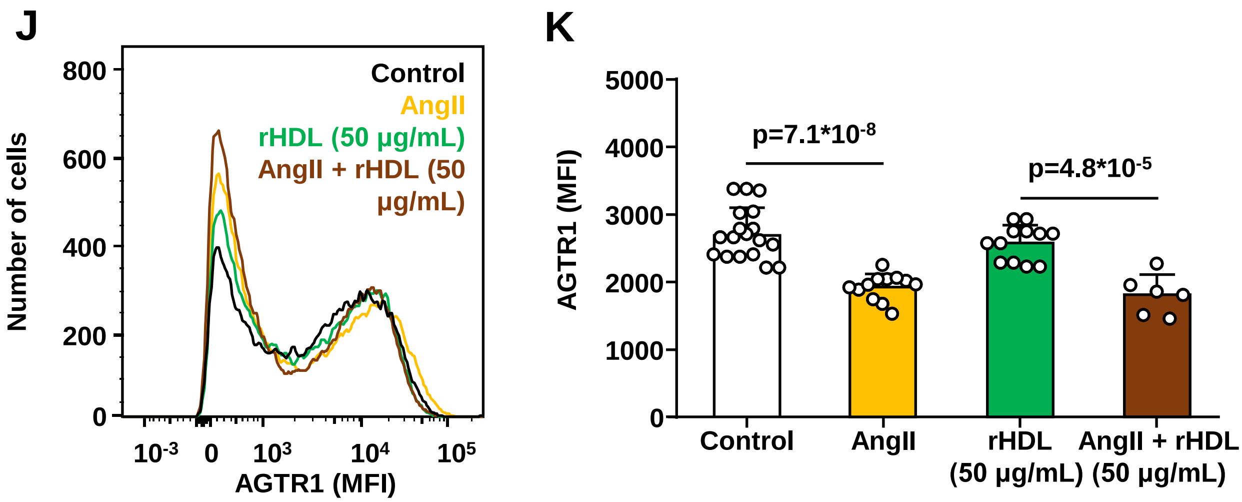

Figure S4. Membrane cholesterol regulates α_1-AR, but not angiotensin II receptor type 1 (AGTR1) surface expression. (**A-I**) HEK293T cells were transfected with the ADRA1A-Tango plasmid expressing human α_{1A}-AR and a FLAG epitope at the N-terminus allowing for the detection of α_{1A}-AR surface expression. (**A-C**) Gating strategy for α_{1A}-AR surface expression. (**A, B**) Gating strategy and representative example of HEK293T cells transfected with the ADRA1A-Tango plasmid. (**C**) Representative example of HEK293T cells transfected with empty plasmid. (**D, E**) Cells were incubated with or without reconstituted HDL (rHDL) for a period of 24 h. During the last 30 min of the rHDL incubation, cells were stimulated with or without PE (10^{-4} M). The surface expression of the α_{1A}-AR was assessed by flow cytometry. (**D**) Representative flow cytometry plot and (**E**) quantification (n=6). (**F**, **G**) Cells were incubated with rHDL for a period of 24 h after which they were fixed and permeabilized. Total expression of the α_1-AR was assessed by flow cytometry. (**F**) Representative flow cytometry plots and (**G**) quantification (n=6). (**H**, **I**) Cells were stimulated with PE and (**H**) ERK phosphorylation was assessed by Western blot at the indicated time points after PE stimulation, and (**I**) quantified (n=5). (**J, K**) HEK293A cells were transfected with the AGTR1-Tango plasmid expressing human AGTR1 and a FLAG epitope at the N-terminus allowing for the detection of AGTR1 surface expression and, 24 h later, incubated with or without rHDL for a period of 24 h, and stimulated with angiotensin II (AngII). The surface expression of the AGTR1 was assessed by flow cytometry. (**J**) Representative flow cytometry plot and (**K**) quantification ((control (n=18), AngII (n=12), rHDL (n=12) AngII + rHDL (n=6)). Data are shown as mean ± SEM. *p*-values < 0.05 by Kruskal-Wallis test with Dunn's post-test with Bonferroni adjustment (**E**), two-tailed unpaired t-test (**G**), Mann-Whitney U test (**I**), or one-way ANOVA with Bonferroni post-test (**K**) are indicated.

Figure S5

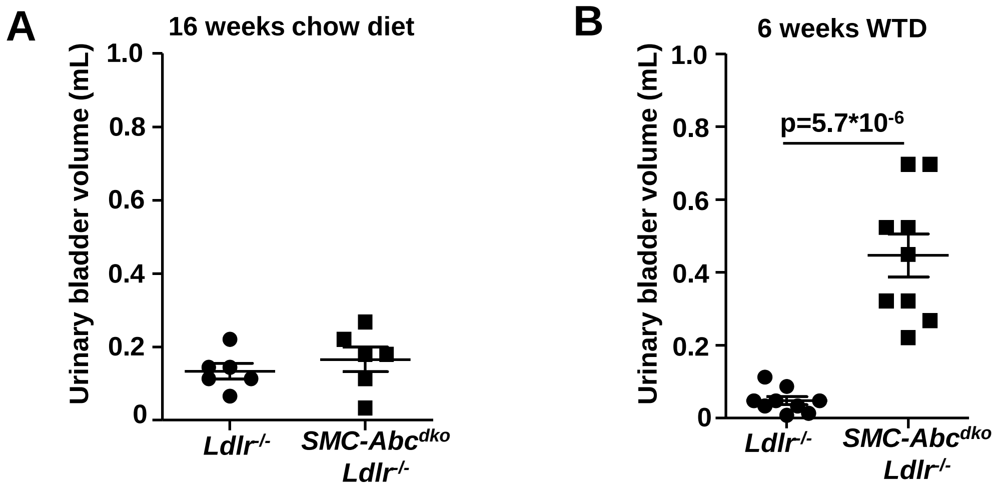

Figure S5. *SMC-Abca1/Abcg1* deficiency increases urinary bladder volume in *Ldlr$^{-/-}$* mice fed WTD for 6 weeks but not in *Ldlr$^{-/-}$* mice fed chow diet. *Ldlr$^{-/-}$* and *SMC-Abc^{dko}Ldlr$^{-/-}$* mice were fed chow diet (**A**) or WTD (**B**). (**A, B**) Urinary bladder volume assessed at 16 weeks of chow diet (n=6) (**A**), and at 6 weeks of WTD (n=9) (**B**). Data are shown as mean ± SEM. *p*-value < 0.05 by two-tailed unpaired t-test is indicated.

Figure S6

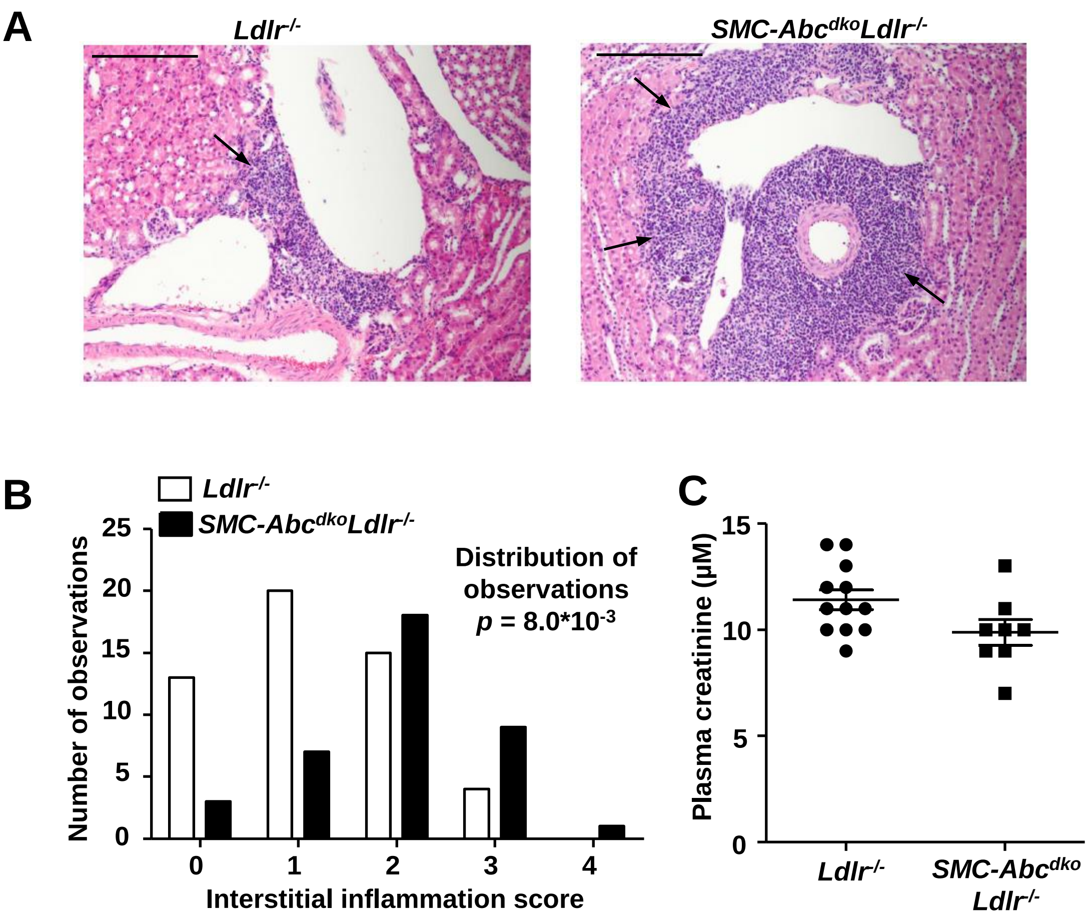

Figure S6. *SMC-Abca1/Abcg1* deficiency induces interstitial inflammation in the kidney of WTD-fed *Ldlr*$^{-/-}$ mice, and does not affect plasma creatinine levels. *Ldlr*$^{-/-}$ and *SMC-Abc*^{dko}*Ldlr*$^{-/-}$ mice were fed WTD 16 weeks. (**A**) Kidneys were isolated, sectioned, and stained with H&E. Scale bar represents 200 µm. (**B**) Quantification of interstitial inflammation score (*Ldlr*$^{-/-}$ (n=26), *SMC-Abc*^{dko}*Ldlr*$^{-/-}$ (n=19)). *p*-value by Chi-square test is indicated. (**C**) Plasma creatinine levels were measured (*Ldlr*$^{-/-}$ (n=12), *SMC-Abc*^{dko}*Ldlr*$^{-/-}$ (n=8)). Data are shown as mean ± SEM.

Figure S7

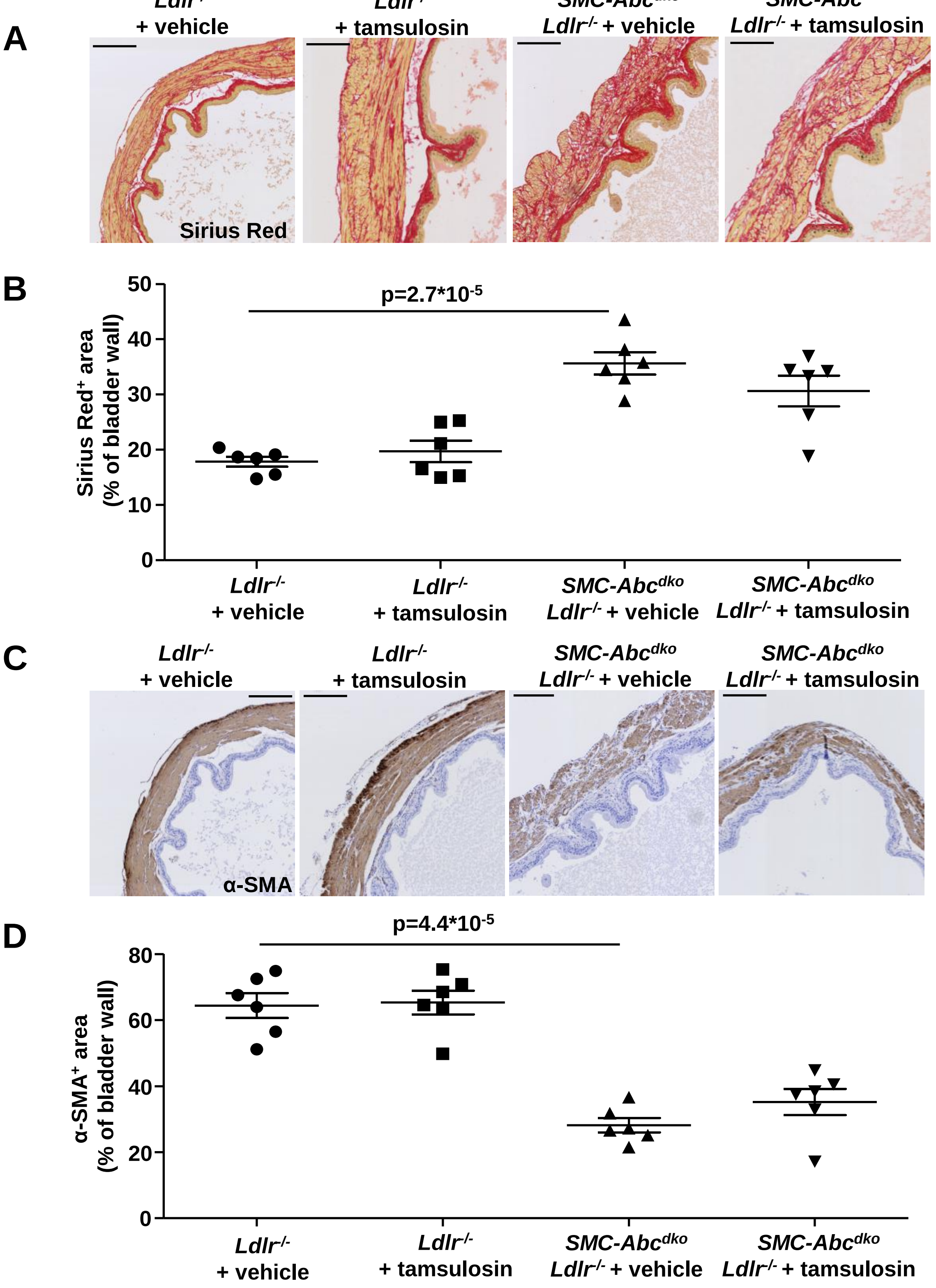

Figure S7. SMC *Abca1/Abcg1* deficiency induces collagen deposition in the urinary bladder and SMC marker loss without reversal by the α_1-AR antagonist tamsulosin. *Ldlr*$^{-/-}$ and *SMC-Abc*^{dko}*Ldlr*$^{-/-}$ mice were fed WTD for 6 weeks. Mice received 0.4 mg/kg tamsulosin or vehicle in the drinking water at 2 weeks after the start of WTD for a period of 4 weeks. (**A**) The urinary bladder was isolated, sectioned, stained with Sirius Red, and (**B**) positive areas were quantified as % of bladder wall area (n=6).(**C**) Sections were stained for α smooth muscle actin (α-SMA), and (**D**) positive areas were quantified as % of bladder wall area (n=6). Scale bar represents 200 μm. Data are shown as mean ± SEM. *p*-values < 0.05 by one-way ANOVA with Bonferroni post-test are indicated.

Figure S8

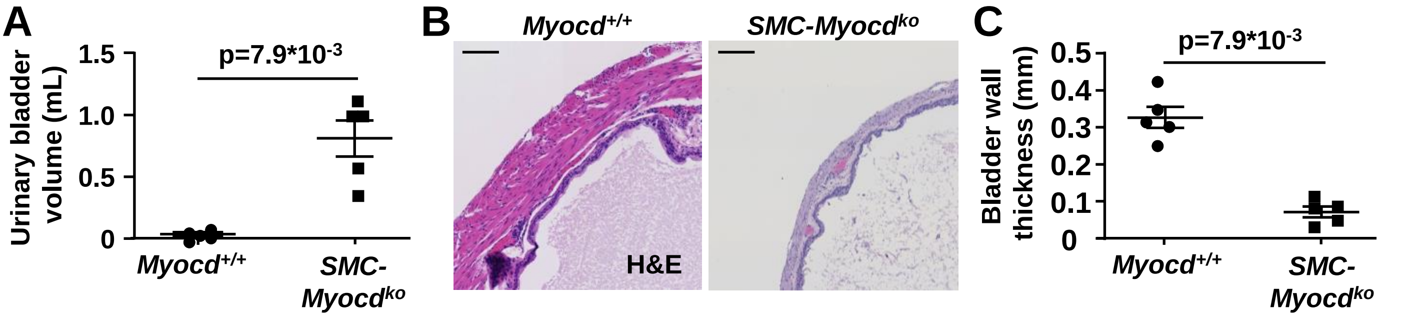

Figure S8. *SMC-Myocardin* deficiency induces bladder wall thinning within one week after induction of *Myocardin* deletion in SMCs. *Myocardin* deletion in SMCs was induced by 10 tamoxifen injections. *SMC-Myocd*^{ko} mice were sacrificed at 7 days after the last tamoxifen injection and were compared to *Myocd*$^{+/+}$ animals. (**A**) Urinary bladder volume. (**B**) The urinary bladder was isolated, sectioned, stained with haematoxylin-eosin (H&E). Scale bar represents 200 µm. (**C**) Bladder wall thickness was measured (n=5). Data are shown as mean ± SEM. *p*-values by Mann-Whitney U test are indicated.

Figure S9

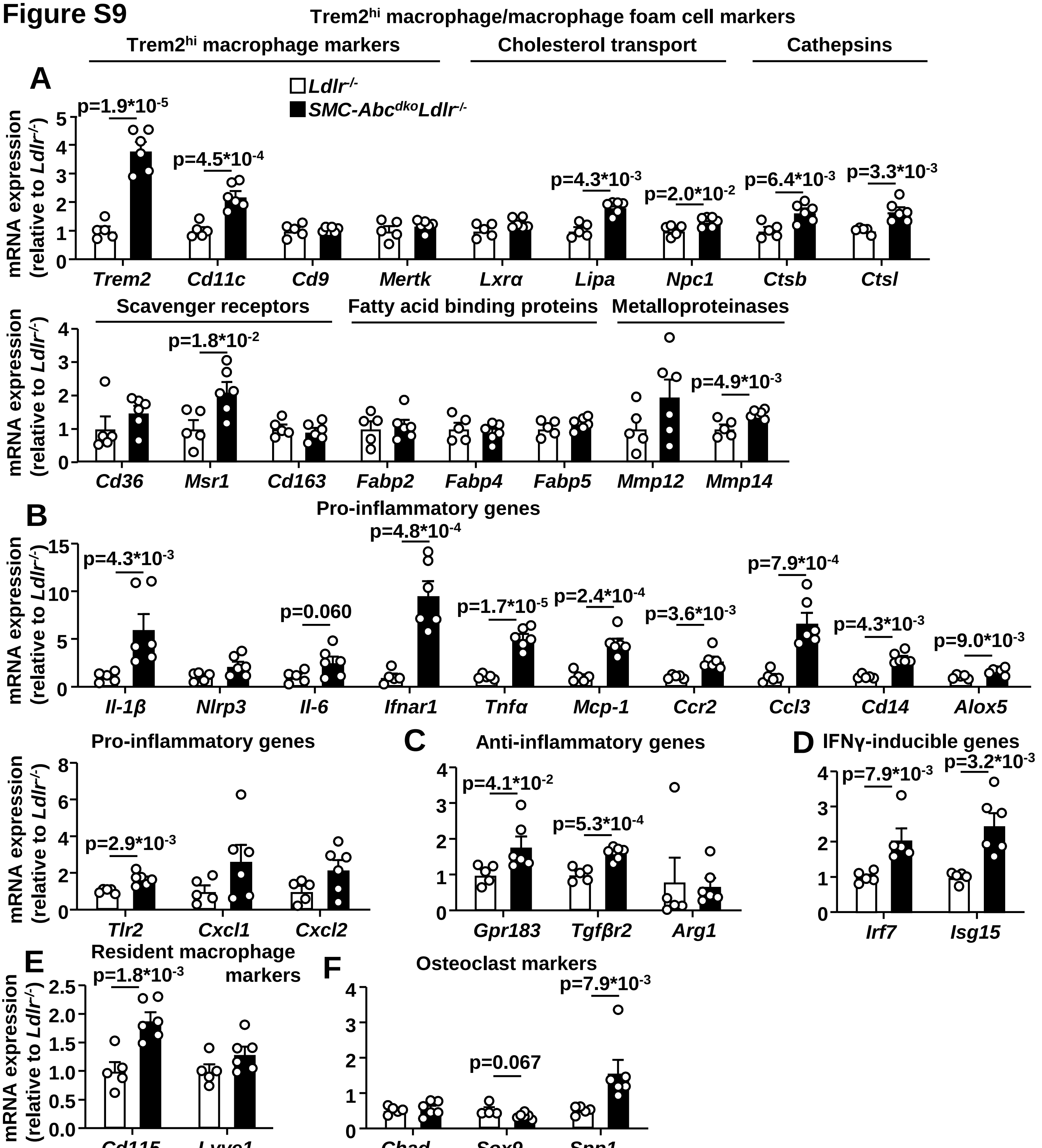

Figure S9. Effects of *SMC-Abca1/Abcg1* deficiency on mRNA expression in urinary bladder wall SMCs in WTD-fed *Ldlr*$^{-/-}$ mice. *Ldlr*$^{-/-}$ and *SMC-Abc*^{dko}*Ldlr*$^{-/-}$ mice were fed WTD for 6 weeks. The urinary bladder was isolated, digested, and mRNA expression of (**A**) Trem2^{hi} macrophage/macrophage foam cell markers, (**B**) pro-inflammatory genes, (**C**) anti-inflammatory genes, (**D**) IFNγ-inducible genes, (**E**) resident macrophage markers, and (**F**) osteoclast markers, was determined (*Ldlr*$^{-/-}$ (n=5, except for *Sox9,* where n=4 due to one outlier being excluded based on the ROUT test), *SMC-Abc*^{dko}*Ldlr*$^{-/-}$ (n=6, except for *Irf7* and *Spp1,* where n=5 due to one outlier being excluded based on the Grubb's test)). Data are shown as mean ± SEM. *p*-values (two tailed unpaired t-test, except for *Lipa, Il-1β, Cd14, Irf7, Sox9 and Spp1,* where statistical significance was tested by Mann-Whitney U test), are indicated.

Figure S10

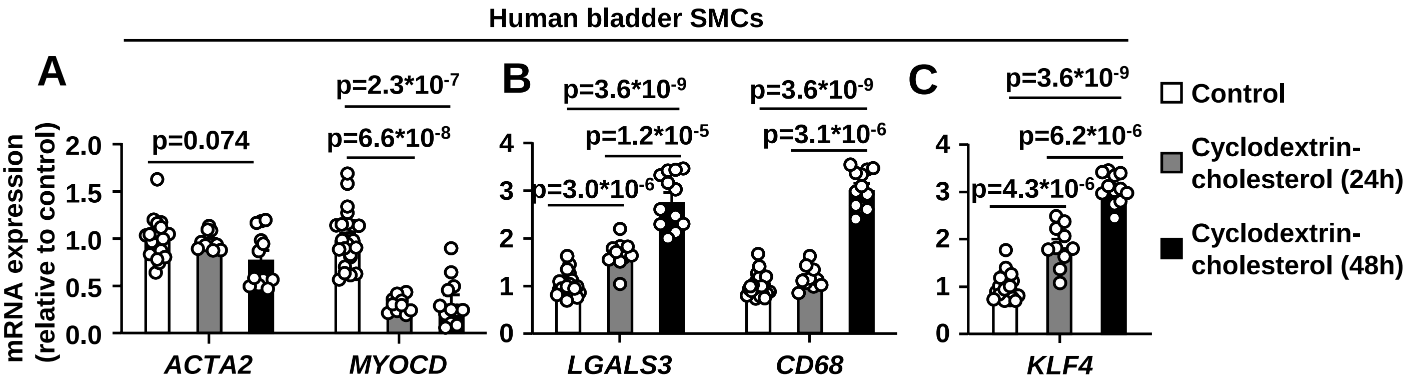

Figure S10. Cyclodextrin-cholesterol loading in human bladder SMCs induces loss of SMC contractile markers and gain of macrophage markers. Human bladder SMCs were incubated with or without 50 µg/mL cylodextrin-cholesterol for 24 or 48 hours. mRNA expression of (**A**) SMC markers, (**B**) macrophage markers, as well as (**C**) *Klf4* was determined (control (n=22), Cyclodextrin-cholesterol (24 h) (n=10), Cyclodextrin-cholesterol (48h) (n=12)). Data are shown as mean ± SEM. *p*-values by Mann-Whitney U test except for MYOCD, which was calculated by two-way tailed unpaired t-test, are indicated.

Figure S11

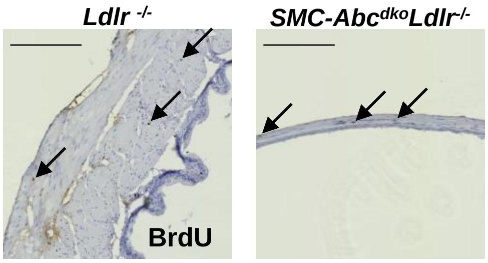

Figure S11. Low number of BrdU positive cells in the urinary bladder of *Ldlr*$^{-/-}$ and *SMC-Abc*^{dko}*Ldlr*$^{-/-}$ mice. *Ldlr*$^{-/-}$ and *SMC-Abc*^{dko}*Ldlr*$^{-/-}$ mice were fed WTD for 16 weeks. Mice were injected with Bromodeoxyuridine (BrdU) 48 and 24 hours intraperitoneally (50 mg/kg bodyweight per injection) prior to sacrifice. The urinary bladder was isolated, sectioned, and stained with anti-BrdU. Arrowheads indicate BrdU positive areas. Scale bar represents 200 µm.

Figure S12

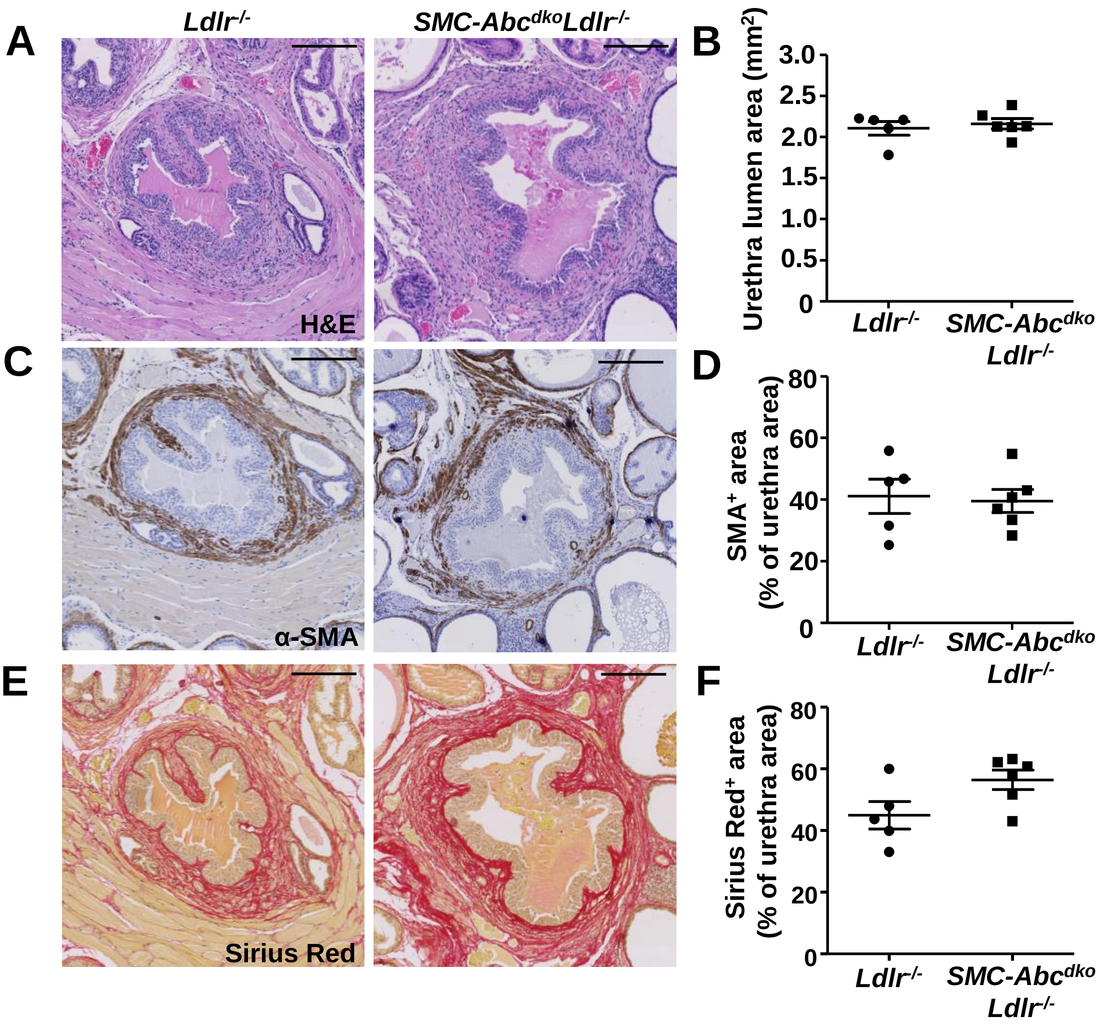

Figure S12. *SMC-Abca1/Abcg1* deficiency does not affect urethra lumen area and composition in WTD-fed *Ldlr$^{-/-}$* mice. Mice were fed WTD for 16 weeks. (**A**) The urethra was isolated, sectioned and stained with haematoxylin-eosin (H&E). (**B**) Quantification of lumen area of the urethra. (**C**) Sections were stained for α smooth muscle actin (α-SMA). (**D**) Quantification of SMA^{+} area as % of urethra area (**E**) Sections were stained with Sirius Red. (**F**) Quantification of Sirius Red^{+} area as % of urethra area (*Ldlr$^{-/-}$* (n=5), *SMC-Abc^{dko}Ldlr$^{-/-}$* (n=6)). Scale bar represents 200 µm. Data are shown as mean ± SEM.

Figure S13

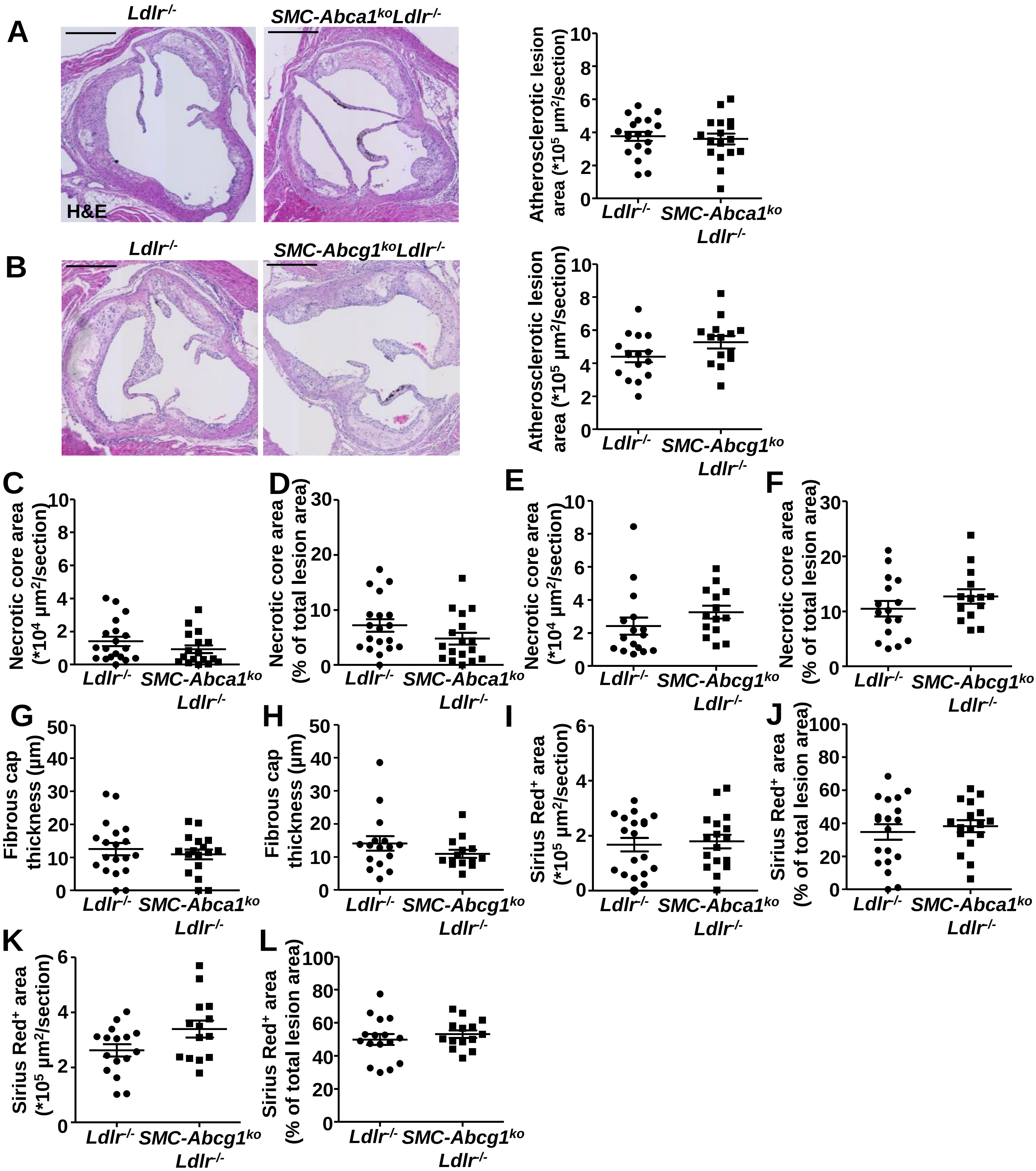
A
Ldlr-/-
SMC-Abca1koLdlr-/-
H&E
Atherosclerotic lesion area (*10^5 µm²/section)
B
SMC-Abcg1koLdlr-/-
C
Necrotic core area (*10^4 µm²/section)
D
Necrotic core area (% of total lesion area)
E
F
G
Fibrous cap thickness (µm)
H
I
Sirius Red+ area (*10^5 µm²/section)
J
Sirius Red+ area (% of total lesion area)
K
L

Figure S13 (Continued)

M

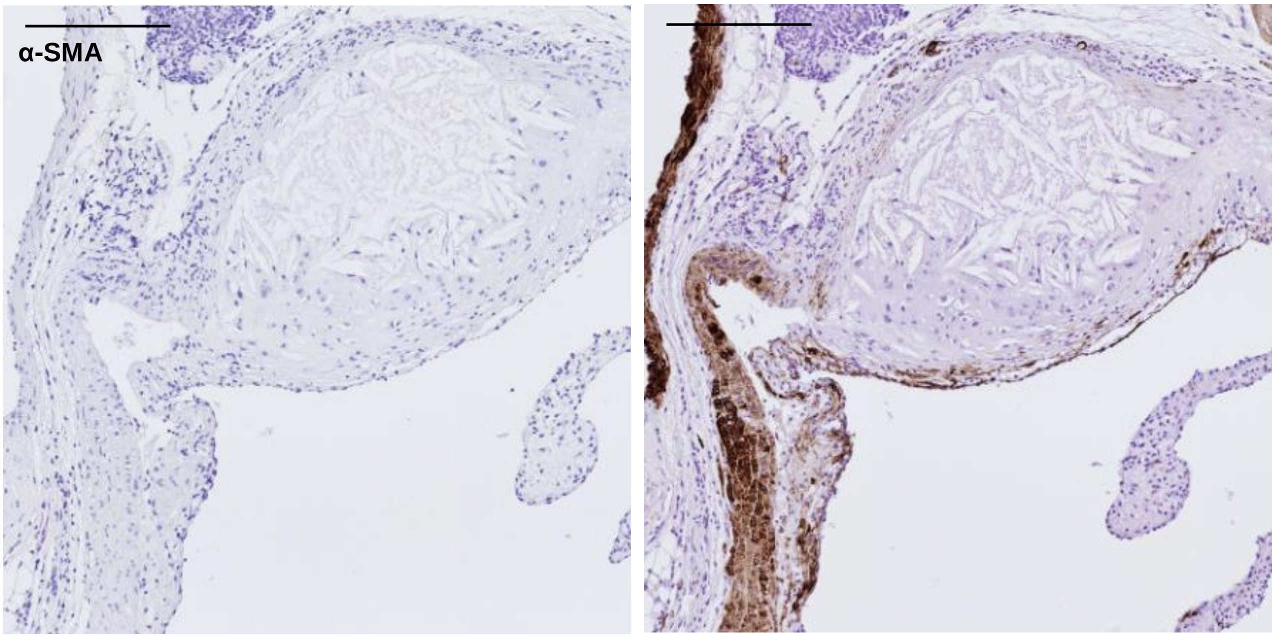

N

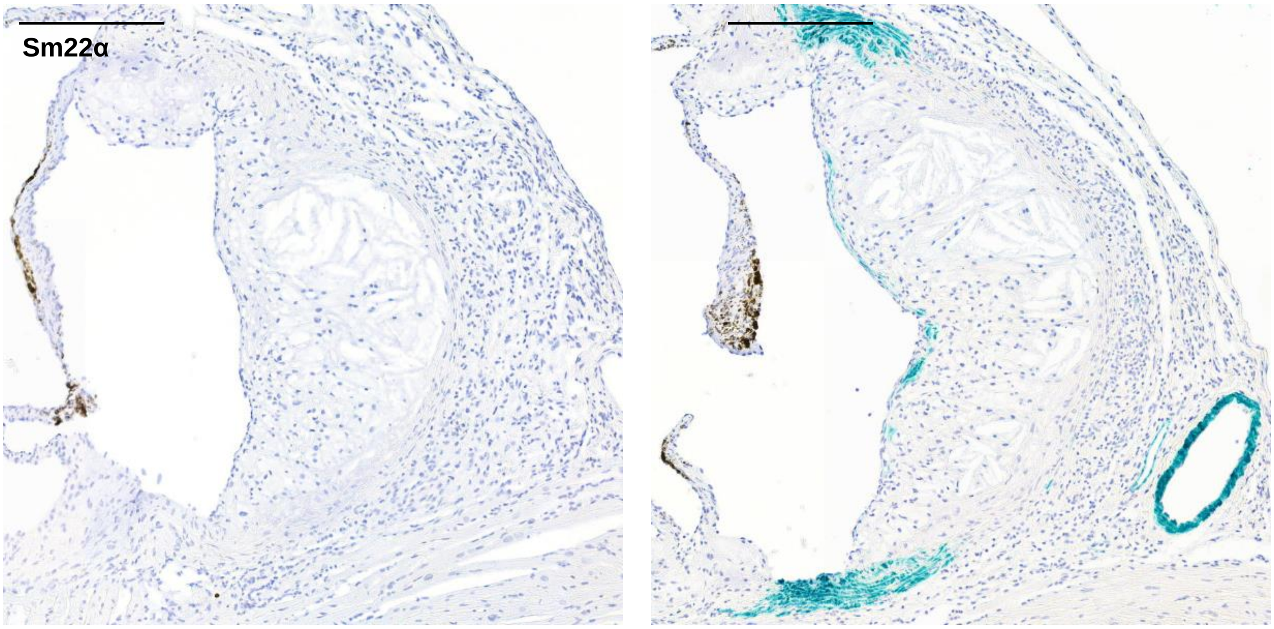

O

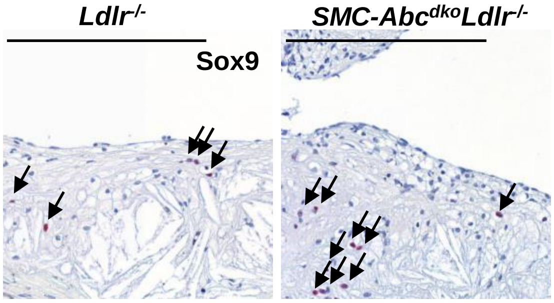

P

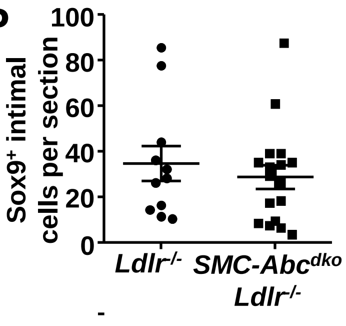

Q

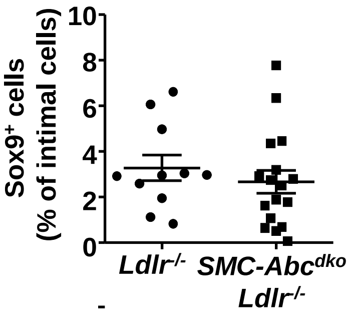

Figure S13 (Continued)

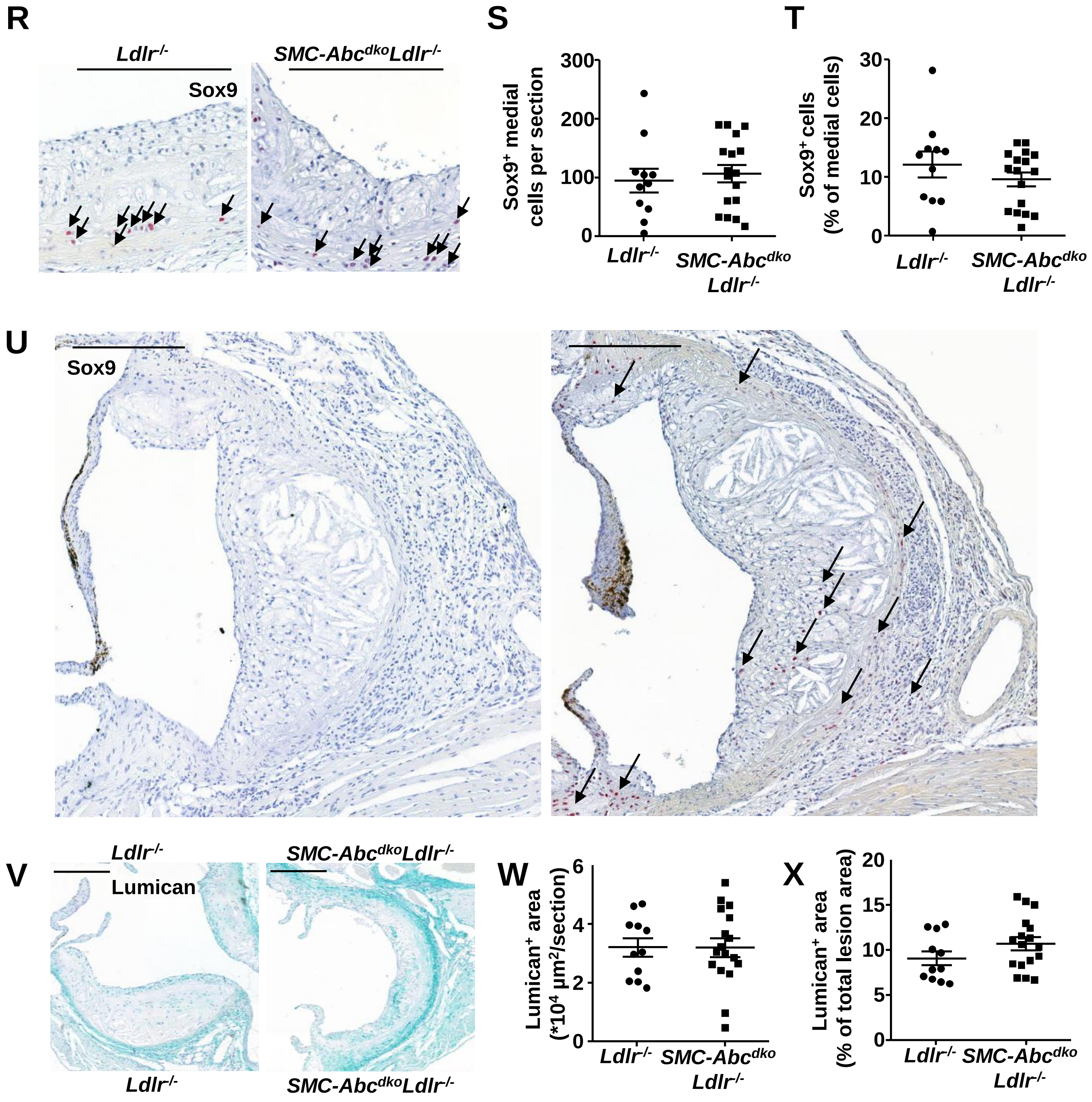

Figure S13 (Continued)

Y

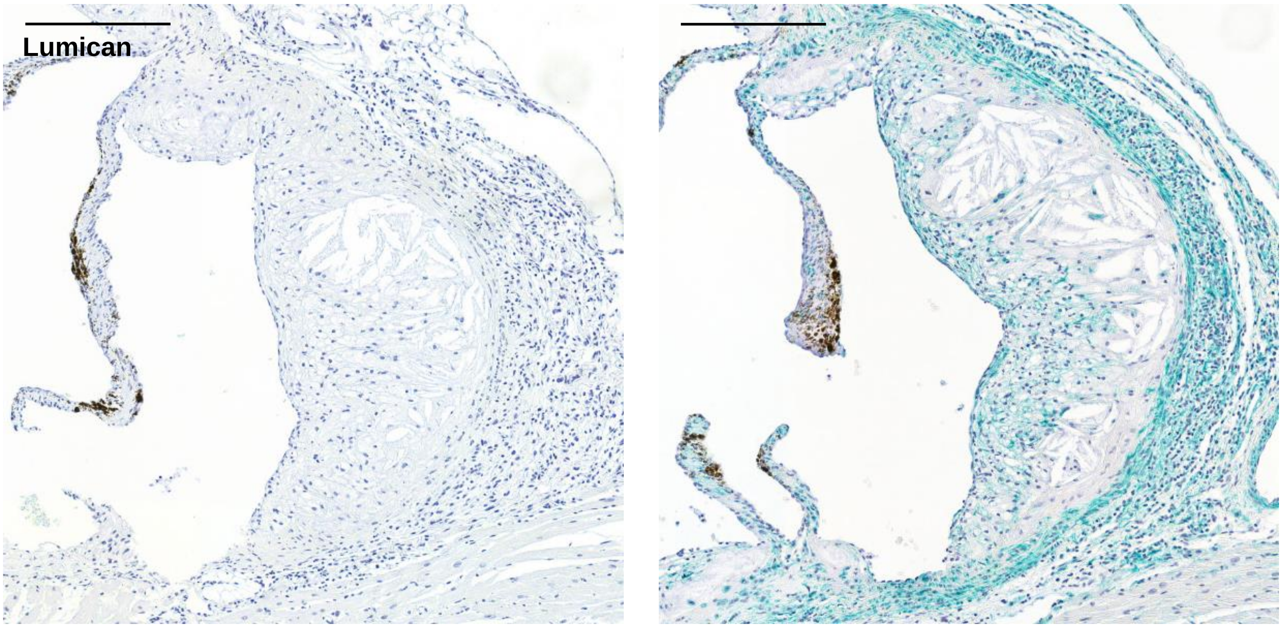

Figure S13. *SMC-Abca1 or* SMC *Abcg1* deficiency does not affect atherosclerotic lesion area, necrotic core area, or lesion composition in *Ldlr*$^{-/-}$ mice fed WTD. *Ldlr*$^{-/-}$, *SMC-Abca1*^{ko}*Ldlr*$^{-/-}$, *SMC-Abcg1*^{ko}*Ldlr*$^{-/-}$ and *SMC-Abc*^{dko}*Ldlr*$^{-/-}$ mice were fed WTD for 16 weeks. Hearts were isolated, sections were made of the aortic root and stained with haematoxylin-eosin (H&E). (**A**-**B**) Representative examples and quantification of atherosclerotic lesion area in *SMC-Abca1*^{ko}*Ldlr*$^{-/-}$ (n=17) (**A**), *SMC-Abcg1*^{ko}*Ldlr*$^{-/-}$ (n=14) (**B**) mice, and their *Ldlr*$^{-/-}$ (**A** (n=19), **B** (n=16)) controls. (**C**-**F**) Quantification of necrotic core area (**C**, **E**) and necrotic core area as % of total atherosclerotic lesion area (**D**, **F**) in *SMC-Abca1*^{ko}*Ldlr*$^{-/-}$ (n=17) (**C, E**), *SMC-Abcg1*^{ko}*Ldlr*$^{-/-}$ (n=14) (**D, F**) mice, and their *Ldlr*$^{-/-}$ (**C, D** (n=19), **E, F** (n=16)) controls. (**G**-**L**) Sections were stained with Sirius Red. Quantification of fibrous cap thickness (**G**-**H**), Sirius Red^{+} area (**I, K**), and Sirius Red^{+} area as % of total atherosclerotic lesion area (**J, L**) in *SMC-Abca1*^{ko}*Ldlr*$^{-/-}$ (n=17) (**G, I, J**), *SMC-Abcg1*^{ko}*Ldlr*$^{-/-}$ (n=14) (**H, K, L**) mice, and their *Ldlr*$^{-/-}$ (**G, I, J** (n=19), **H, K, L** (n=16)) controls. (**M**) Negative control for α-SMA staining (left, negative control without α-SMA primary antibody; right, adjacent section stained with α-SMA primary antibody). (**N**) Negative control for Sm22α staining (left, negative control without Sm22α primary antibody; right, adjacent section stained with Sm22α primary antibody). (**O-U**) Sections were stained with SRY-box transcription factor 9 (Sox9). (**O**) Representative examples of Sox9 staining in the intima. (**P, Q**) Quantification of Sox9^{+} intimal cells per section (**P**) and Sox9^{+} intimal cells as % of total intimal cells (**Q**). (**R**) Representative examples of Sox9 staining in the media. (**S, T**) Quantification of Sox9^{+} medial cells per section (**S**) and Sox9^{+} medial cells as % of total medial cells (**T**). (**U**) Negative control for Sox9 (left, negative control without Sox9 primary antibody; right, adjacent section stained with Sox9 primary antibody). (**V-Y**) Sections were stained for Lumican. (**V**) Representative examples of Lumican staining. (**W, X**) Quantification of Lumican^{+} area (**W**) and Lumican^{+} area as % of total atherosclerotic lesion area (**X**). (**Y**) Negative control for Lumican staining (left, negative control without Lumican primary antibody; right, adjacent section stained with Lumican primary antibody). (**P**-**T** and **W**-**X**) *Ldlr*$^{-/-}$ (n=11), *SMC-Abc*^{dko}*Ldlr*$^{-/-}$ (n=17). (**A**-**B**) Scale bar represents 400 µm. (**M**-**N, U-V, Y**) Scale bar represents 200 µm. (**O, R**) Scale bar represents 100 µm. Each data point represents an individual mouse. Data are shown as mean ± SEM.

Figure S14

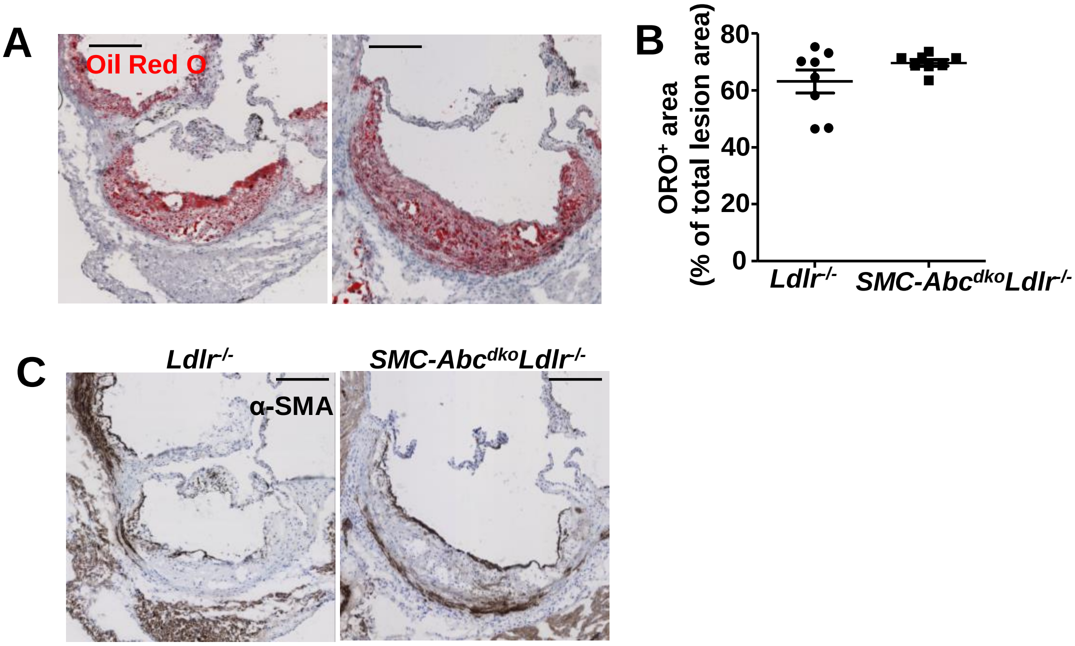

Figure S14. *SMC-Abca1/Abcg1* deficiency does not affect lipid accumulation in atherosclerotic lesions of *Ldlr*$^{-/-}$ mice fed WTD. Hearts from *Ldlr*$^{-/-}$ and *SMC-Abc*^{dko}*Ldlr*$^{-/-}$ mice fed WTD for 16 weeks were isolated, embedded in OCT compound, and frozen sections of the aortic root were prepared and sections were stained with Oil Red O (ORO). (**A**) Representative examples of ORO staining. (**B**) Quantification of Oil Red O^{+} area as % of total atherosclerotic lesion area. (n=8). (**C**) Sections adjacent to the ones used for Oil Red O staining were stained for α smooth muscle actin (α-SMA). Representative examples of α-SMA staining. (**A**,**C**) Scale bar represents 200 µm. (**B**) Each data point represents an individual mouse. Data are shown as mean ± SEM.

Figure S15

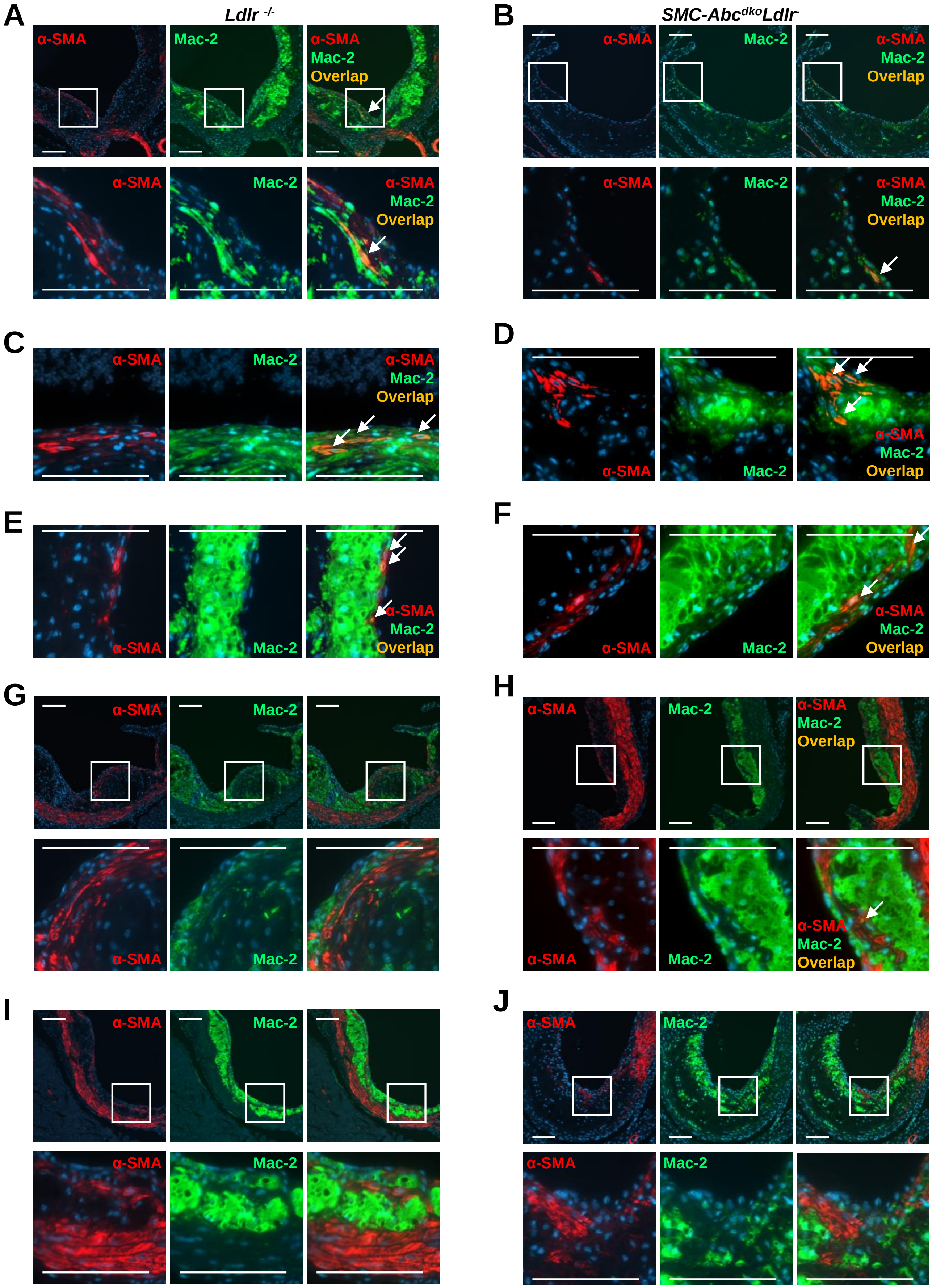

Figure S15 (Continued)

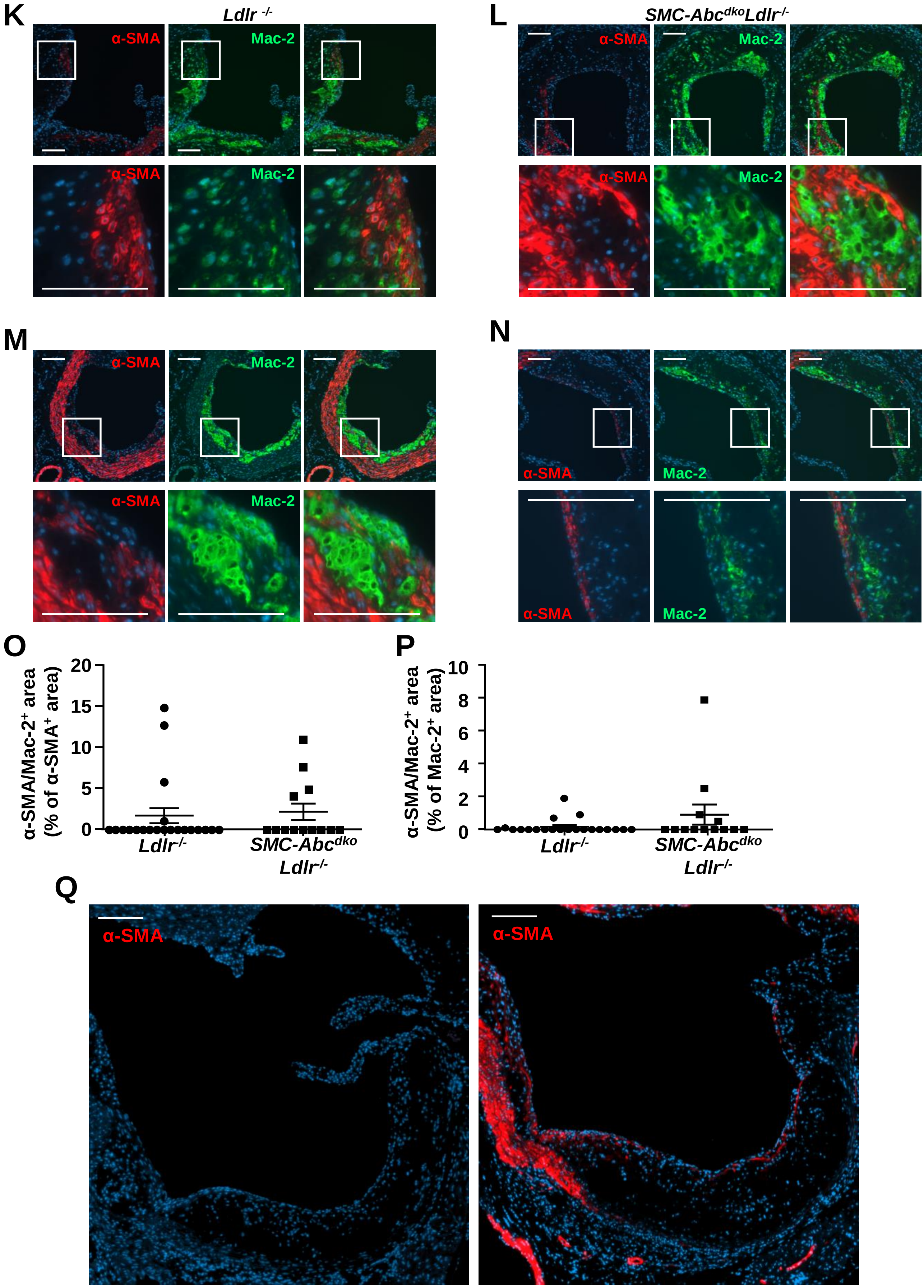

Figure S15. *SMC-Abca1/Abcg1* deficiency does not affect co-localization of α-SMA and Mac-2 in the aorta of *Ldlr*$^{-/-}$ mice fed WTD. *Ldlr*$^{-/-}$ and *SMC-Abc*^{dko}*Ldlr*$^{-/-}$ mice were fed WTD for 16 weeks. Hearts were isolated, sections were made of the aortic root, and stained for α-SMA, Mac-2, and DAPI. (**A**-**N**) Representative examples of (**A**-**F,H**) plaques with overlap of α-SMA and Mac-2 and (**G, I**-**N**) plaques with no overlap. Arrowheads indicate co-localization of α-SMA and Mac-2. (**O**-**P**) Quantification of area positive for α-SMA and Mac-2 as % of (**O**) α-SMA^{+} or (**P**) Mac-2^{+} area (*Ldlr*$^{-/-}$ (n=21), *SMC-Abc*^{dko}*Ldlr*$^{-/-}$ (n=13)). (**Q**) Negative control for α-SMA (left, negative control without α-SMA primary antibody; right, adjacent section stained with α-SMA primary antibody). Scale bar represents 100 µm. Each data point represents an individual mouse. Data are shown as mean ± SEM.

Figure S16

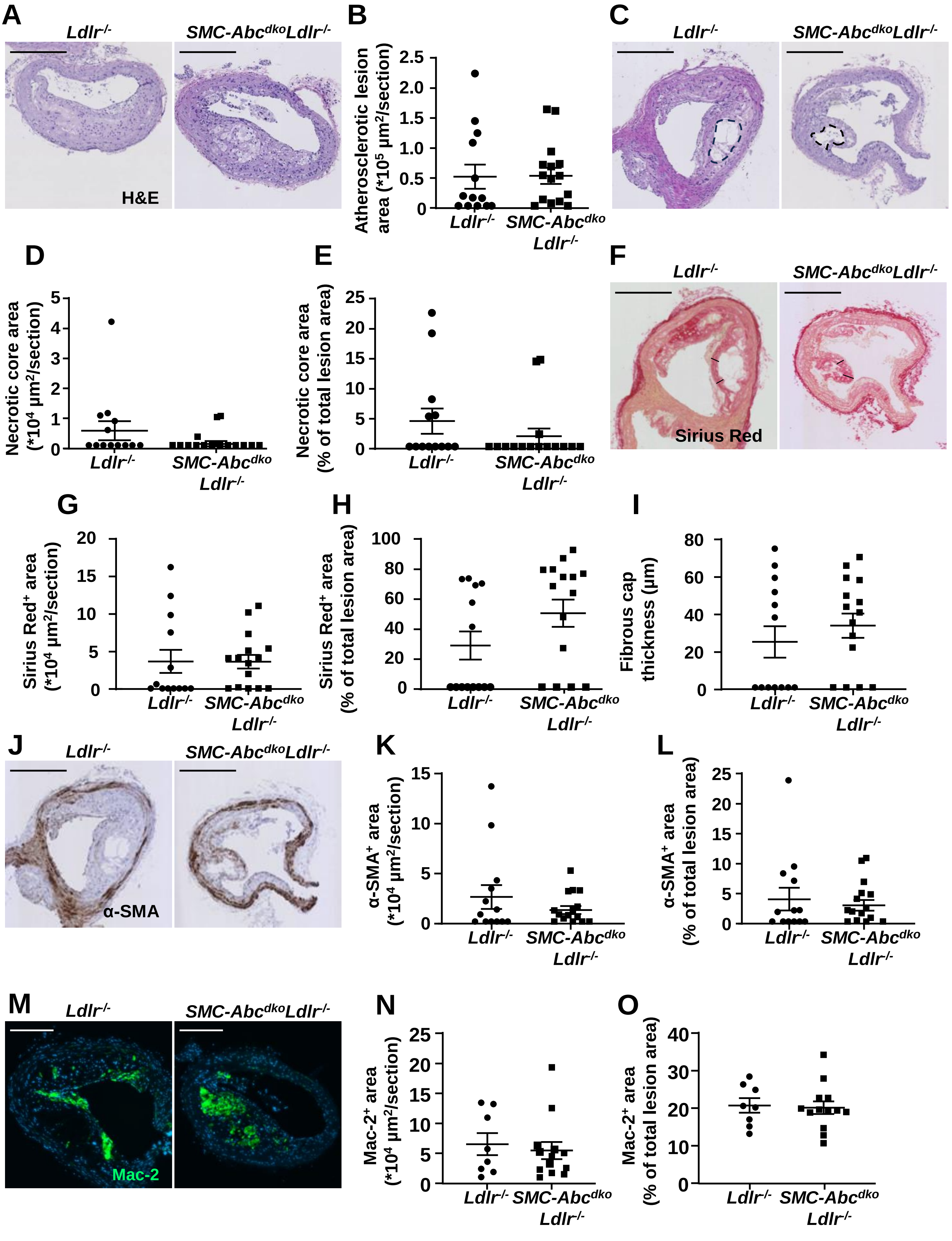

A
Ldlr-/-
SMC-Abcdko Ldlr-/-
H&E
B
Atherosclerotic lesion area (*10^5 µm^2/section)
2.5
2.0
1.5
1.0
0.5
0
Ldlr-/-
SMC-Abcdko Ldlr-/-
C
Ldlr-/-
SMC-Abcdko Ldlr-/-
D
Necrotic core area (*10^4 µm^2/section)
5
4
3
2
1
0
Ldlr-/-
SMC-Abcdko Ldlr-/-
E
Necrotic core area (% of total lesion area)
25
20
15
10
5
0
Ldlr-/-
SMC-Abcdko Ldlr-/-
F
Ldlr-/-
SMC-Abcdko Ldlr-/-
Sirius Red
G
Sirius Red+ area (*10^4 µm^2/section)
20
15
10
5
0
Ldlr-/-
SMC-Abcdko Ldlr-/-
H
Sirius Red+ area (% of total lesion area)
100
80
60
40
20
0
Ldlr-/-
SMC-Abcdko Ldlr-/-
I
Fibrous cap thickness (µm)
80
60
40
20
0
Ldlr-/-
SMC-Abcdko Ldlr-/-
J
Ldlr-/-
SMC-Abcdko Ldlr-/-
α-SMA
K
α-SMA+ area (*10^4 µm^2/section)
15
10
5
0
Ldlr-/-
SMC-Abcdko Ldlr-/-
L
α-SMA+ area (% of total lesion area)
25
20
15
10
5
0
Ldlr-/-
SMC-Abcdko Ldlr-/-
M
Ldlr-/-
SMC-Abcdko Ldlr-/-
Mac-2
N
Mac-2+ area (*10^4 µm^2/section)
25
20
15
10
5
0
Ldlr-/-
SMC-Abcdko Ldlr-/-
O
Mac-2+ area (% of total lesion area)
40
30
20
10
0
Ldlr-/-
SMC-Abcdko Ldlr-/-

Figure S16. *SMC-Abca1/Abcg1* deficiency does not affect atherosclerotic lesion area, necrotic core area, or lesion composition in the brachiocephalic artery of *Ldlr*$^{-/-}$ mice fed WTD. *Ldlr*$^{-/-}$ and *SMC-Abc*^{dko}*Ldlr*$^{-/-}$ mice were fed WTD for 16 weeks. The brachiocephalic artery was isolated, sectioned, and stained with haematoxylin-eosin (H&E) (**A, B**) Representative example (**A**) and quantification (**B**) of atherosclerotic lesion area. (**C**-**E**) Representative example (**C**) and quantification of necrotic core area (**D**) and necrotic core area as % of total atherosclerotic lesion area area (**E**). Black dashed lines indicate necrotic cores. (**F**-**I**) Sections were stained with Sirius Red. (**F**) Representative example of Sirius Red staining and quantification of fibrous cap thickness (**G**), collagen^{+} area (**H**) and collagen^{+} area as % of total atherosclerotic lesion area (**I**). Black lines indicate the fibrous cap. (**J**-**L**) Sections were stained for α smooth muscle actin (α-SMA). (**J**) Representative examples of α-SMA staining. (**K, L**) Quantification of SMA^{+} area (**K**) and SMA^{+} area as % of total atherosclerotic lesion area (**L**). (**M**-**O**) Sections were stained for Mac-2 (Lgals3). (**M**) Representative examples of Mac-2 staining. (**N, O**) Quantification of Mac-2^{+} (Lgals3^{+}) area (**N**) and Mac-2^{+} area as % of total atherosclerotic lesion area (**O**) (*Ldlr*$^{-/-}$ (n=13), *SMC-Abc*^{dko}*Ldlr*$^{-/-}$ (n=15)). Scale bar represents 200 µm. Each data point represents an individual mouse. Data are shown as mean ± SEM.

Figure S17

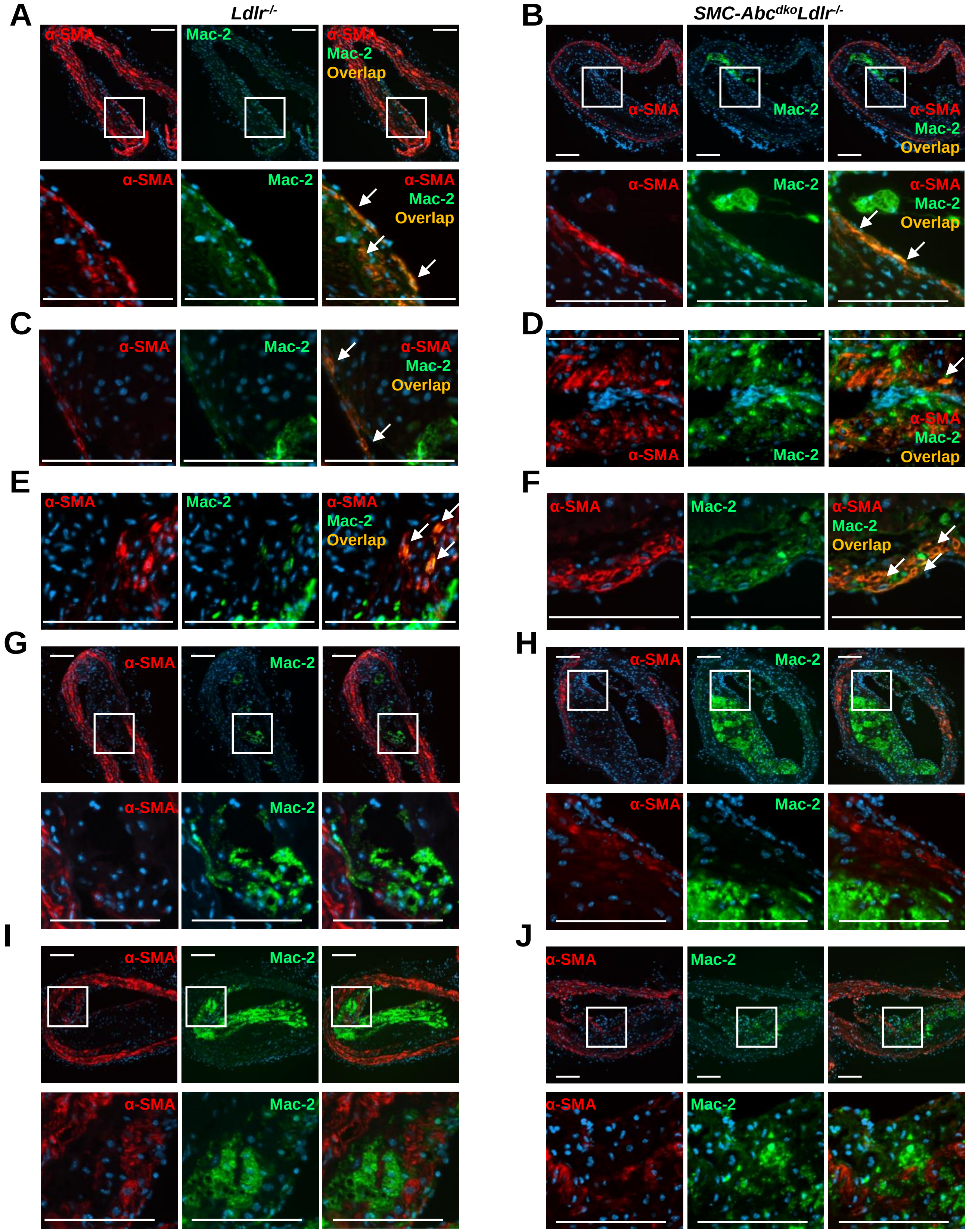

Figure S17 (Continued)

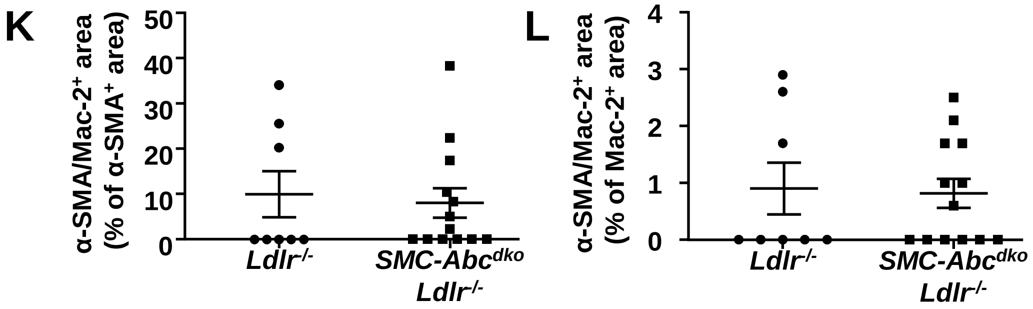

Figure S17. *SMC-Abca1/Abcg1* deficiency does not affect colocalization of α-SMA and Mac-2 in the brachiocephalic arteries of *Ldlr$^{-/-}$* mice fed WTD. *Ldlr$^{-/-}$* and *SMC-Abc^{dko}Ldlr$^{-/-}$* mice were fed WTD for 16 weeks. The brachiocephalic artery was isolated, sectioned, and stained for α-SMA, Mac-2, and DAPI. (**A**-**J**) Representative examples of (**A**-**F**) regions overlapping α-SMA and Mac-2 and (**G**-**J**) regions with no overlap. Arrowheads indicate co-localization of α-SMA and Mac-2. (**K**-**L**) Quantification of overlapping SMA and Mac-2 area as % of (**K**) SMA^{+} and (**L**) Mac-2^{+} area (*Ldlr$^{-/-}$* (n=8), *SMC-Abc^{dko}Ldlr$^{-/-}$* (n=13)). Scale bar represents 100 µm. Each data point represents an individual mouse. Data are shown as mean ± SEM.
